# The Risk of Preterm Birth in Women With Periodontitis: A Systematic Review and Meta‐Analysis

**DOI:** 10.1111/idh.70001

**Published:** 2025-10-02

**Authors:** Dimitris Sokos, Dagmar Else Slot, Berna Dogan, Sergio Bizzarro

**Affiliations:** ^1^ Department of Periodontology, Academic Center for Dentistry Amsterdam (ACTA) University of Amsterdam and Vrije Universiteit Amsterdam Amsterdam the Netherlands

**Keywords:** gestation, meta‐analysis, odds ratio, periodontal medicine, periodontitis, pregnancy, preterm birth, systematic review

## Abstract

**Aim:**

Previous studies have explored the association between periodontitis and preterm birth (PTB), with conflicting results primarily due to variability in methodology, statistical analyses and the case definitions of both conditions. This study aimed to synthesise critically the available scientific evidence of observational studies that evaluate the risk of PTB in pregnant women with or without periodontitis.

**Material and Methods:**

MEDLINE‐PubMed and Cochrane databases were searched from their inception through June 2025 to identify eligible studies evaluating the incidence of PTB in women with periodontitis compared to those without. Inclusion required that periodontitis be defined through full‐mouth clinical examination, assessing probing pocket depth in combination with clinical attachment loss and/or radiographic alveolar bone loss. The risk of bias was assessed. Descriptive analysis, and when feasible, meta‐analysis (MA) and trial sequential analysis (TSA) were performed. Sub‐analyses were conducted based on the risk of bias analysis, study design, geographical area, periodontitis case definition, smoking, health or country social economic status, history of urinary tract infections and the number of evaluated teeth. The total body of evidence was graded.

**Results:**

A total of 723 unique papers were identified, and 11 eligible studies were included. The descriptive analysis showed that seven of the eleven studies present a significant association between PTB and periodontitis. Confounding variables on personal, medical and environmental aspects lowered the risk. The crude overall MA based on 11 studies resulted in a small effect, odds ratio (OR: 2.38 [95% CI: 1.78; 3.18], *p* < 0.00001). The TSA showed that the required number of events was reached, and the type I error is ruled out.

**Conclusion:**

There is moderate certainty that pregnant women with periodontitis compared to pregnant women without periodontitis have a small risk of PTB.

**Trial Registration:**

PROSPERO number: CRD42022327501

## 1 Introduction

Preterm birth (PTB) takes place when infants are born alive before completing of the 37th week of gestation. Annually, around 15 million babies are born premature [1]. PTB complications are the main cause of deaths in children under 5 years old. Every year, approximately 1.055 million deaths are attributed to prematurity [2]. In addition, the infants who survive are at a high risk of long‐term morbidity, such as neurologic and developmental disabilities [3]. Therefore, PTB is considered a major global health issue [1]. Several risk factors, related to maternal characteristics, reproductive history and pregnancy characteristics, have been associated with PTB [1, 4, 5]. Also, extragenital infections and inflammatory diseases, such as periodontitis, are suggested to be associated with PTB [4, 5].

Periodontitis is a multicausal, complex, chronic inflammatory disease of the periodontium, the supporting tissues of the teeth. It is characterised by an aberrant immune response to the dental biofilm [6]. It is the sixth most common human disease, with a prevalence of 45%–50% worldwide. Severe periodontitis affects approximately 7.4% of the world's population [7]. Moreover, periodontitis is associated with increased levels of inflammation biomarkers in the bloodstream [8, 9, 10]. In the scientific literature, two potential mechanisms are suggested for explaining the relation between PTB and periodontitis: a direct and an indirect pathway. Currently, scientific evidence supports mainly the direct pathway due to the bacterial transfer from the oral environment to the foetal‐placental unit [11].

Several observational studies, systematic reviews (SR) and meta‐analyses (MA) have investigated the association between periodontitis and PTB with conflicting results [12, 13, 14, 15, 16]. These discrepancies are largely attributable to differences in the study designs, the sample sizes, the statistical analyses, the adjustment for potential confounding factors, and the periodontitis case definitions [12, 17, 18]. In the light of these considerations and the publication of new studies on this topic, the aim of this SR and MA was to assess, summarise and synthesise the available scientific evidence derived from observational studies on the risk of PTB in pregnant women with periodontitis compared to those without periodontitis.

## 2 Material and Methods

This SR complied with the *Cochrane Handbook for Systematic Reviews* [19] and the guideline for Meta‐Analysis of Observational Studies in Epidemiology (MOOSE) [20]. A protocol was developed a priori following the initial team discussion. Ethical approval under the reference number 2021‐50260 was granted by the ACTA ethical committee, and the study has been registered with the International Prospective Register of Systematic Reviews (PROSPERO) by number CRD42022327501.

### 2.1 Focused Question and Hypothesis

A precise review question was formulated utilising the population, exposure, comparison, outcomes and study (PECOS) framework as follows [19]:

- In pregnant women (population) with periodontitis (exposure) compared to those without (comparison), what is the risk of PTB (outcome) as established from observational investigations (studies)?

Due to the potential link between periodontitis and PTB, it was hypothesised that there is a higher risk of PTB in pregnant women with periodontitis as compared to those without.

### 2.2 Search Strategy

A structured search strategy was designed to retrieve all relevant studies that evaluate the PTB events among pregnant women with periodontitis as compared to those without. The search was designed by three reviewers (B.D., D.S. and S.B.). The National Library of Medicine in Washington, DC (MEDLINE‐PubMed) and Cochrane Central were searched from the inception of this study through June 2025 for appropriate papers that answer the focused question. Table 1 provides details regarding the search approach employed.

**TABLE 1:** The following strategy was used in the search and customised to the database being searched.

{< Exposure: [MeSH] (periodontal disease) OR (periodontitis) OR [textwords] (periodontal disease) OR (periodontitis) OR (periodontal diseas*) > AND < Outcome: [MeSH] (premature birth) OR (preterm birth) OR (preterm labor) [textwords] (premature birth) OR (preterm birth) OR (preterm labor) >}

*Note:* The asterisk (*) was used as a truncation symbol.

### 2.3 Screening and Selection

Two blinded reviewers (B.D. and D.S.) conducted the screening and selection process. After eliminating duplicates, the remaining records underwent an initial screening based on their titles and abstracts using Rayyan [21]. Subsequently, articles were selected for full‐text reading if they potentially met the inclusion criteria or if the information provided by the title and abstract was not sufficient to make a final decision. Additionally, the reference lists of the articles chosen for full‐text reading were manually searched to identify any additional articles that might be relevant.

Publications were considered eligible based on the predetermined inclusion criteria:

- Full‐text article available in English.
- Observational studies: cohort, case–control or cross‐sectional studies.
- Studies conducted with pregnant women.
- Evaluating a group with periodontitis and a group without periodontitis.
  ○ Definition of periodontitis, based on full‐mouth clinical examination, evaluating the amount of
    - probing pocket depth (PPD) in combination with
    - clinical attachment loss (CAL) AND/OR radiographic examination evaluating the amount of bone loss.
- Reported outcome: PTB
  ○ Definition of PTB when delivery took place before 37 weeks of pregnancy [1] and confirmed after clinical examination from a health care professional.

Any disagreement about the eligibility of studies between the two reviewers was resolved after additional discussion. If disagreement persisted, a third reviewer, S.B., was consulted, whose judgement was considered as decisive. Thereafter, all the selected studies identified and included for this SR were proceeded with for data extraction and estimation of the risk of bias. If necessary, corresponding authors were inquired of through email for additional information respecting those studies which met the inclusion criteria but did not provide sufficient or clear data to be incorporated in the analysis.

### 2.4 Methodological Quality Assessment

Two reviewers (B.D. and D.S.) independently scored the individual methodological qualities of the included studies utilising the Newcastle‐Ottawa scale (NOS) [22] for cohort and case–control, and its adaptation for the cross‐sectional studies [23]. Any disagreement in the assessment was resolved by a third reviewer (SB).

The NOS contains seven items for the cross‐sectional studies and eight items for the case–control and cohort studies. These items are categorised into three domains including selection, comparability and—depending on the study type—outcome (cohort and cross‐sectional studies) or exposure (case–control studies). For each item, a series of response options is provided. A star system was used to allow a semi‐quantitative assessment of study quality. The highest quality studies were awarded a maximum of one star for each item, except for the items related to comparability in all study designs, and the assessments of the exposure and outcome in the cross‐sectional studies, which allowed the assignment of two stars. The NOS ranges from zero up to nine stars for the case–control and cohort studies and from zero to ten stars for the cross‐sectional studies.

The judgements within each domain were carried forward to an overall risk of bias. A study with at least eight stars was classified as having a low risk of bias. Moderate risk of bias was assigned when the study scored six or seven stars. A study was classified as having a high risk of bias when it scored five stars or less.

### 2.5 Data Extraction

Independent data extraction was performed by two reviewers (B.D. and D.S.) by means of a predefined and custom‐designed standardised data extraction form. Disagreement between the reviewers was solved through discussion and consensus. If disagreement persisted, a third reviewer (S.B.) was consulted; this judgement was decisive. From the eligible papers, the following information was obtained: (1) primary researcher and year of publication, (2) location of the study, (3) design of the study, (4) age of the participants, (5) sample size, (6) periodontitis definition, (7) preterm birth definition, (8) outcome of PTB by absolute or relative numbers and (9) conclusions of the original authors. If multiple case definitions or thresholds were presented for periodontitis or PTB, only those which fulfilled the inclusion criteria were extracted. The definition most often used in other included studies was applied for data analysis.

### 2.6 Assessment of Clinical and Methodological Heterogeneity

The factors used to assess the clinical heterogeneity were the following: characteristics of participants (age, continent of origin, health status, smoking, history of urinary tract infection, socio‐economic status of the country of origin [24]), number of participants, details on definitions of PTB and periodontitis. The factors used to evaluate the methodological heterogeneity included study design details and the number of examined teeth per participant [25].

### 2.7 Data Analysis

A descriptive data presentation was utilised for all included studies to summarise the findings. In addition, the absolute number and percentage of pregnant women with PTB among those with and without periodontitis were extracted and calculated for each study. Based on this, the overall number and percentage of PTB events within each group were weighed based on the study population.

A meta‐analysis was performed comparing the number of PTB events among pregnant women with and those without periodontitis utilising Review Manager version 5.4 [26]. From the data, the Odds‐Ratio (OR) with its associated 95% confidence interval (CI) and *p*‐value were calculated; *p*‐values ≤ 0.05 were considered as significant. When there were four or more comparisons to be analysed, a random‐effects model (REM) was used. If there were less than four studies, a fixed‐effects model (FEM) analysis was conducted [19, 27]. The calculated OR was interpreted according to Chen et al. [28]: < 1.68 as none to very small, ≥ 1.68 as small, ≥ 3.47 as medium and ≥ 6.71 as large.

Statistically, heterogeneity was tested by the Chi‐squared test and *I* ^2^ statistic. A Chi‐squared test resulting in a *p* < 0.1 was considered an indication of significant statistical heterogeneity. As a sign of the possible magnitude of inconsistency across studies, an *I* ^2^ statistic of 0%–40% indicates negligible levels of heterogeneity. An *I* ^2^ statistic of 30%–60% indicates moderate heterogeneity, and an *I* ^2^ statistic of 50%–90% indicates substantial heterogeneity. An *I* ^2^ statistic greater than 75% was further assessed with subgroup or sensitivity analysis [25, 29]. Sensitivity analyses were undertaken to evaluate the effect of excluding studies based on specific aspects in the domain of clinical or methodological heterogeneity.

Publication bias testing was utilised as proposed by Egger et al. [30]. If the meta‐analysis included enough trials (at least 10), a funnel plot was used as a visual tool to assess publication bias. The presence of asymmetry in the inverted funnel is suggestive of publication bias [19]. When no clear asymmetry in the funnel plot was found, the Egger's test was applied.

TSA was employed to estimate the type Ι error risk. It is performed for all the included studies and for those with a low risk of bias assessment. The required information size (RIS) and trial sequential monitoring boundaries (TSMB) for benefit or futility were computed. The RIS was determined considering a type Ι error risk of *α* = 5% and a type ΙΙ error risk of *β* = 0.20, with a statistical test power of 80%. RIS was adjusted for heterogeneity and multiple comparisons. The Lan‐DeMets version [31] of the O'Brien‐Fleming function [32] was utilised to assess the TSMBs. The incidence rates in the exposed and unexposed arms used for the TSA were calculated based on the data obtained from the included studies. TSA was conducted. TSA software version 0.9.5.10 Beta (Copenhagen Trial Unit, Copenhagen, Denmark) was used [33, 34, 35, 36].

### 2.8 Grading the Body of Evidence

Two reviewers (D.S. and D.E.S.) rated the quality of the evidence and the strength of the recommendations according to the following aspects: study limitations, inconsistency of results, indirectness of evidence, imprecision and publication bias by utilising the Grading of Recommendations Assessment, Development and Evaluation (GRADE) [37], which provides a systematic approach for considering and reporting each of these factors. An overall rating of confidence in effect estimates was considered critical for the final recommendation [38]. Any disagreement between the two reviewers was resolved after additional discussion. If a disagreement persisted, the judgement of a third reviewer (SB) was considered as decisive.

## 3 Results

### 3.1 Search and Selection Results

**FIGURE 1:**
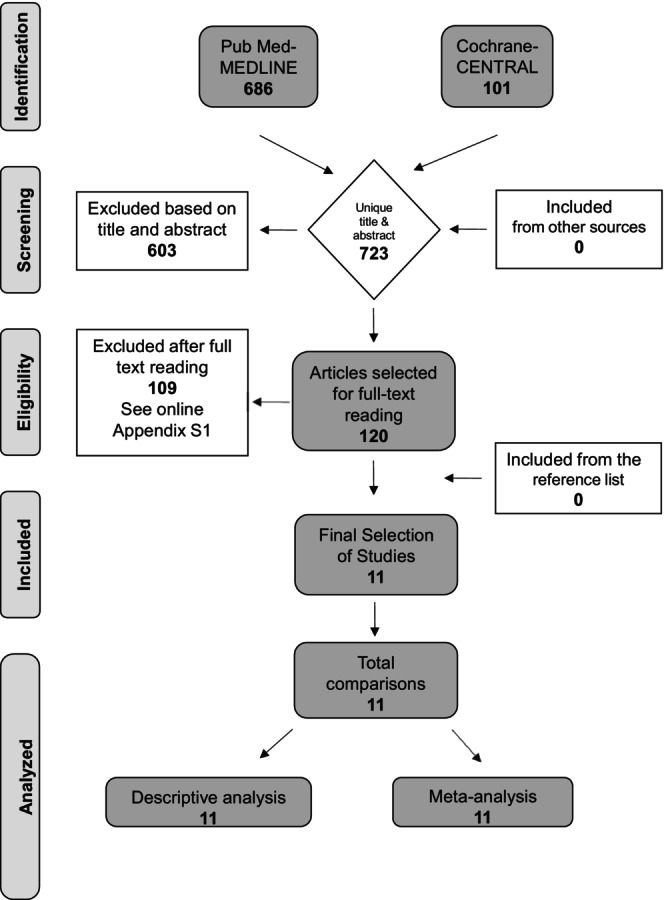
Search and selection results.

The electronic search of the MEDLINE‐PubMed and Cochrane‐CENTRAL resulted in 723 unique publications. After detailed screening, finally, 11 studies were selected and processed for further data extraction (for details see Figure 1 and Online Appendix S1).

### 3.2 Assessment of Clinical Heterogeneity

Table 2 presents an overview of the included studies, their design, the characteristics of the study populations, the case definitions of periodontitis and PTB, and the original author's conclusions.

**TABLE 2:** Overview of studies proceeded for data extraction.

Study ID, 1st author, year	Study design	Age range in years periodontitis/non‐periodontitis mean age (SD) in years	Definition		Original author's conclusion
			Periodontitis	PTB	
Country	Sample size				
I. Pockpa et al. 2022 [39] Ivory Coast	Cohort 338	15–50 NA	Interdental CAL at ≥ 2 non‐adjacent teeth OR Buccal or oral CAL and PPD ≥ 3 mm at ≥ 2 teeth	< 37 weeks	Periodontitis is an additional risk factor for PTB in Ivory Coast.
II. Caneiro et al. 2020 [13] Spain	Cohort 158	NA Periodontitis: 31.88 (± 4.38) Non‐periodontitis: 31.91 (± 4.21)	Interdental CAL at ≥ 2 non‐adjacent teeth OR Buccal or oral CAL and PPD ≥ 3 mm at ≥ 2 teeth	< 37 weeks	No statistical association between periodontitis stage II, grade B and PTB. 1‐week delivery and a 140 g weight difference between the groups could be amplified with a larger and more advanced periodontitis group. The association between periodontitis and PTB should be further explored in interventional studies to establish whether it is causal or incidental.
III. Pérez‐Molina et al. 2019 [40] Mexico	Case–control 1029	NA NA	≥ 4 teeth with ≥ 1 site with ≥ 4 mm PPD and clinical loss of bone or soft tissue ≥ 3 mm	< 37 weeks	Periodontitis in pregnancy is an independent risk factor for PTB.
IV. Martínez‐Martínez et al. 2016 [41] Mexico	Cross‐sectional 70	20–35 NA	D1: PPD ≥ 3 mm and CAL ≥ 2 mm in 30% of probed sites D2: ≥ 4 teeth with ≥ 1 site(s) with PPD ≥ 4 mm and CAL ≥ 3 mm OR 1 tooth with 1 site with PPD and CAL ≥ 4 mm	< 37 weeks	PTB is a multifactorial condition. Periodontitis and periodontopathogens are not sufficient to trigger PTB.
V. Macedo et al. 2014 [42] Brazil	Case–control 296	18–40 NA	D1: ≥ 4 teeth with ≥ 1 site PPD ≥ 4 mm and CAL ≥ 3 mm at the same site D2: ≥ 1 site with PPD and CAL of ≥ 4 mm	< 37 weeks	Periodontitis defined according to Definition 2 and unfavourable oral health‐related behaviour were factors associated with PTB.
VI. Kumar et al. 2013 [43] India	Cohort 193	18–31 Periodontitis: 22.32 (± 2.79) Non‐periodontitis: 22.32(± 2.75)	CAL and PPD ≥ 4 mm in ≥ 1 site(s)	< 37 weeks	Maternal periodontitis is associated with an increased risk of pre‐eclampsia, intrauterine growth restriction, PTB and LBW infants.
VII. Martinez de Teyada et al. 2012 [44] Switzerland	Case–control 429	NA NA	Moderate: ≥ 2 interproximal sites with CAL ≥ 4 mm, not on the same tooth, or ≥ 2 interproximal sites with PPD ≥ 5 mm, not on the same tooth Severe: ≥ 2 interproximal sites with CAL ≥ 6 mm, not on the same tooth, and ≥ 1 interproximal site with PPD ≥ 5 mm	≥ 22 and ≤ 34^6/7^ weeks	Early preterm delivery is associated with periodontitis when the USA consensus definitions are used. The European definitions revealed inadequate for the study population because of the lack of discrimination power.
VIII. Piscoya et al. 2012 [45] Brazil	Case–control 718	NA NA	≥ 4 teeth with PPD ≥ 4 mm and CAL ≥ 3 mm at the same site	< 37 weeks	Periodontitis is strongly associated with prematurity, indicating necessity for regular periodontal investigation and treatment during pregnancy.
IX. Agueda et al. 2008 [46] Spain	Cohort 1296	18–40 NA	≥ 4 teeth with ≥ 1 site with PPD ≥ 4 mm and CAL ≥ 3 mm at the same site.	< 37 weeks	The factors involved in many cases of adverse pregnancy outcomes have not still been identified, although systemic infections may play a role. This study found a modest association between periodontitis and PTB. Further research is required to establish whether periodontitis is a risk factor for PTB and/or LBW.
X. López et al. 2002 [14] Chile	Cohort 639	18–35 Periodontitis: 27.1 (± 4.3) Non‐periodontitis: 24.1 (± 4.6)	≥ 4 teeth with PPD ≥ 4 mm and CAL ≥ 3 mm at the same site	< 37 weeks	Periodontitis is an independent risk factor for PLBW. Periodontitis affords >three‐fold increase in the risk for PTB and LBW. Adverse pregnancy outcomes are frequently associated with potentially correctable lifestyles, or with infectious diseases that, like periodontitis, can be eliminated prior to or during pregnancy.
XI. Offenbacher et al. 2001 [15] USA	Cohort 812	NA NA	≥ 1 site with PPD > 3 mm and CAL > 2 mm	< 37 weeks	Maternal periodontal disease and incident progression are significant contributors to obstetric risk for preterm delivery. The results underscore the need for further consideration of periodontal disease as a potentially new and modifiable risk for PTB.

Abbreviations: CAL, clinical attachment loss; LBW, low birth weight; NA, not available; PLBW, preterm low birth weight; PPD, probing pocket depth; PTB, preterm birth; USA, United States of America.

The total number of participants was 5978. Three studies were conducted in South America [14, 42, 45], three in Europe [13, 44, 46], three in North America [15, 40, 41], one in Africa [39] and one in South Asia [43]. Among these, four [13, 15, 44, 46] were carried out in developed countries and seven in developing countries [14, 39, 40, 41, 42, 43, 45].

The reported age range was from 15 to 50, and the mean age varied from 22 up to 31. Age information was not provided in two studies [44, 45], and six studies [15, 39, 40, 41, 42, 46] did not specify the mean age of participants according to their periodontal status. Four studies involved participants with co‐morbidities [40, 41, 44, 46], and seven studies includedparticipants with a history of urinary tract infection during pregnancy [14, 15, 40, 42, 44, 45, 46]. Smokers were included in eight studies [13, 14, 15, 39, 40, 44, 45, 46], excluded in two studies [41, 42] and one study did not provide information on the smoking status of participants [43].

All included studies defined preterm birth as labour that occurred before 37 completed weeks of gestation, except for one study [44] which defined PTB as labour that occurred between 22 and 34 weeks. All studies used PPD and CAL as clinical parameters to define periodontitis. Four studies [14, 40, 45, 46] defined periodontitis based on the involvement of a minimum of four teeth, three studies [13, 39, 44] considered two teeth and two studies [15, 43] a single tooth. Further, two studies [41, 42] employed variations in periodontitis case definitions, differing in the minimum number of involved teeth. The threshold for PPD ranged from 3 mm [13, 39, 41] to 5 mm [44], and for CAL from 1 mm [13, 39] to 4 mm [41, 42, 43, 44].

### 3.3 Assessment of Methodological Heterogeneity and Quality

Six studies [13, 14, 15, 39, 43, 46] adopted a cohort design, four [40, 42, 44, 45] a case–control design and one [41] a cross‐sectional approach. All studies performed full mouth periodontal examinations. Among them, five studies excluded the third molars [13, 39, 41, 42, 46], while the rest [14, 15, 40, 41, 43, 45] did not specify the inclusion or exclusion of these teeth. Utilising the NOS [22, 23], nine studies [14, 15, 39, 40, 42, 43, 44, 45, 46] had a low risk of bias, while two studies [13, 41] exhibited a high risk of bias. For details, see Online Appendix S2A–C.

### 3.4 Data Analyses

#### 3.4.1 Data Extraction

The data extraction for each included study, detailing the number of participants in each group, with or without a diagnosis of periodontitis, along with the number and percentage of PTB events per group, is presented in Table 3. Two studies [41, 42] provided two periodontitis case definitions. In the study by Macedo et al. [42], the applied definition in the studies by Agueda et al. [46], Lopez et al. [14] and Piscoya et al. [45] was utilised in the overall analysis. In the study by Martínez‐Martínez et al. [41], the definition derived from the criteria outlined in the study by Macedo et al. [42] was adopted in the data analysis. Within the research cohort, 2292 pregnant women were identified with periodontitis, comprising 38% of the total study population. Among the included participants, 1304 women experienced PTB, accounting for 22% of the study cohort.

**TABLE 3:** Prevalence of PTB among periodontitis and non‐periodontitis groups and original outcome of interest of the included studies.

Study ID	1st author year	# Participants periodontitis	# PTB	% PTB	Outcome of interest of the present review presented by the original authors OR or RR (95% CI) *p*‐value
		Non‐periodontitis			
I	Pockpa et al. 2022 [39]	Periodontitis: 201 Non‐periodontitis: 137	50 12	25^a^ 9^a^	Adjusted OR: 3.62 (95% CI 1.80; 7.31) *p* = 0.0003 OR adjusted for confounding variables, not specified.
II	Caneiro et al. 2020 [13]	Periodontitis: 39 Non‐periodontitis: 119	6^b^ 12^b^	15^a^ 10^a^	—
III	Pérez‐Molina et al. 2019 [40]	Periodontitis: 507^a^ Non‐periodontitis: 522^a^	229 114^a^	45^a^ 22^a^	Unadjusted OR: 2.95 (95% CI 2.23; 3.90) *p* < 0.0010 Adjusted OR for multiple pregnancies: 2.25 (95% CI 1.61; 3.14) *p* < 0.001
IV	Martínez‐Martínez et al. 2016 [41]	Periodontitis: 49^a^ Non‐periodontitis: 21^a^	19^a^ 6^a^	39^a^ 29^a^	—
V	Macedo et al. 2014 [42]	Periodontitis: 46^a^ Non‐periodontitis: 250^a^	16^a^ 58^a^	35^a^ 23^a^	Unadjusted OR D1: 1.76 (95% CI 0.90; 3.46) *p* = 0.098 Adjusted OR D1: 1.62 (95% CI 0.80; 3.29) *p* = 0.178 Unadjusted OR D2: 2.15 (95% CI 1.26; 3.67) *p* = 0.004 Adjusted OR D2: 1.98 (95% CI 1.14; 3.43) *p* = 0.015 OR adjusted for: origin, increased appetite, frequency of daily toothbrushing.
VI	Kumar et al. 2013 [43]	Periodontitis: 61 Non‐periodontitis: 132	23 24	38 18	Unadjusted OR: 2.72 (95% CI 1.30; 5.68) *p* < 0.05 Adjusted OR: 1.491 (95% CI 0.709; 3.136) *p* = 0.293 OR adjusted for: age, education, BMI and socioeconomic status.
VII	Martinez de Teyada et al. 2012 [44]	Periodontitis: 125^a^ Non‐periodontitis: 304^a^	34^a^ 50^a^	27^a^ 17^a^	Unadjusted OR: Moderate Periodontitis 0.88 (95% CI 1.41; 4.13) *p* = 0.79 Severe Periodontitis 2.42 *p* = 0.001 Adjusted OR: Moderate Periodontitis 0.85 (95% CI 0.30; 2.40) *p* = 0.75 Severe Periodontitis 2.38 (95% CI 1.36; 2.14) *p* = 0.002 OR adjusted for main confounders, not specified.
VIII	Piscoya et al. 2012 [45]	Periodontitis: 82^a^ Non‐periodontitis: 636^a^	70 290	85^a^ 46^a^	Unadjusted OR: 6.96 (95% CI 3.56; 13.90) *p* = 0.000 Adjusted OR: 6.17 (95% CI 3.18; 11.96) *p* = 0.000 OR adjusted for: socioeconomic and demographic factors. Unadjusted OR: 6.96 (95% CI 3.69; 13.90) *p* = 0.000 Adjusted OR: 7.09 (95% CI 3.69; 13.59) *p* = 0.000 OR adjusted for: maternal age, number of prenatal consultations and smoking habit. Unadjusted OR: 6.96 (95% CI 3.69; 13.90) *p* = 0.000 Adjusted OR: 6.79 (95% CI 3.52; 13.09) *p* = 0.000 OR adjusted for: prenatal clinical intercurrences. Unadjusted OR: 6.96 (95% CI 3.69; 13.09) *p* = 0.000 Adjusted OR: 6.05 (95% CI 3.01; 12.16) *p* = 0.000 OR adjusted for the variables in all groups.
IX	Agueda et al. 2008 [46]	Periodontitis: 338^a^ Non‐periodontitis: 958^a^	31 54^a^	9^a^ 6^a^	Adjusted OR: 1.77 (95% CI 1.08; 2.88) *p* < 0.05 OR adjusted for: age, area of residence, systemic diseases, pregnancy complications, previous PTB, onset prenatal care, type of delivery, untreated caries.
X	López et al. 2002 [14]	Periodontitis: 233 Non‐periodontitis: 406	12 6	5 2	Adjusted RR: 2.9 (95% CI 1.0; 8.1) *p* = 0.045 RR adjusted for: previous PLBW, fewer than six pre‐natal visits and low maternal weight gain.
XI	Offenbacher et al. 2001 [15]	Periodontitis: 611^a^ Non‐periodontitis: 201	150^a^ 38	25^a^ 19	—
	Total	Periodontitis: 2292 Non‐periodontitis: 3686	640 664	28% 18%	
	Total	PTB: 1304 FTB: 4674	640 (49%) periodontitis patients 1652 (35%) periodontitis patients		

Abbreviations: BMI, body mass index; CI, confidence interval; D, definition of periodontitis; FTB, full‐term birth; NA, not available; OR, odds ratio; PLBW, preterm low birth weight, PTB, preterm birth; RR, risk ratio.

^a^ Calculated from the authors of the present review.

^b^ Provided from the original authors.

#### 3.4.2 Descriptive Analysis

The original unadjusted and adjusted OR or risk ratio (RR), along with corresponding 95% CI and *p*‐values, are presented in Table 3. Three studies [13, 15, 41] did not provide this information. Among the studies that provided data, seven [14, 39, 40, 43, 44, 45, 46] reported a significant association between periodontitis and PTB. However, this association lost significance after adjusting for confounding variables in the study by Kumar et al. [43] and in moderate periodontitis patients in the study by Martinez de Teyada et al. [44]. Additionally, the study by Macedo et al. [42] did not demonstrate a significant association when applying periodontitis case definition 1.

All studies except one [14] presented ORs. The unadjusted ORs varied from 0.88 to 6.96, while the adjusted ORs ranged from 0.85 to 7.09. The utilised confounding variables for statistical adjustments across studies were multiple pregnancies [40], origin of participants [42], increased appetite [42], frequency of daily toothbrushing [42], age [15, 43, 45, 46], education [43, 45], BMI [43], socioeconomic status [43, 45], smoking [15, 45], occupation [45], number of prenatal consultations [14, 45], prenatal clinical intercurrences [45], residence area [46], systemic diseases [46], pregnancy complications [15, 46], urinary tract infection [15, 45, 46], chorioamnionitis [15], previous PTB [14, 46], onset of prenatal care [46], type of delivery [46], untreated caries [46] and low maternal weight gain [14], race and marital status of participants [15] and food stamps usage [15].

#### 3.4.3 Meta‐Analysis

**FIGURE 2:**
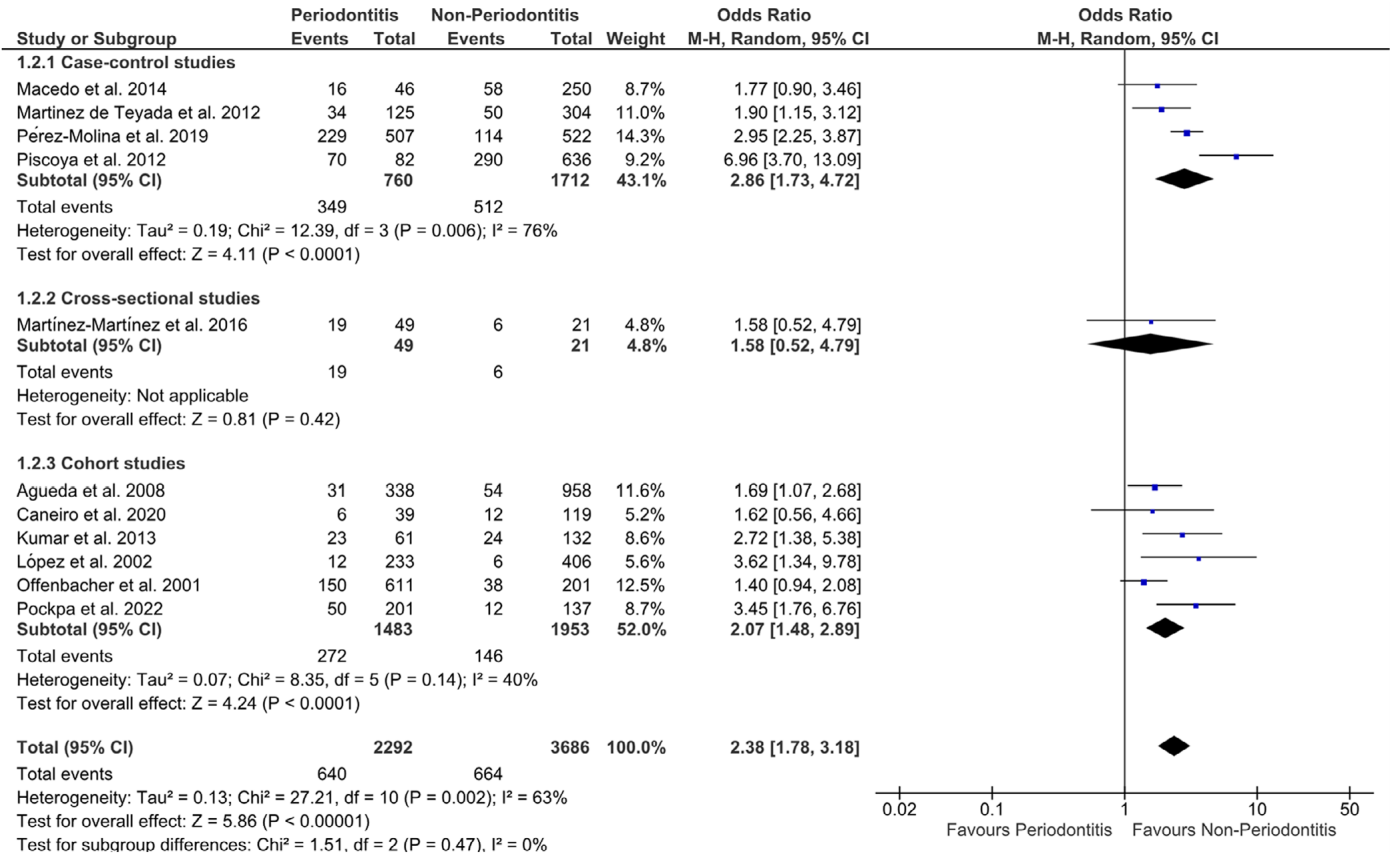
Meta‐analysis of the selected studies. A Chi‐squared test resulting in a *p* < 0.1 was considered an indication of significant statistical heterogeneity. As a rough guide for assessing the possible magnitude of inconsistency across studies, an *I* ^2^ value of 0%–40% was interpreted as non‐imperative, and moderate to considerable heterogeneity was assumed to be present for values above 40%.

Based on 11 included studies using a random‐effects model, pregnant women with periodontitis exhibited a statistically significant increased risk, characterised by a small effect, of experiencing PTB (OR: 2.38 [95% CI: 1.78; 3.18], *p* < 0.00001) (Figure 2). The assessment of heterogeneity was significant and substantial (*I* ^2^ = 63%).

Table 4 summarises the outcomes of the subgroup analyses for the effect size, encompassing the OR, 95% CI, *p*‐values and assessments of statistical heterogeneity. The original forest plots are presented in the Online Appendices S3A–G. Except for two sub‐analyses pertaining to studies with high risk of bias and those excluding smokers, all other sub‐analyses showed a statistically significant heightened risk for pregnant women with periodontitis (OR range: 1.61–4.03) (Table 4). Most of these findings indicated a small effect on the likelihood of PTB, except for the analysis focusing on PTB among women with periodontitis in South America. This particular analysis revealed a statistically significant increased risk with a medium effect (OR: 4.03 [95% CI: 2.71; 6.00], *p* < 0.00001). The assessments of heterogeneity within the subgroup analysis varied from *I* ^2^ = 0% up to *I* ^2^ = 79%, indicating ‘potential not important’ up to ‘substantial heterogeneity’.

**TABLE 4:** Overview subgroup analysis: risk of bias, study design, world continent, health status, history of urinary tract infection, smoking, periodontitis definition, number of examined teeth per participant.

	Included studies	Effect sizes				Heterogeneity		For details see
		OR	Model	95 CI%	*p*	*I* ^2^ value	*p*	Forest plot
Risk of bias								
Low	Agueda et al. 2008 [46] Kumar et al. 2013 [43] Lopez et al. 2012 [14] Macedo et al. 2014 [42] Martinez de Teyada et al. 2012 [44] Offenbacher et al. 2001 [15] Pérez‐Molina et al. 2019 [40] Piscoya et al. 2012 [45] Pockpa et al. 2022 [39]	2.49	Random	[1.82; 3.42]	< 0.00001	69%	0.001	S3‐A
High	Caneiro et al. 2020 [13] Martínez‐ Martínez et al. 2016 [41]	1.60	Fixed	[0.75; 3.44]	0.23	0%	0.98	S3‐A
Study design								
Case–control	Macedo et al. 2014 [42] Martinez de Teyada et al. 2012 [44] Pérez‐Molina et al. 2019 [40] Piscoya et al. 2012 [45]	2.86	Random	[1.73; 4.72]	< 0.0001	76%	0.006	S3‐B
Cohort	Agueda et al. 2008 [46] Caneiro et al. 2020 [13] Kumar et al. 2013 [43] López et al. 2002 [14] Offenbacher et al. 2001 [15] Pockpa et al. 2022 [39]	2.07	Random	[1.48; 2.89]	< 0.0001	40%	0.14	S3‐B
World continent								
Europe	Agueda et al. 2008 [46] Caneiro et al. 2020 [13] Martinez de Teyada et al. 2012 [44]	1.77	Fixed	[1.28; 2.44]	0.0005	0%	0.93	S3‐C
North‐America	Martínez‐Martínez et al. 2016 [41] Offenbacher et al. 2001 [15] Pérez‐Molina et al. 2019 [40]	2.27	Fixed	[1.82; 2.84]	< 0.00001	79%	0.008	S3‐C
South‐America	López et al. 2002 [14] Macedo et al. 2014 [42] Piscoya et al. 2012 [45]	4.03	Fixed	[2.71; 6.00]	< 0.00001	77%	0.01	S3‐C
Country socio‐economic status (17)								
Developed	Agueda et al. 2008 [46] Caneiro et al. 2020 [13] Martinez de Teyada et al. 2012 [44] Offenbacher et al. 2001 [15]	1.61	Random	[1.25; 2.07]	0.0002	0%	0.81	S3‐D
Developing	Kumar et al. 2013 [43] López et al. 2002 [14] Macedo et al. 2014 [42] Martínez‐Martínez et al. 2016 [41] Pérez‐Molina et al. 2019 [40] Piscoya et al. 2012 [45] Pockpa et al. 2022 [39]	3.08	Random	[2.22; 4.26]	< 0.00001	45%	0.09	S3‐D
Health status, smoking, history of urinary tract infections								
Including participants with co‐morbidities	Agueda et al. 2008 [46] Martinez de Teyada et al. 2012 [44] Martínez‐Martínez et al. 2016 [41] Pérez‐Molina et al. 2019 [40]	2.16	Random	[1.54; 3.03]	0.00001	49%	0.12	S3‐E
Excluding participants with co‐morbidities	Caneiro et al. 2020 [13] Kumar et al. 2013 [43] López et al. 2002 [14] Macedo et al. 2014 [42] Offenbacher et al. 2001 [15] Piscoya et al. 2012 [45]	2.53	Random	[1.45; 4.42]	0.001	75%	0.001	S3‐E
Including smokers	Agueda et al. 2008 [46] Caneiro et al. 2020 [13] López et al. 2002 [14] Martinez de Teyada et al. 2012 [44] Offenbacher et al. 2001 [15] Pérez‐Molina et al. 2019 [40] Piscoya et al. 2012 [34] Pockpa et al. 2022 [46]	2.50	Random	[1.74; 3.59]	< 0.00001	73%	0.0006	S3‐E
Excluding smokers	Macedo et al. 2014 [42] Martínez‐Martínez et al. 2016 [41]	1.71	Fixed	[0.96; 3.05]	0.07	0%	0.87	S3‐E
Including participants with history of urinary tract infection	Agueda et al. 2008 [46] López et al. 2002 [14] Macedo et al. 2014 [42] Martinez de Teyada et al. 2012 [44] Offenbacher et al. 2001 [15] Pérez‐Molina et al. 2019 [40] Piscoya et al. 2012 [45]	2.38	Random	[1.63; 3.49]	< 0.00001	76%	0.0004	S3‐E
Periodontitis case definition								
≥ 4 teeth with PPD ≥ 4 mm and CAL ≥ 3 mm at the same site	Agueda et al. 2008 [46] López et al. 2002 [14] Macedo et al. 2014 [42] Pérez‐Molina et al. 2019 [40] Piscoya et al. 2012 [45]	2.86	Random	[1.79; 4.56]	< 0.0001	73%	0.005	S3‐F
≥ 4 mm CAL	Kumar et al. 2013 [43] Martinez de Teyada et al. 2012 [44]	2.15	Fixed	[1.44; 3.21]	0.0002	0%	0.40	S3‐F
≥ 3 mm CAL	Agueda et al. 2008 [46] López et al. 2002 [14] Macedo et al. 2014 [42] Offenbacher et al. 2001 [15] Pérez‐Molina et al. 2019 [40] Piscoya et al. 2012 [45]	2.50	Random	[1.60; 3.90]	< 0.0001	79%	0.0002	S3‐F
≥ 1 mm CAL	Caneiro et al. 2020 [13] Pockpa et al. 2022 [39]	2.87	Fixed	[1.65; 4.99]	0.0002	29%	0.23	S3‐F
PPD > 3 mm	Agueda et al. 2008 [46] Kumar et al. 2013 [43] López et al. 2002 [14] Macedo et al. 2014 [42] Martínez‐Martínez et al. 2016 [41] Offenbacher et al. 2001 [15] Pérez‐Molina et al. 2019 [40] Piscoya et al. 2012 [45]	2.43	Random	[1.68; 3.52]	< 0.00001	72%	0.0009	S3‐F
PPD > 2 mm	Caneiro et al. 2020 [13] Pockpa et al. 2022 [39]	2.87	Fixed	[1.65; 4.99]	0.0002	29%	0.23	S3‐F
≥ 2 affected teeth	Caneiro et al. 2020 [13] Martinez de Teyada et al. 2012 [44] Pockpa et al. 2022 [39]	2.31	Fixed	[1.60; 3.34]	< 0.00001	17%	0.30	S3‐F
≥ 1 affected tooth	Kumar et al. 2013 [43] Offenbacher et al. 2001 [15]	1.63	Fixed	[1.16; 2.31]	0.005	64%	0.10	S3‐F
Number of examined teeth per participant								
Full mouth examination excluding third molars	Agueda et al. 2008 [46] Caneiro et al. 2020 [13] Macedo et al. 2014 [42] Martinez de Teyada et al. 2012 [44] Pockpa et al. 2022 [39]	1.96	Random	[1.50; 2.56]	0.00001	0%	0.50	S3‐G
Full mouth examination without specification on third molars	Kumar et al. 2013 [43] López et al. 2002 [14] Martínez‐Martínez et al. 2016 [41] Offenbacher et al. 2001 [15] Pérez‐Molina et al. 2019 [40] Piscoya et al. 2012 [45]	2.77	Random	[1.72; 4.44]	< 0.0001	76%	0.0009	S3‐G

Meta‐analysis outcome	None	Small	Medium	Large
(OR)	OR 0	OR ≥ 1.68	OR ≥ 3.47	OR ≥ 6.71
As a guideline, by the colour, the effect sizes of the OR were interpreted using a normal standard deviate. Effect sizes of 1.68, 3.47 and 6.71 were considered indicative of small, medium and large magnitudes, respectively [21].				

Test for heterogeneity	Potential not important	Moderate	Substantial	Considerable
(*I* ^2^)	0%–40%	30%–60%	50%–90%	75%–100%
As a guideline, by the colour, to assess the potential magnitude of inconsistency between studies, an *I* ^2^ statistic of 0%–40% may represent unimportant levels of heterogeneity, 30%–60% moderate heterogeneity, 50%—90% substantial heterogeneity and > 75% considerable heterogeneity) [25].				

Abbreviations: CAL, clinical attachment loss; PPD: probing pocket depth.

Sensitivity analyses were performed by evaluating the effect of excluding studies based on specific aspects in the domain of clinical or methodological characteristics. Sensitivity analysis revealed no substantial differences in the OR compared to the overall OR as judged based on overlapping 95% CIs, indicating that the overall analysis was robust.

#### 3.4.4 Publication Bias and TSA Analyses

No evidence of publication bias was detected upon visualisation of the funnel plot, a finding corroborated by the Egger's test (*p* = 0.996) (Online Appendix S4).

**FIGURE 3:**
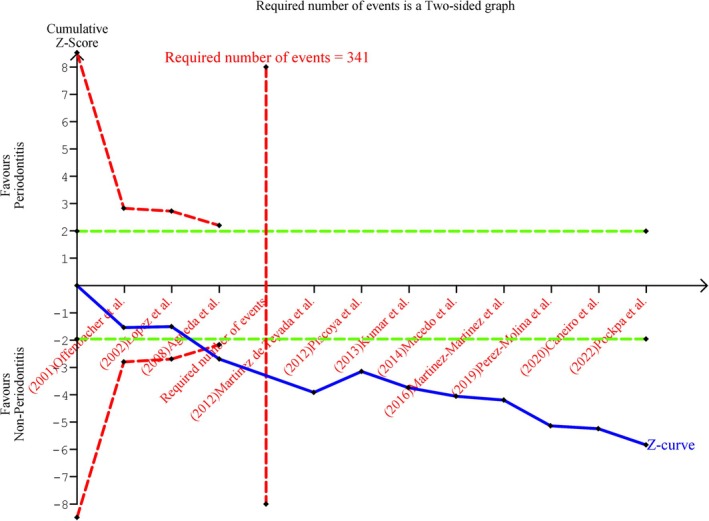
TSA of the selected studies. The cumulative blue *Z*‐curves were constructed with each cumulative *Z*‐value calculated after including a new trial according to publication date. Crossing of the two‐sided *Z* = 1.96 provides a traditionally significant result. Crossing of the red trial sequential monitoring boundaries is needed to obtain reliable evidence adjusted for random error risk. *Z*‐curves not crossing *Z* = 1.96 indicate absence of evidence if the information size is not reached or lack of the predefined intervention effect if the information size is not reached. The green dotted lines represent the traditional boundary. The vertical red line represents the estimated heterogeneity‐adjusted required information size, the number of events for the meta‐analysis sample size.

The overall TSA is shown in Figure 3. The required number of events is reached. The *Z*‐curve does cross the monitoring boundaries; thus, excluding the possibility of type I error. Similarly, the TSA conducted on the low risk of bias studies (Online Appendix S5) reached the required number of events, crossed the monitoring boundaries; thereby, the type I error is ruled out.

#### 3.4.5 Grading the Body of Evidence

Table 5 summarises the various factors used to rate the quality of evidence and strength of recommendations according to the GRADE working group (2014) [37]. There was a moderate level of certainty for a small magnitude of the OR for PTB among women with periodontitis as compared to pregnant women without periodontitis.

**TABLE 5:** GRADE evidence profile for the odds ratio of PTB among periodontitis patients as compared to non‐periodontitis individuals.

Summary of findings table on the body of the estimated evidence profile	
Determinants of quality	Odds ratio 2.38
Study design (Table 2)	Observational studies
# studies (Figure 1)	# 11
# comparisons	# 11
# comparisons in MA (Figures 1 and 2)	# 11
Risk of bias (Appendix S2A–C)	Low to high
Consistency (Tables 3 and 4, Figure 2, Online Appendix S3A–G)	Rather consistent
Directness (Tables 2 and 3, Figure 3, Online Appendix S5)	Rather generalizable
Precision (Table 4, Figures 2 and 3, Online Appendices S3A–G and S5)	Rather precise
Reporting bias (Online Appendix S4)	Possible
Magnitude of the effect (Table 4, Figure 2, Online Appendix S3A–G, Chen et al. 2010 [21])	Small
Strength of the recommendation based on the quality and body of evidence (Figure 3, Online Appendix S5)	Moderate
**Direction of recommendation**	**There is moderate certainty that pregnant women with periodontitis compared to pregnant women without periodontitis have a small risk for PTB.**

## 4 Discussion

The present systematic review assesses, summarises and synthesises the current evidence within dental and medical literature on the risk of PTB in pregnant women with periodontitis. This association may be explained via two potential mechanisms. The first includes a direct pathway, in which oral bacteria and/or their virulence factors enter the systemic circulation through the ulcerated pocket epithelium and reach the foetal‐placental unit. In this condition a transient but frequent bacteremia occurs during daily oral activities, especially toothbrushing or, more extensively, during periodontal therapy [47, 48]. The second includes an indirect pathway, in which inflammatory molecules locally produced within periodontal tissues either reach the foetal‐placental unit via circulation or access the liver and increase the synthesis of cytokines and acute phase proteins which, in turn, could affect the foetal‐placental unit [11]. The MA revealed an OR of 2.38 for pregnant women with periodontitis to have a PTB which according to Chen et al. [28] is interpreted as a weak association between periodontitis and PTB.

### 4.1 Periodontitis and PTB Case Definitions as Eligibility Criteria

The inclusion of studies with high variability in periodontitis case definitions is a common limitation found in previous SR on the topic [12, 17, 18, 49]. The current SR applied a strict inclusion criterion requiring a clear definition of periodontitis. Specifically, this definition was based solely on full‐mouth clinical examinations, assessing the amount of PPD in combination with CAL and/or radiographic alveolar bone loss, aligning with key aspects outlined in the recent World Workshop on the classification of periodontal diseases [50, 51]. Partial mouth examinations or assessments based solely on radiographic bone loss are susceptible to underestimating or misclassifying periodontitis and may not adequately reflect its full clinical extent [50, 52, 53].

A crucial consideration in conducting a full mouth assessment involves deciding whether to include or exclude third molars. A previous study [54] showed that clinical periodontal measures could be accurately estimated on visible third molars of pregnant women. However, consensus within the dental scientific literature regarding the approach to this matter remains elusive. Notably, in the included studies that third molars were specifically excluded from the full mouth examination, the estimated OR was numerically lower (OR: 1.96 [95% CI: 1.50; 2.56]) compared to those where no specific directive was provided regarding the third molars (OR: 2.77, 95% CI [1.72; 4.44]) (Table 4). The latter finding might be ascribed to the greater severity of periodontitis in pregnant women that third molars have been included in the periodontal clinical examination [55]. The new periodontal classification does not address this issue, even though periodontal defects caused by impacted or malpositioned third molars should not be classified as periodontitis cases [50, 51].

Diagnosis based exclusively on radiographic evidence of bone loss may underestimate periodontitis prevalence, particularly in mild cases [53]. Conversely, diagnosis based only on CAL may misclassify individuals with a reduced but healthy periodontium as periodontitis patients [51, 56]. Furthermore, studies that only measured PPDs were excluded due to the risk of overestimating periodontitis prevalence, as pseudo pockets may inflate diagnosis rates [51, 56]. Notably, a recent SR and MA showed substantial underestimation of bone levels by both clinical and radiographic measurements compared to intraoperative level measurements [57].

Another critical criterion was the method of defining PTB based on gestational age estimation. Numerous studies relied on participant self‐report of the last menstrual period to estimate gestational age, a subjective method prone to random error and overestimation due to factors like delayed ovulation [58, 59]. To address this limitation, the present SR exclusively included studies where gestational age assessment was confirmed by a health care professional following an objective clinical examination.

### 4.2 OR, RR, TSA and Type I Error

The OR and the RR are the two primary measures of associations in epidemiology [60]. Typically, case–control studies employ the OR, while cohort studies use the RR [61]. In instances where the occurrence of an outcome within a cohort study is prevalent (e.g., exceeding 10% within the unexposed group), the use of the OR tends to lead to an overestimation of the risk ratio [60, 61]. However, under the premise of the ‘rare disease assumption’, the OR may serve as an acceptable approximation of the RR [60, 61]. The present SR included six cohort studies [13, 14, 15, 39, 43, 46]. Among these, only one [14] used the RR. Two studies [39, 46] appropriately presented an OR since the incidence in the non‐periodontitis group was < 10%. In contrast, one study [43] incorrectly used the OR due to an observed incidence of PTB > 10% within the non‐periodontitis group. Lastly, two studies [13, 15] did not report data for either the OR or the RR.

The current statistical analysis is supported by the results derived from TSA. Both the overall TSA and the TSA performed on studies with low risk of bias ruled out the possibility of type I error. Consequently, the two studies [13, 41] with a high risk of bias do not contribute to the potential type I error. Although the overall OR being 2.38 [95% CI: 1.78; 3.18] and the sub‐analysis on low risk of bias studies indicating a numerically higher risk (OR: 2.49 [95% CI: 1.82; 3.42]), there is firm overlap in the 95% CIs. Conversely, the sub‐analysis for high risk of bias studies exhibited the lowest OR (1.60), which was not statistically significant (95% CI [0.75; 3.44]). This challenges the capability of the risk of bias assessment tool to differentiate between high risk studies with large effects and low risk studies with small effects. This was not supported by the current series of MA nor the findings of TSA's.

### 4.3 Country Differences

Social and economic disparities have been reported as risk factors for both periodontitis [62] and PTB [63, 64], suggesting a commonality in risk. This observation is further supported by the subgroup analysis carried out in our study (Table 4, Online Appendix S3D). Most of the included investigations, as per the United Nations classification [24], were conducted in developing countries [14, 39, 40, 41, 42, 43, 45]. The estimated OR (3.08) from these countries revealed a significant, albeit weak [28], association between periodontitis and PTB. Instead, the OR (1.61) of the studies from developed countries did not signify an association between the two conditions [28].

The sub‐analysis on world continents assessed the data from different geographical regions regardless of the social and economic status of the countries involved. However, the results may not be fully representative of specific world continents due to the limited inclusion of studies. Specifically, only three studies from Europe [13, 44, 46], three from North America [15, 40, 41], two located in Mexico [40, 41], three from South America [14, 42, 45], with two of them conducted in Brazil [42, 45] and one from Africa [39] were included in the sub‐analysis. Also, no studies met the inclusion criteria of the present SR from Oceania, Central America, West and East Asia. The OR for South America was calculated as 4.03, indicating a medium association between periodontitis and PTB [28]. This finding might be attributed to the high prevalence of caesarean sections in Brazil, which has been linked to an increased risk of future PTB [65, 66, 67]. Thus, social and cultural aspects can serve as a risk factors.

### 4.4 Medical Co‐Factors

Smoking is a shared risk factor for periodontitis and PTB [5, 68]. Eight studies included smokers in their sample [13, 14, 15, 39, 40, 44, 45, 46], while two excluded them [41, 42]. Sub‐analysis of these studies reaffirmed the role of smoking as a risk factor for both conditions. The OR was higher in the studies that included smokers compared to the overall analysis. On the contrary, when smokers were excluded, the OR was lower than the overall analysis (Table 4). As a supplementary analysis, it would be valuable to exclusively examine smokers. This may confirm that smoking elevates the risk of PTB in pregnant women with periodontitis, potentially demonstrating a larger effect size.

Intrauterine infections are estimated to contribute to 25%–40% of all PTBs [4]. Microorganisms responsible for these infections can activate the innate immune system, leading to the release of chemokines and cytokines within the uterine environment [4]. This mechanism parallels the pathway underlying the association between periodontitis and PTB. Only seven studies mentioned the inclusion of participants with a history of urinary tract infection during pregnancy [14, 15, 40, 42, 44, 45, 46], with three of them adjusting the ORs for this factor [15, 45, 46]. Sub‐analysis showed an equal OR of 2.38 compared to the overall analysis, although with higher heterogeneity of 76%, confirming the detrimental role of intrauterine infections in the development of PTB.

Numerous medical co‐morbidities could be linked to PTB [69]. Six studies explicitly mentioned the exclusion of participants with medical co‐morbidities [13, 14, 15, 42, 43, 45], whilst four included participants with such conditions [40, 41, 44, 46]. The sub‐analysis of studies that excluded participants with medical co‐morbidities revealed a higher OR (Table 4). However, this finding should be interpreted cautiously, given that three of these studies were conducted in developing countries [14, 42, 43, 45].

### 4.5 Results in Relation to Previous Evidence

Multiple SRs have explored the association between periodontitis and PTB. The scientific literature contains at least eight such reviews [12, 16, 17, 49, 70, 71, 72, 73], spanning from 2005 [70] to 2022 [16]. Also, at least two umbrella reviews have synthesised the findings on this topic [74, 75]. Variations among these SRs exist, potentially influencing their outcomes, particularly concerning the definitions of periodontitis and PTB as eligibility criteria. For instance, Chambrone et al. [17] and Corbella et al. [71] reported respective RRs of 1.70 and ORs of 1.78, employing different inclusion criteria with respect to periodontitis and PTB definitions, compared to the present study.

The SR prepared for the European Federation of Periodontology (EFP)/American Academy of Periodontology (AAP) workshop in 2013 [12] incorporated 11 case–control and three prospective studies, where periodontitis and PTB were diagnosed by health care professionals. However, the participants' periodontal status was assessed either with full mouth or partial mouth examination protocols. A pooled OR of 2.47 for case–control studies and a pooled RR of 1.15 for prospective studies were calculated. In contrast, the present study computed only ORs irrespective of the original design, resulting in ORs 2.86 for case–control and 2.07 for cohort, both indicating a small effect size [28]. The reported OR of 2.47 in the EFP/AAP paper [12] was similarly interpreted as indicating a small effect size [28], while the RR 1.15 (95% CI [0.89; 1.49]) was not statistically significant.

In 2016, Corbella et al. [72] performed a new MA and found a slightly lower RR of 1.61 (95% CI: 1.33–1.95) compared to their 2012 MA [71]. Comparable magnitude of RR 1.67 (95% CI: 1.67–2.38) was observed from Moliner‐Sánchez et al. in 2020 [73]. Despite both studies showing significant associations, Olivier et al. [76] classified these effects as small. The first SR from Khader et al. [70] estimated the highest OR of 3.87, though its robustness was limited due to its reliance on only two studies. Recently, Manrique‐Corredor et al. [49] and Zhang et al. [16] assessed ORs of 2.01 and 1.57, respectively, but with less specific inclusion criteria concerning periodontitis and PTB definitions.

### 4.6 Strengths, Limitations and Recommendations

- The present SR employed a pre‐registered and reproducible protocol, characterised by strict inclusion criteria related to the definitions of periodontitis and PTB cases.
- The statistical analysis was reinforced by the findings of TSA and incorporated the assessment of methodical quality of the included studies.
- Maternal age was recognised as a risk factor for PTB [4, 5], with both younger and older women showing higher susceptibility. Additionally, the prevalence and severity of periodontitis rise with age [77]. Most of the included studies in the MA either do not report mean ages based on the periodontal status of the participants [15, 39, 40, 41, 42, 46] or do not provide any age information at all [44, 45]. It would be of interest to explore the raw data of the included studies regarding age in a secondary analysis.
- Various confounding variables, encompassing personal factors (education, socioeconomic and marital status, occupation, food stamps usage, residence area, origin, race of participants), dental factors (frequency of daily toothbrushing, untreated caries), medical factors (BMI, smoking, increased appetite, systemic diseases, urinary tract infection) and pregnancy‐related factors (multiple pregnancies, number of prenatal consultations, prenatal clinical intercurrences, pregnancy complications, chorioamnionitis, previous PTB, onset of prenatal care, type of delivery and low maternal weight gain), were utilised for statistical adjustments across the original studies included in this SR. However, the present MA did not incorporate adjustments for these confounders. Nonetheless, the conducted sensitivity and subgroup analyses support the finding of the present MA (Table 4).
- Future observational studies investigating the risk of PTB in pregnant women with periodontitis should adhere to a standardised definition of periodontitis and the dual presentation of disease status as both dichotomous and continuous variables, aligning with the latest guidelines from the World Workshop on the Classification of Periodontal and Peri‐Implant Diseases and Conditions [50, 51]. The validation of PTB events should be based on objective clinical examinations performed by health care professionals. Furthermore, adjusting for potential confounders is essential to avoid bias and ensure the accuracy of the results. Longitudinal study designs are preferable as they offer a more robust assessment of the association between periodontitis and PTB [78, 79].

## 5 Conclusion

There is moderate certainty that pregnant women with periodontitis compared to pregnant women without periodontitis have a small risk for PTB. Health care professionals should be vigilant of the potential risk for PTB in pregnant women with periodontitis. Public health care initiatives should emphasise the prevention of periodontitis as a constituent of health promotion efforts including the focus on personal, medical and environmental factors.

## 6 Clinical Relevance

### 6.1 Scientific Rationale for the Study

Previous systematic reviews have established links between periodontitis and PTB. However, there has been inconsistency in how both conditions are defined across the studies. The individual studies have produced conflicting results due to differences in their methodologies, clinical approaches and quality standards. Therefore, a thorough assessment based on rigorous and clinically relevant criteria is crucial.

### 6.2 Principal Findings

Confounding variables lower the risk. There is moderate certainty that pregnant women with periodontitis compared to those without have a small risk for PTB. Sub‐analyses and TSA support these findings.

### 6.3 Practical Implications

Healthcare providers need to remain alert to the potential threat of PTB in pregnant women with periodontitis. Public health initiatives should prioritise preventing periodontitis as part of broader efforts to promote health, addressing factors related to individuals, medical care and the environment.

## Author Contributions

**Dimitris Sokos:** contributed to design, search and selection, analysis and interpretation, and drafted the manuscript. **Dagmar Else Slot:** contributed to design, analysis and interpretation, and critically revised the manuscript. **Berna Dogan:** contributed to design, search and selection, analysis and interpretation, and drafted the manuscript. **Sergio Bizzarro:** contributed to conception and design, selection, analysis and interpretation, and critically revised the manuscript. All authors gave final approval and agreed to be accountable for all aspects of work ensuring integrity and accuracy.

## Ethics Statement

The ACTA ethical approval committee agreed in proceeding and the study is registered by 2021‐50260.

## Conflicts of Interest

The authors declare no conflicts of interest.

## Supporting information


**Appendix S1:** idh70001‐sup‐0001‐Appendix.pdf.

## Data Availability

Data were derived from resources available in original papers that are published in the public domain.
