# Supplementary material for: The Risk of Preterm Birth in Women With Periodontitis: A Systematic Review and Meta‐Analysis
Source: Int J Dent Hyg. 2025 Oct 2;24(1):116–35. doi: 10.1111/idh.70001 (PMC12748043; doi:10.1111/idh.70001)
Supplement: Supplementary file 1 — Appendix S1: idh70001‐sup‐0001‐Appendix.pdf. [file IDH-24-116-s001.pdf]

ONLINE APPENDICES

The risk of preterm birth in women with periodontitis: A systematic review and meta-analysis

Dimitris Sokos (ORCID: 0000-0002-5609-0864)
Dagmar Else Slot (ORCID: 0000-0001-7234-0037)
Berna Dogan
Sergio Bizzarro (ORCID: 0000-0002-5228-5998)

Online Appendix S1

Excluded studies (N = 109) based on the full text reading of the articles.

Lack of periodontitis definition based on study's inclusion criteria	1. Abati et al. 2013<br>2. Ali & Abidin 2012<br>3. Alves et al. 2006<br>4. Bošnjak et al. 2006<br>5. Choi et al. 2021<br>6. Choudhury et al. 2022<br>7. Costa et al. 2019<br>8. Davenport et al. 1998<br>9. Davenport et al. 2002<br>10. Farrell et al. 2006<br>11. Fogacci et al. 2018<br>12. Gesase et al. 2018<br>13. Gilani et al. 2024<br>14. Goepfert et al. 2004<br>15. Harper et al. 2011<br>16. Hasegawa et al. 2003<br>17. Herrera-Serna et al. 2024<br>18. Hirano et al. 2010<br>19. Holbrook et al. 2004<br>20. Jarjoura et al. 2005<br>21. Jeffcoat et al. 2001<br>22. Khader et al. 2009<br>23. Kopycka-Kedzierawski et al. 2019<br>24. Krüger et al. 2019<br>25. Lee et al. 2021<br>26. Lee et al. 2022<br>27. Mahilkar et al. 2021<br>28. Manau et al. 2008<br>29. Mokeem et al. 2004<br>30. Moore et al. 2004<br>31. Moore et al. 2005<br>32. Nabet et al. 2010<br>33. Noack et al. 2005<br>34. Offenbacher et al. 1996<br>35. Offenbacher et al. 2006<br>36. Patel et al. 2024<br>37. Perunovic et al. 2016<br>38. Pitiphat et al. 2008

	39. Pratiksha et al. 2016<br>40. Rakoto-Alson et al. 2010<br>41. Reza Karimi et al. 2016<br>42. Ryu et al. 2010<br>43. Sánchez et al. 2007<br>44. Santa Cruz et al. 2013<br>45. Santos-Pereira et al. 2007<br>46. Savintha et al. 2022<br>47. Sharma et al. 2009<br>48. Skuldbøl et al. 2006<br>49. Soucy-Giguére et al. 2016<br>50. Srinivas et al. 2009<br>51. Tellapragada et al. 2016<br>52. Thomas et al. 2023<br>53. Trivedi et al. 2018<br>54. Turton & Africa 2017<br>55. Wood et al. 2006<br>56. Ye et al. 2020
Lack of PTB definition based on study's inclusion criteria	1. Al-Rawi et al. 2022<br>2. Alchalabi et al. 2013<br>3. Arteaga-Guerra et al. 2010<br>4. Bartha et al. 2022<br>5. Basha et al. 2015<br>6. Baskaradoss et al. 2011<br>7. Bassani et al. 2007<br>8. Bhavsar et al. 2023<br>9. Bulut et al. 2014<br>10. Choi et al. 2021<br>11. Damante et al. 2022<br>12. Davenport et al. 1998<br>13. Davenport et al. 2002<br>14. de Oliveira et al. 2021<br>15. de Vasconcelos Calixto et al. 2024<br>16. Dörtbudak et al. 2005<br>17. Farrell et al. 2006<br>18. Gallagher-Cobos et al. 2022<br>19. Gazolla et al. 2007<br>20. Giannella et al. 2011<br>21. Gilani et al. 2024<br>22. Goepfert et al. 2004

	23. Gomes-Filho et al. 2007<br>24. Govindaraju et al. 2015<br>25. Guimarães et al. 2010<br>26. Herrera-Serna et al. 2024<br>27. Hirano et al. 2010<br>28. Jeffcoat et al. 2001<br>29. Khader et al. 2009<br>30. Krüger et al. 2019<br>31. Lauren et al. 2012<br>32. Le et al. 2007<br>33. Lee et al. 2021<br>34. Lee et al. 2022<br>35. Lima et al. 2023<br>36. Lohsoonthorn et al. 2009<br>37. Mahilkar et al. 2021<br>38. Marakoglou et al. 2008<br>39. Marin et al. 2005<br>40. Márquez-Corona et al. 2021<br>41. Massaro et al. 2020<br>42. Meqa et al. 2017<br>43. Mesa et al. 2013<br>44. Micu et al. 2020<br>45. Mokeem et al. 2004<br>46. Moncunill-Mira et al. 2021<br>47. Moliterno et al. 2005<br>48. Moore et al. 2004<br>49. Moore et al. 2005<br>50. Offenbacher et al. 1996<br>51. Patel et al. 2024<br>52. Pratiksha et al. 2016<br>53. Rakoto-Alson et al. 2010<br>54. Reza Karimi et al. 2016<br>55. Savintha et al. 2022<br>56. Sert et al. 2011<br>57. Siqueira et al. 2007<br>58. Skuldbøl et al. 2006<br>59. Soucy-Giguére et al. 2016<br>60. Thomas et al. 2023<br>61. Trivedi et al. 2018<br>62. Usin et al. 2016

	63. Uwambaye et al. 2021<br>64. Vettore et al. 2008<br>65. Vogt et al. 2010<br>66. Wang et al. 2013<br>67. Wood et al. 2006<br>68. Yanaranci et al. 2024
No PTB participants group	1. Blanc et al. 2015<br>2. Calabrese et al. 2010<br>3. Calixto et al. 2019<br>4. Fogacci et al. 2018<br>5. Noack et al. 2005<br>6. Pozo et al. 2016<br>7. Ye et al. 2020
Lack of non-periodontitis group	1. Al-Rawi et al. 2022<br>2. Alan et al. 2025<br>3. Hasegawa et al. 2003<br>4. Massaro et al. 2020
Lack of periodontitis group	1. Alan et al. 2025
Different aim of study	1. Iwanaga et al. 2011<br>2. Lee et al. 2016<br>3. López 2008<br>4. Moore et al. 2004<br>5. Radochova et al. 2019<br>6. Riché et al. 2002<br>7. Sugita et al. 2012
Different study design	1. Tang & Chen 2024
Same cohort with other included study	1. Manau et al. 2008

PTB: preterm birth

References:

1. Abati S, Villa A, Cetin I, et al. Lack of association between maternal periodontal status and adverse pregnancy outcomes: a multicentric epidemiologic study. The Journal of Maternal-Fetal & Neonatal Medicine. 2013; 26(4): 369-372.
2. Al-Rawi NH, Imran NK, Abdulkareem AA, et al. Association between maternal periodontitis, acute-phase reactants and preterm birth. Oral Dis. 2022; 28(7): 1995-1999.
3. Alan M, Sorsa T, Meriç Kantar P, et al. Active-Matrix Metalloproteinase-8, Myeloperoxidase in Relation With Periodontics, Preterm Birth. Oral Dis. 2025;31(4):1333-1341.

4. Alchalabi HA, Al Habashneh R, Jabali OA, Khader YS. Association between periodontal disease and adverse pregnancy outcomes in a cohort of pregnant women in Jordan. Clin Exp Obstet Gynecol. 2013;40(3):399-402.
5. Ali TB & Abidin KZ. Relationship of periodontal disease to pre-term low birth weight infants in a selected population--a prospective study. Community Dent Health. 2012; 29(1): 100-5.
6. Alves RT, Ribeiro RA. Relationship between maternal periodontal disease and birth of preterm low weight babies. Braz Oral Res. 2006; 20(4): 318-23.
7. Arteaga-Guerra JJ, Cerón-Souza V, Mafla AC. Dynamic among periodontal disease, stress, and adverse pregnancy outcomes. Rev Salud Publica (Bogota). 2010; 12(2): 276-286.
8. Bartha V, Steinmacher S, Wittlinger R, et al. Gain a Baby Lose a Tooth-Is There an Association between Periodontitis and Preterm Birth? J Clin Med. 2022; 11(23): 7183.
9. Basha S, Shivalinga Swamy H, et al. Maternal Periodontitis as a Possible Risk Factor for Preterm Birth and Low Birth Weight--A Prospective Study. Oral Health Prev Dent. 2015; 13(6): 537-44.
10. Baskaradoss JK, Geevarghese A, Kutty VR. Maternal periodontal status and preterm delivery: a hospital based case-control study. J Periodontal Res. 2011; 46(5): 542-9.
11. Bassani DG, Olinto MT, Kreiger N. Periodontal disease and perinatal outcomes: a case-control study. J Clin Periodontol. 2007; 34(1): 31-9.
12. Bhavsar NV, Trivedi S, Vachhani KS, et al. Association between preterm birth and low birth weight and maternal chronic periodontitis: A hospital-based case-control study. Dent Med Probl. 2023; 60(2): 207-217.
13. Blanc V, O'Valle F, Pozo E, et al. Oral bacteria in placental tissues: increased molecular detection in pregnant periodontitis patients. Oral Dis. 2015; 21(7): 905-12.
14. Bošnjak A, Relja T, Vučićević-Boras V, et al. Pre-term delivery and periodontal disease: a case-control study from Croatia. J Clin Periodontal 2006; 33: 710–716.
15. Bulut G, Olukman O, Calkavur S. Is there a relationship between maternal periodontitis and pre-term birth? A prospective hospital-based case-control study. Acta Odontol Scand. 2014; 72(8): 866-73.
16. Calabrese N, Calabrese A, Nibali L, et al. Is there any association between periodontitis and preterm low birth weight? J Matern Fetal Neonatal Med. 2010; 23(11): 1288-93.
17. Calixto NR, Alves CM, Abreu LM, et al. Detection of periodontal pathogens in mothers of preterm birth and/or low weight. Med Oral Patol Oral Cir Bucal. 2019; 24(6): e776-e781.
18. Choi SE, Choudhary A, Ahern JM, et al. Association between maternal periodontal disease and adverse pregnancy outcomes: an analysis of claims data. Fam Pract. 2021; 38(6) :718-723.

19. Choudhury P, Rao R, Prabhu S, et al. Microorganisms of maternal periodontitis cause adverse pregnancy outcomes in gestational diabetic individuals: a preliminary observational report. Quintessence Int. 2022; 53(10): 850-857.
20. Costa EM, de Araujo Figueiredo CS, et al. Periodontopathogenic microbiota, infectious mechanisms and preterm birth: analysis with structural equations (cohort-BRISA). Arch Gynecol Obstet. 2019; 300(6): 1521-1530.
21. Damante CA, Foratori GA Junior, de Oliveira Cunha P, et al. Association among gestational diabetes mellitus, periodontitis and prematurity: a cross-sectional study. Arch Endocrinol Metab. 2022; 66(1): 58-67.
22. Davenport ES, Williams CE, Sterne JA, et al. The East London Study of Maternal Chronic Periodontal Disease and Preterm Low Birth Weight Infants: study design and prevalence data. Ann Periodontol. 1998; 3(1): 213-21.
23. Davenport ES, Williams CE, Sterne JA, et al. Maternal periodontal disease and preterm low birthweight: case-control study. J Dent Res. 2002; 81(5): 313-8.
24. de Oliveira LJC, Cademartori MG, Schuch HS, et al. Periodontal disease and preterm birth: Findings from the 2015 Pelotas birth cohort study. Oral Dis. 2021; 27(6): 1519-1527.
25. de Vasconcelos Calixto NR, Lopes FF, Franco MMP, Gomes-Filho IS, Benatti BB, Alves CMC. Comparative Study on the Periodontal Parameters Used in Diagnosing Periodontitis in Puerperae and Periodontitis' Relationship with the Birth of Preterm Infants: A Case-Control Study. Int J Environ Res Public Health. 2024;21(2):156.
26. Dörtbudak O, Eberhardt R, Ulm M, et al. Periodontitis, a marker of risk in pregnancy for preterm birth. J Clin Periodontol. 2005; 32(1): 45-52.
27. Farrell S, Ide M, Wilson RF. The relationship between maternal periodontitis, adverse pregnancy outcome and miscarriage in never smokers. J Clin Periodontol. 2006; 33(2):115-20.
28. Fogacci MF, Cardoso EOC, Barbirato DDS, et al. No association between periodontitis and preterm low birth weight: a case-control study. Arch Gynecol Obstet. 2018; 297(1): 71-76.
29. Gallagher-Cobos G, Almerich-Torres T, Montiel-Company JM, et al. Relationship between Periodontal Condition of the Pregnant Woman with Preterm Birth and Low Birth Weight. J Clin Med. 2022; 11(22): 6857.
30. Gazolla CM, Ribeiro A, Moysés MR, et al. Evaluation of the incidence of preterm low birth weight in patients undergoing periodontal therapy. J Periodontol. 2007; 78(5): 842-8.
31. Gesase N, Miranda-Rius J, Brunet-Llobet L, et al. The association between periodontal disease and adverse pregnancy outcomes in Northern Tanzania: a cross-sectional study. Afr Health Sci. 2018; 18(3): 601-611.

32. Giannella L, Giulini S, Cerami LB, et al. Periodontal disease and nitric oxide levels in low risk women with preterm labor. Eur J Obstet Gynecol Reprod Biol. 2011; 158(1): 47-51.
33. Gilani SI, Niaz A, Afridi S. Maternal periodontitis as a risk factor for preterm birth: A cross-sectional study. J Dent Res Dent Clin Dent Prospects. 2024;18(1):72-76.
34. Goepfert AR, Jeffcoat MK, Andrews WW, et al. Periodontal disease and upper genital tract inflammation in early spontaneous preterm birth. Obstet Gynecol. 2004; 104(4): 777-783.
35. Gomes-Filho IS, Cruz SS, Rezende EJ, et al. Exposure measurement in the association between periodontal disease and prematurity/low birth weight. J Clin Periodontol. 2007; 34(11): 957-63.
36. Govindaraju P, Venugopal S, Shivakumar MA, et al. Maternal periodontal disease and preterm birth: A case-control study. J Indian Soc Periodontol. 2015; 19(5): 512-5.
37. Guimarães AN, Silva-Mato A, Miranda Cota LO, et al. Maternal periodontal disease and preterm or extreme preterm birth: an ordinal logistic regression analysis. J Periodontol. 2010; 81(3): 350-8.
38. Harper LM, Parry S, Stamilio DM, et al. The interaction effect of bacterial vaginosis and periodontal disease on the risk of preterm delivery. Am J Perinatol. 2012; 29(5):347-52.
39. Hasegawa K, Furuichi Y, Shimotsu A, et al. Associations between systemic status, periodontal status, serum cytokine levels, and delivery outcomes in pregnant women with a diagnosis of threatened premature labor. J Periodontol. 2003; 74(12): 1764-70.
40. Herrera-Serna BY, López-Soto OP, Rendón-Blandón DL, Alfonso-Galeano E, Salgado-Yepes LV, Chacón T. Association of birth and periodontal disease in Bolivia, Chile and Colombia. Biomedica. 2024;44(3):355-367.
41. Hirano E, Sugita N, Kikuchi A, et al. Peroxisome proliferator-activated receptor gamma polymorphism and periodontitis in pregnant Japanese women. J Periodontol. 2010; 81(6): 897-906.
42. Holbrook WP, Oskarsdóttir A, Fridjónsson T, et al. No link between low-grade periodontal disease and preterm birth: a pilot study in a healthy Caucasian population. Acta Odontol Scand. 2004; 62(3): 177-9.
43. Jarjoura K, Devine PC, Perez-Delboy A, et al. Markers of periodontal infection and preterm birth. Am J Obstet Gynecol. 2005; 192(2): 513-9.
44. Jeffcoat MK, Geurs NC, Reddy MS, et al. Periodontal infection and preterm birth: results of a prospective study. J Am Dent Assoc. 2001; 132(7): 875-880.
45. Iwanaga R, Sugita N, Hirano E, et al. FcγRIIB polymorphisms, periodontitis and preterm birth in Japanese pregnant women. J Periodontal Res. 2011; 46(3): 292-302.
46. Khader Y, Al-shishani L, Obeidat B, et al. A. Maternal periodontal status and preterm low birth weight delivery: a case-control study. Arch Gynecol Obstet. 2009; 279(2): 165-9.

47. Kopycka-Kedzierawski DT, Li D, Xiao J, et al. Association of periodontal disease with depression and adverse birth outcomes: Results from the Perinatal database; Finger Lakes region, New York State. PLoS One. 2019; 14(4): e0215440.
48. Krüger MSDM, Casarin RP, Pinto GDS, et al. Maternal periodontal disease and adverse perinatal outcomes: is there an association? A hospital-based case-control study. J Matern Fetal Neonatal Med. 2019; 32(20): 3401-3407.
49. Lauren M, Minire A, Maldi X, et al. The impact of periodontitis in the preterm birth and body size of newborns. Mater Sociomed. 2012; 24(1): 44-7.
50. Le HT, Jareinpituk S, Kaewkungwal J, Pitiphat W. Increased risk of preterm birth among non- smoking, non- alcohol drinking women with maternal periodontitis. Southeast Asian J Trop Med Public Health. 2007 May;38(3):586-93.
51. Lee HJ, Ha JE, Bae KH. Synergistic effect of maternal obesity and periodontitis on preterm birth in women with pre-eclampsia: a prospective study. J Clin Periodontol. 2016; 43(8): 646-51.
52. Lee KS, Kim ES, Kim DY, et al. Association of Gastroesophageal Reflux Disease with Preterm Birth: Machine Learning Analysis. J Korean Med Sci. 2021; 36(43): e282.
53. Lee YL, Hu HY, Chou SY, et al. Periodontal disease and preterm delivery: a nationwide population-based cohort study of Taiwan. Sci Rep. 2022; 12(1): 3297.
54. Lima KM, Alves CM, Vidal FC, et al. Fusobacterium nucleatum and Prevotella in women with periodontitis and preterm birth. Med Oral Patol Oral Cir Bucal. 2023; 28(5): e450-e456.
55. Lohsoonthorn V, Kungsadalpipob K, Chanchareonsook P, et al. Is maternal periodontal disease a risk factor for preterm delivery? Am J Epidemiol. 2009; 169(6): 731-9.
56. López R. Periodontal disease and adverse pregnancy outcomes. Evid Based Dent. 2008; 9(2): 48.
57. Mahilkar S, Malagi SK, Soni A, et al. IL-17, A Possible Salivary Biomarker for Preterm Birth in Females with Periodontitis. J Obstet Gynaecol India. 2021; 71(3): 262-267.
58. Manau C, Echeverria A, Agueda A, et al. Periodontal disease definition may determine the association between periodontitis and pregnancy outcomes. J Clin Periodontol. 2008; 35(5): 385-97.
59. Marakoglu I, Gursoy UK, Marakoglu K, et al. Periodontitis as a risk factor for preterm low birth weight. Yonsei Med J. 2008; 49(2): 200-3.
60. Marin C, Segura-Egea JJ, Martínez-Sahuquillo A, Bullón P. Correlation between infant birth weight and mother's periodontal status. J Clin Periodontol. 2005; 32(3): 299-304.
61. Márquez-Corona ML, Tellez-Girón-Valdez A, Pontigo-Loyola AP, et al. Preterm birth associated with periodontal and dental indicators: a pilot case-control study in a developing country. J Matern Fetal Neonatal Med. 2021; 34(5): 690-695.

62. Massaro CR, Buratti M, de Paula TNP, Piana EA, Wachter F, Hoshi AT, Nassar CA, Nassar PO. Maternal periodontal disease as a risk factor for preterm birth and low-birth-weight babies: a case-control study. Gen Dent. 2020; 68(6): 44-49.
63. Meqa K, Dragidella F, Disha M, Sllamniku-Dalipi Z. The Association between Periodontal Disease and Preterm Low Birthweight in Kosovo. Acta Stomatol Croat. 2017; 51(1): 33-40.
64. Mesa F, Pozo E, Blanc V, et al. Are periodontal bacterial profiles and placental inflammatory infiltrate in pregnancy related to birth outcomes? J Periodontol. 2013; 84(9): 1327-36.
65. Micu IC, Roman A, Ticala F, et al. Relationship between preterm birth and post-partum periodontal maternal status: a hospital-based Romanian study. Arch Gynecol Obstet. 2020; 301(5): 1189-1198.
66. Mokeem SA, Molla GN, Al-Jewair TS. The prevalence and relationship between periodontal disease and pre-term low birth weight infants at King Khalid University Hospital in Riyadh, Saudi Arabia. J Contemp Dent Pract. 2004; 5(2):40-56.
67. Moliterno LF, Monteiro B, Figueredo CM, et al. Association between periodontitis and low birth weight: a case-control study. J Clin Periodontol. 2005; 32(8): 886-90.
68. Moncunill-Mira J, Brunet-Llobet L, Cuadras D, et al. Do the clinical criteria used to diagnose periodontitis affect the association with prematurity? Odontology. 2021; 109(2): 455-463.
69. Moore S, Ide M, Coward PY, et al. A prospective study to investigate the relationship between periodontal disease and adverse pregnancy outcome. Br Dent J. 2004; 197(5): 251-8.
70. Moore S, Ide M, Randhawa M, et al. An investigation into the association among preterm birth, cytokine gene polymorphisms and periodontal disease. BJOG. 2004; 111(2): 125-32.
71. Moore S, Randhawa M, Ide M. A case-control study to investigate an association between adverse pregnancy outcome and periodontal disease. J Clin Periodontol. 2005; 32(1): 1-5.
72. Nabet C, Lelong N, Colombier ML, et al. ; Epipap Group. Maternal periodontitis and the causes of preterm birth: the case-control Epipap study. J Clin Periodontol. 2010; 37(1): 37-45.
73. Noack B, Klingenberg J, Weigelt J, Hoffmann T. Periodontal status and preterm low birth weight: a case control study. J Periodontal Res. 2005; 40(4): 339-45.
74. Offenbacher S, Katz V, Fertik G, et al. J. Periodontal infection as a possible risk factor for preterm low birth weight. J Periodontol. 1996; 67(10 Suppl): 1103-13.\
75. Offenbacher S, Boggess KA, Murtha AP, et al. Progressive periodontal disease and risk of very preterm delivery. Obstet Gynecol. 2006; 107(1): 29-36. Erratum in: Obstet Gynecol. 2006; 107(5): 1171.

76. Patel RB, Batra S, Halemani S, et al. Maternal Periodontitis Prevalence and its Relationship with Preterm and Low-Birth Weight Infants: A Hospital-Based Research. J Pharm Bioallied Sci. 202;16(Suppl 1):S488-S491.
77. Perunovic NDj, Rakic MM, Nikolic LI, et al. The Association Between Periodontal Inflammation and Labor Triggers (Elevated Cytokine Levels) in Preterm Birth: A Cross-Sectional Study. J Periodontol. 2016; 87(3): 248-56.
78. Pitiphat W, Joshipura KJ, Gillman MW, et al. Maternal periodontitis and adverse pregnancy outcomes. Community Dent Oral Epidemiol. 2008; 36(1): 3-11.
79. Pozo E, Mesa F, Ikram MH, Puertas A, et al. Preterm birth and/or low birth weight are associated with periodontal disease and the increased placental immunohistochemical expression of inflammatory markers. Histol Histopathol. 2016; 31(2): 231-7.
80. Pratiksha, G., Neha, A., Anju, H., & Gurvinder, L. Significance of bacterial vaginosis and periodontal infection as Predictors of preterm labor. Bangladesh Journal of Medical Science 2016; 15(3): 441–449.
81. Radochova V, Stepan M, Kacerovska Musilova I, et al. Association between periodontal disease and preterm prelabour rupture of membranes. J Clin Periodontol. 2019; 46(2): 189-196.
82. Rakoto-Alson S, Tenenbaum H, Davideau JL. Periodontal diseases, preterm births, and low birth weight: findings from a homogeneous cohort of women in Madagascar. J Periodontol. 2010; 81(2): 205-213.
83. Reza Karimi M, Hamissi JH, Naeini SR, et al. The Relationship Between Maternal Periodontal Status of and Preterm and Low Birth Weight Infants in Iran: A Case Control Study. Glob J Health Sci. 2015; 8(5): 184-8.
84. Riché EL, Boggess KA, Lieff S, et al. Periodontal disease increases the risk of preterm delivery among preeclamptic women. Ann Periodontol. 2002; 7(1): 95-101.
85. Ryu JI, Oh K, Yang H, et al. Health behaviors, periodontal conditions, and periodontal pathogens in spontaneous preterm birth: a case-control study in Korea. J Periodontol. 2010; 81(6): 855-63.
86. Sánchez AR, Bagniewski S, Weaver AL, Vallejos N. Correlations between maternal periodontal conditions and preterm low birth weight infants. J Int Acad Periodontol. 2007 Apr;9(2):34-41.
87. Santa Cruz I, Herrera D, Martin C, et al. Association between periodontal status and pre-term and/or low-birth weight in Spain: clinical and microbiological parameters. J Periodontal Res. 2013; 48(4): 443-51.
88. Santos-Pereira SA, Giraldo PC, Saba-Chujfi E, et al. Chronic periodontitis and pre-term labour in Brazilian pregnant women: an association to be analysed. J Clin Periodontol. 2007; 34(3): 208–13.
89. Savitha JN, Bhavya B, Yadalam U, Khan SF. Detection of Porphyromonas gingivalis in umbilical cord blood of new-born and in subgingival plaque of pregnant participants

with periodontal disease and its association with pregnancy outcomes: An observational study. J Indian Soc Periodontol. 2022; 26(4): 365-372.
90. Sert T, Kırzıoğlu FY, Fentoğlu O, et al. Serum placental growth factor, vascular endothelial growth factor, soluble vascular endothelial growth factor receptor-1 and -2 levels in periodontal disease, and adverse pregnancy outcomes. J Periodontol. 2011; 82(12): 1735-48.
91. Sharma A, Ramesh A, Thomas B. Evaluation of plasma C-reactive protein levels in pregnant women with and without periodontal disease: A comparative study. J Indian Soc Periodontol. 2009; 13(3): 145-9.
92. Siqueira FM, Cota LO, Costa JE, et al. Intrauterine growth restriction, low birth weight, and preterm birth: adverse pregnancy outcomes and their association with maternal periodontitis. J Periodontol. 2007; 78(12): 2266-2276.
93. Skuldbøl T, Johansen KH, Dahlén G, et al. Is pre-term labour associated with periodontitis in a Danish maternity ward? J Clin Periodontol. 2006; 33(3): 177-83.
94. Soucy-Giguère L, Tétu A, Gauthier S, et al. Periodontal Disease and Adverse Pregnancy Outcomes: A Prospective Study in a Low-Risk Population. J Obstet Gynaecol Can. 2016; 38(4): 346-50.
95. Srinivas SK, Sammel MD, Stamilio DM, et al. Periodontal disease and adverse pregnancy outcomes: is there an association? Am J Obstet Gynecol. 2009; 200(5): 497.e1-8.
96. Sugita N, Kobayashi T, Kikuchi A, et al. Immunoregulatory gene polymorphisms in Japanese women with preterm births and periodontitis. J Reprod Immunol. 2012; 93(2): 94-101.
97. Tang L, Chen K. Association Between Periodontitis and Adverse Pregnancy Outcomes: Two-Sample Mendelian Randomisation Study. Int Dent J. 2024;74(6):1397-1404.
98. Tellapragada C, Eshwara VK, Bhat P, et al. Risk Factors for Preterm Birth and Low Birth Weight Among Pregnant Indian Women: A Hospital-based Prospective Study. J Prev Med Public Health. 2016; 49(3): 165-75.
99. Thomas C, Timofeeva I, Bouchoucha E, et al. Oral and periodontal assessment at the first trimester of pregnancy: The PERISCOPE longitudinal study. Acta Obstet Gynecol Scand. 2023; 102(6): 669-680.
100. Trivedi P, Saxena D, Puwar T, et al. A cohort study on risk factors for preterm births in rural Gujarat. Indian J Public Health. 2018; 62(2): 111-116.
101. Turton M & Africa CWJ. Further evidence for periodontal disease as a risk indicator for adverse pregnancy outcomes. Int Dent J. 2017; 67(3): 148-156.
102. Usin MM, Menso J, Rodríguez VI, et al. Association between maternal periodontitis and preterm and/or low birth weight infants in normal pregnancies. J Matern Fetal Neonatal Med. 2016; 29(1): 115-9.

103. Uwambaye P, Munyanshongore C, Rulisa S, et al. Assessing the association between periodontitis and premature birth: a case-control study. BMC Pregnancy Childbirth. 2021; 21(1): 204.

104. Vettore MV, Leal Md, Leão AT, et al. The relationship between periodontitis and preterm low birthweight. J Dent Res. 2008; 87(1): 73-8.

105. Vogt M, Sallum AW, Cecatti JG, Morais SS. Periodontal disease and some adverse perinatal outcomes in a cohort of low risk pregnant women. Reprod Health. 2010; 7: 29.

106. Wang YL, Liou JD, Pan WL. Association between maternal p%eriodontal disease and preterm delivery and low birth weight. Taiwan J Obstet Gynecol. 2013; 52(1): 71-76.

107. Wood S, Frydman A, Cox S, et al. Periodontal disease and spontaneous preterm birth: a case control study. BMC Pregnancy Childbirth. 2006; 6: 24.

108. Yanaranci S, Laosrisin N, Sriprasertsuk A, Panrin P, Nantakeeratipat T. The Association of Maternal Periodontal Diseases in the Postpartum Period with Preterm Low Birth Weight. J Contemp Dent Pract. 2024 Feb 1;25(2):99-106.

109. Ye C, Xia Z, Tang J, et al. Unculturable and culturable periodontal-related bacteria are associated with periodontal inflammation during pregnancy and with preterm low birth weight delivery. Sci Rep. 2020; 10(1): 15807.

Online Appendix S2A-C

Risk of bias assessment per study design

A. Cohort studies

Newcastle – Ottawa quality assessment scale for cohort studies	Selection (max 4)	Comparability (max 2)	Outcome (max 3)	Score
Pockpa et al. 2022 (39)	★★★★	★★	★★★	9/9
Caneiro et al. 2020 (13)	★★		★★★	5/9
Kumar et al. 2013 (43)	★★★	★★	★★★	8/9
Agueda et al. 2008 (46)	★★★★	★★	★★★	9/9
López et al. 2002 (14)	★★★★	★★	★★★	9/9
Offenbacher et al. 2001 (15)	★★★★	★★	★★★	9/9

B. Cross-sectional studies

Modified Newcastle – Ottawa quality assessment scale for cross-sectional studies	Selection (max 5)	Comparability (max 2)	Outcome (max 3)	Score
Martínez-Martínez et al. 2016 (41)	★★★		★★	5/10

C. Case-control studies

Newcastle – Ottawa quality assessment scale for case control studies	Selection (max 4)	Comparability (max 2)	Exposure (max 3)	Score
Pérez-Molina et al. 2019 (40)	★★★★	★★	★★★	9/9
Macedo et al. 2014 (42)	★★★★	★★	★★★	9/9
Martinez de Teyada et al. 2012 (44)	★★★★	★★	★★	8/9
Piscoya et al. 2012 (45)	★★★	★★	★★★	8/9

Online Appendix S3-A

Meta-analysis, subgroup analysis: forest plot using a random model (REM) of the performed meta-analysis for pregnant woment with periodontitis compared to pregnant women without periodontitis on risk of bias.

Low risk of bias (REM)

Study or Subgroup	Periodontitis Events	Periodontitis Total	Non-Periodontitis Events	Non-Periodontitis Total	Weight	Odds Ratio M-H, Random, 95% CI
**1.2.1 Case-control studies**						
Macedo et al. 2014	16	46	58	250	9.8%	1.77 [0.90, 3.46]
Martinez de Teyada et al. 2012	34	125	50	304	12.2%	1.90 [1.15, 3.12]
Pe´rez-Molina et al. 2019	229	507	114	522	15.5%	2.95 [2.25, 3.87]
Piscoya et al. 2012	70	82	290	636	10.3%	6.96 [3.70, 13.09]
**Subtotal (95% CI)**		**760**		**1712**	**47.7%**	**2.86 [1.73, 4.72]**
Total events	349		512			
Heterogeneity: Tau² = 0.19; Chi² = 12.39, df = 3 (P = 0.006); I² = 76%						
Test for overall effect: Z = 4.11 (P < 0.0001)						
**1.2.2 Cross-sectional studies**						
Martínez-Martínez et al. 2016	19	49	6	21	0.0%	1.58 [0.52, 4.79]
**Subtotal (95% CI)**		**0**		**0**		**Not estimable**
Total events	0		0			
Heterogeneity: Not applicable						
Test for overall effect: Not applicable						
**1.2.3 Cohort studies**						
Agueda et al. 2008	31	338	54	958	12.7%	1.69 [1.07, 2.68]
Caneiro et al. 2020	6	39	12	119	0.0%	1.62 [0.56, 4.66]
Kumar et al. 2013	23	61	24	132	9.7%	2.72 [1.38, 5.38]
Lo´pez et al. 2002	12	233	6	406	6.4%	3.62 [1.34, 9.78]
Offenbacher et al. 2001	150	611	38	201	13.7%	1.40 [0.94, 2.08]
Pockpa et al. 2022	50	201	12	137	9.8%	3.45 [1.76, 6.76]
**Subtotal (95% CI)**		**1444**		**1834**	**52.3%**	**2.15 [1.47, 3.14]**
Total events	266		134			
Heterogeneity: Tau² = 0.09; Chi² = 8.25, df = 4 (P = 0.08); I² = 52%						
Test for overall effect: Z = 3.94 (P < 0.0001)						
**Total (95% CI)**		**2204**		**3546**	**100.0%**	**2.49 [1.82, 3.42]**
Total events	615		646			
Heterogeneity: Tau² = 0.15; Chi² = 26.14, df = 8 (P = 0.0010); I² = 69%						
Test for overall effect: Z = 5.64 (P < 0.00001)						
Test for subgroup differences: Chi² = 0.79, df = 1 (P = 0.37), I² = 0%						

Odds Ratio M-H, Random, 95% CI

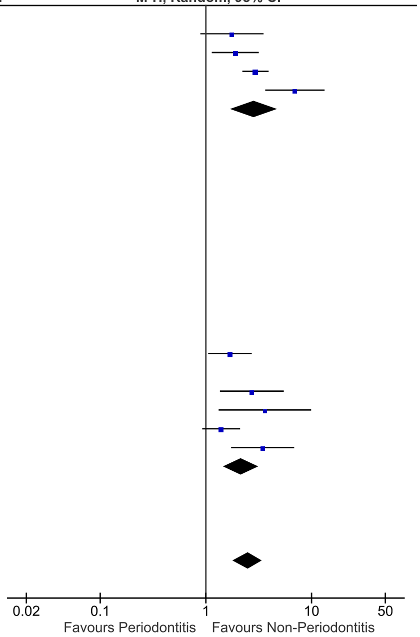

High risk of bias (FEM)

Study or Subgroup	Periodontitis Events	Periodontitis Total	Non-Periodontitis Events	Non-Periodontitis Total	Weight	Odds Ratio M-H, Fixed, 95% CI
**1.2.1 Case-control studies**						
Macedo et al. 2014	16	46	58	250	0.0%	1.77 [0.90, 3.46]
Martinez de Teyada et al. 2012	34	125	50	304	0.0%	1.90 [1.15, 3.12]
Pe´rez-Molina et al. 2019	229	507	114	522	0.0%	2.95 [2.25, 3.87]
Piscoya et al. 2012	70	82	290	636	0.0%	6.96 [3.70, 13.09]
**Subtotal (95% CI)**		**0**		**0**		**Not estimable**
Total events	0		0			
Heterogeneity: Not applicable						
Test for overall effect: Not applicable						
**1.2.2 Cross-sectional studies**						
Martínez-Martínez et al. 2016	19	49	6	21	50.6%	1.58 [0.52, 4.79]
**Subtotal (95% CI)**		**49**		**21**	**50.6%**	**1.58 [0.52, 4.79]**
Total events	19		6			
Heterogeneity: Not applicable						
Test for overall effect: Z = 0.81 (P = 0.42)						
**1.2.3 Cohort studies**						
Agueda et al. 2008	31	338	54	958	0.0%	1.69 [1.07, 2.68]
Caneiro et al. 2020	6	39	12	119	49.4%	1.62 [0.56, 4.66]
Kumar et al. 2013	23	61	24	132	0.0%	2.72 [1.38, 5.38]
Lo´pez et al. 2002	12	233	6	406	0.0%	3.62 [1.34, 9.78]
Offenbacher et al. 2001	150	611	38	201	0.0%	1.40 [0.94, 2.08]
Pockpa et al. 2022	50	201	12	137	0.0%	3.45 [1.76, 6.76]
**Subtotal (95% CI)**		**39**		**119**	**49.4%**	**1.62 [0.56, 4.66]**
Total events	6		12			
Heterogeneity: Not applicable						
Test for overall effect: Z = 0.90 (P = 0.37)						
**Total (95% CI)**		**88**		**140**	**100.0%**	**1.60 [0.75, 3.44]**
Total events	25		18			
Heterogeneity: Chi² = 0.00, df = 1 (P = 0.98); I² = 0%						
Test for overall effect: Z = 1.21 (P = 0.23)						
Test for subgroup differences: Chi² = 0.00, df = 1 (P = 0.98), I² = 0%						

Odds Ratio M-H, Fixed, 95% CI

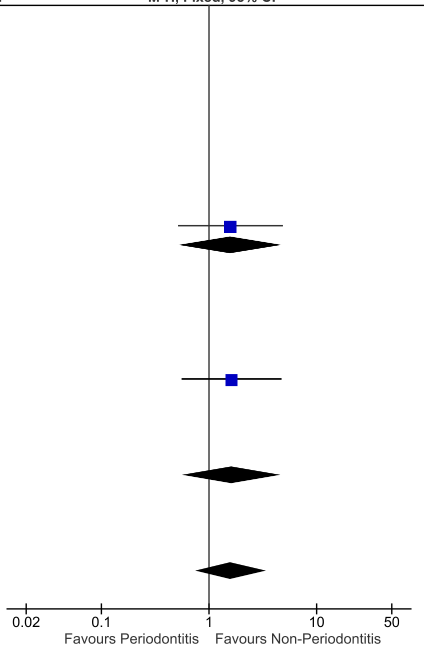

Online Appendix S3-B

Meta-analysis, subgroup analysis: forest plots using a random model (REM) and fixed model (FEM) of the performed meta-analysis for pregnant woment with periodontitis compared to pregnant women without periodontitis on study design.

Case-control (REM)

Study or Subgroup	Periodontitis Events	Periodontitis Total	Non-Periodontitis Events	Non-Periodontitis Total	Weight	Odds Ratio M-H, Random, 95% CI
**1.2.1 Case-control studies**						
Macedo et al. 2014	16	46	58	250	21.1%	1.77 [0.90, 3.46]
Martinez de Teyada et al. 2012	34	125	50	304	25.6%	1.90 [1.15, 3.12]
Pe̍rez-Molina et al. 2019	229	507	114	522	31.1%	2.95 [2.25, 3.87]
Piscoya et al. 2012	70	82	290	636	22.2%	6.96 [3.70, 13.09]
**Subtotal (95% CI)**		**760**		**1712**	**100.0%**	**2.86 [1.73, 4.72]**
Total events	349		512			
Heterogeneity: Tau² = 0.19; Chi² = 12.39, df = 3 (P = 0.006); I² = 76%						
Test for overall effect: Z = 4.11 (P < 0.0001)						
**1.2.2 Cross-sectional studies**						
Martínez-Martínez et al. 2016	19	49	6	21	0.0%	1.58 [0.52, 4.79]
**Subtotal (95% CI)**		**0**		**0**		**Not estimable**
Total events	0		0			
Heterogeneity: Not applicable						
Test for overall effect: Not applicable						
**1.2.3 Cohort studies**						
Agueda et al. 2008	31	338	54	958	0.0%	1.69 [1.07, 2.68]
Caneiro et al. 2020	6	39	12	119	0.0%	1.62 [0.56, 4.66]
Kumar et al. 2013	23	61	24	132	0.0%	2.72 [1.38, 5.38]
Ló́pez et al. 2002	12	233	6	406	0.0%	3.62 [1.34, 9.78]
Offenbacher et al. 2001	150	611	38	201	0.0%	1.40 [0.94, 2.08]
Pockpa et al. 2022	50	201	12	137	0.0%	3.45 [1.76, 6.76]
**Subtotal (95% CI)**		**0**		**0**		**Not estimable**
Total events	0		0			
Heterogeneity: Not applicable						
Test for overall effect: Not applicable						
**Total (95% CI)**		**760**		**1712**	**100.0%**	**2.86 [1.73, 4.72]**
Total events	349		512			
Heterogeneity: Tau² = 0.19; Chi² = 12.39, df = 3 (P = 0.006); I² = 76%						
Test for overall effect: Z = 4.11 (P < 0.0001)						
Test for subgroup differences: Not applicable						

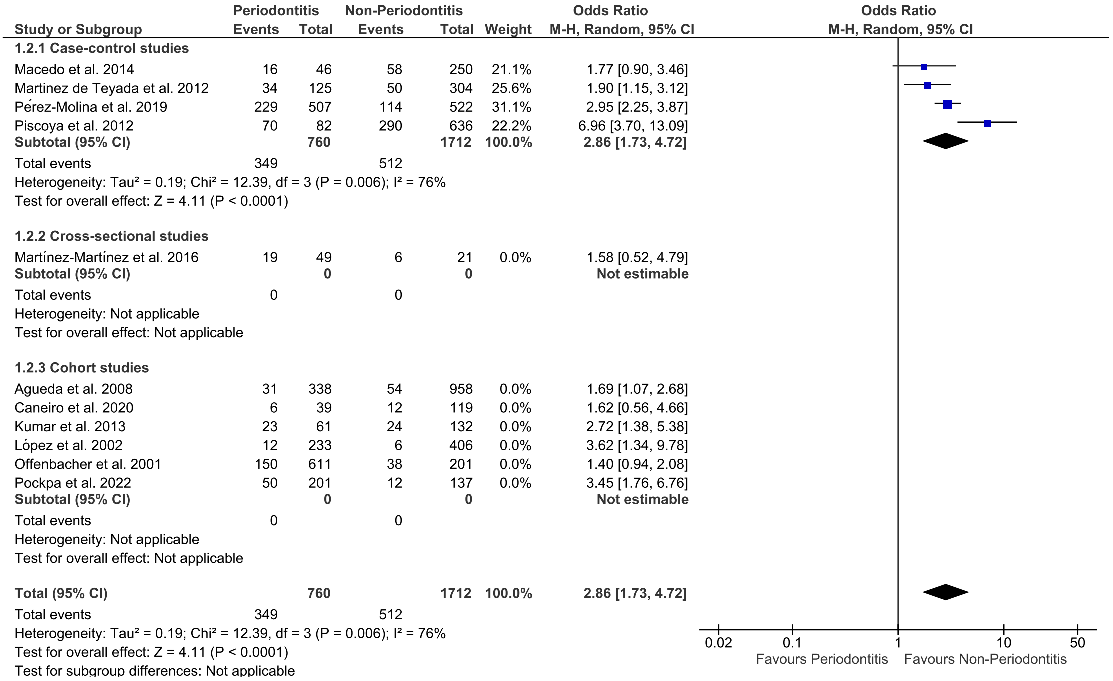

Cohort (REM)

Study or Subgroup	Periodontitis Events	Periodontitis Total	Non-Periodontitis Events	Non-Periodontitis Total	Weight	Odds Ratio M-H, Random, 95% CI
**1.2.1 Case-control studies**						
Macedo et al. 2014	16	46	58	250	0.0%	1.77 [0.90, 3.46]
Martinez de Teyada et al. 2012	34	125	50	304	0.0%	1.90 [1.15, 3.12]
Pe̍rez-Molina et al. 2019	229	507	114	522	0.0%	2.95 [2.25, 3.87]
Piscoya et al. 2012	70	82	290	636	0.0%	6.96 [3.70, 13.09]
**Subtotal (95% CI)**		**0**		**0**		**Not estimable**
Total events	0		0			
Heterogeneity: Not applicable						
Test for overall effect: Not applicable						
**1.2.2 Cross-sectional studies**						
Martínez-Martínez et al. 2016	19	49	6	21	0.0%	1.58 [0.52, 4.79]
**Subtotal (95% CI)**		**0**		**0**		**Not estimable**
Total events	0		0			
Heterogeneity: Not applicable						
Test for overall effect: Not applicable						
**1.2.3 Cohort studies**						
Agueda et al. 2008	31	338	54	958	24.1%	1.69 [1.07, 2.68]
Caneiro et al. 2020	6	39	12	119	8.2%	1.62 [0.56, 4.66]
Kumar et al. 2013	23	61	24	132	15.6%	2.72 [1.38, 5.38]
Ló́pez et al. 2002	12	233	6	406	9.1%	3.62 [1.34, 9.78]
Offenbacher et al. 2001	150	611	38	201	27.1%	1.40 [0.94, 2.08]
Pockpa et al. 2022	50	201	12	137	15.9%	3.45 [1.76, 6.76]
**Subtotal (95% CI)**		**1483**		**1953**	**100.0%**	**2.07 [1.48, 2.89]**
Total events	272		146			
Heterogeneity: Tau² = 0.07; Chi² = 8.35, df = 5 (P = 0.14); I² = 40%						
Test for overall effect: Z = 4.24 (P < 0.0001)						
**Total (95% CI)**		**1483**		**1953**	**100.0%**	**2.07 [1.48, 2.89]**
Total events	272		146			
Heterogeneity: Tau² = 0.07; Chi² = 8.35, df = 5 (P = 0.14); I² = 40%						
Test for overall effect: Z = 4.24 (P < 0.0001)						
Test for subgroup differences: Not applicable						

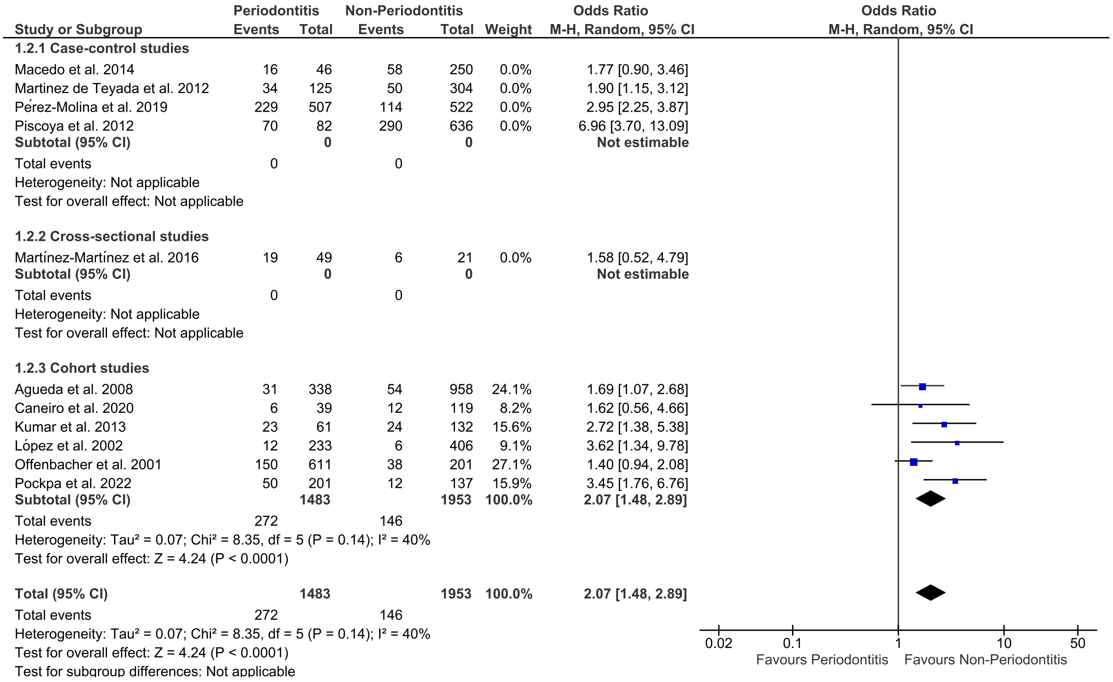

Online Appendix S3-C

Meta-analysis, subgroup analysis: forest plots using a random model (REM) and fixed model (FEM) of the performed meta-analysis for pregnant woment with periodontitis compared to pregnant women without periodontitis on continent.

Europe (FEM)

Study or Subgroup	Periodontitis Events	Periodontitis Total	Non-Periodontitis Events	Non-Periodontitis Total	Weight	Odds Ratio M-H, Fixed, 95% CI
**1.2.1 Case-control studies**						
Macedo et al. 2014	16	46	58	250	0.0%	1.77 [0.90, 3.46]
Martinez de Teyada et al. 2012	34	125	50	304	40.9%	1.90 [1.15, 3.12]
Pérez-Molina et al. 2019	229	507	114	522	0.0%	2.95 [2.25, 3.87]
Piscoya et al. 2012	70	82	290	636	0.0%	6.96 [3.70, 13.09]
**Subtotal (95% CI)**		**125**		**304**	**40.9%**	**1.90 [1.15, 3.12]**
Total events	34		50			
Heterogeneity: Not applicable						
Test for overall effect: Z = 2.53 (P = 0.01)						
**1.2.2 Cross-sectional studies**						
Martínez-Martínez et al. 2016	19	49	6	21	0.0%	1.58 [0.52, 4.79]
**Subtotal (95% CI)**		**0**		**0**		**Not estimable**
Total events	0		0			
Heterogeneity: Not applicable						
Test for overall effect: Not applicable						
**1.2.3 Cohort studies**						
Agueda et al. 2008	31	338	54	958	49.4%	1.69 [1.07, 2.68]
Caneiro et al. 2020	6	39	12	119	9.7%	1.62 [0.56, 4.66]
Kumar et al. 2013	23	61	24	132	0.0%	2.72 [1.38, 5.38]
López et al. 2002	12	233	6	406	0.0%	3.62 [1.34, 9.78]
Offenbacher et al. 2001	150	611	38	201	0.0%	1.40 [0.94, 2.08]
Pockpa et al. 2022	50	201	12	137	0.0%	3.45 [1.76, 6.76]
**Subtotal (95% CI)**		**377**		**1077**	**59.1%**	**1.68 [1.10, 2.56]**
Total events	37		66			
Heterogeneity: Chi^2 = 0.01, df = 1 (P = 0.94); I^2 = 0%						
Test for overall effect: Z = 2.41 (P = 0.02)						
**Total (95% CI)**		**502**		**1381**	**100.0%**	**1.77 [1.28, 2.44]**
Total events	71		116			
Heterogeneity: Chi^2 = 0.14, df = 2 (P = 0.93); I^2 = 0%						
Test for overall effect: Z = 3.48 (P = 0.0005)						
Test for subgroup differences: Chi^2 = 0.14, df = 1 (P = 0.71), I^2 = 0%						

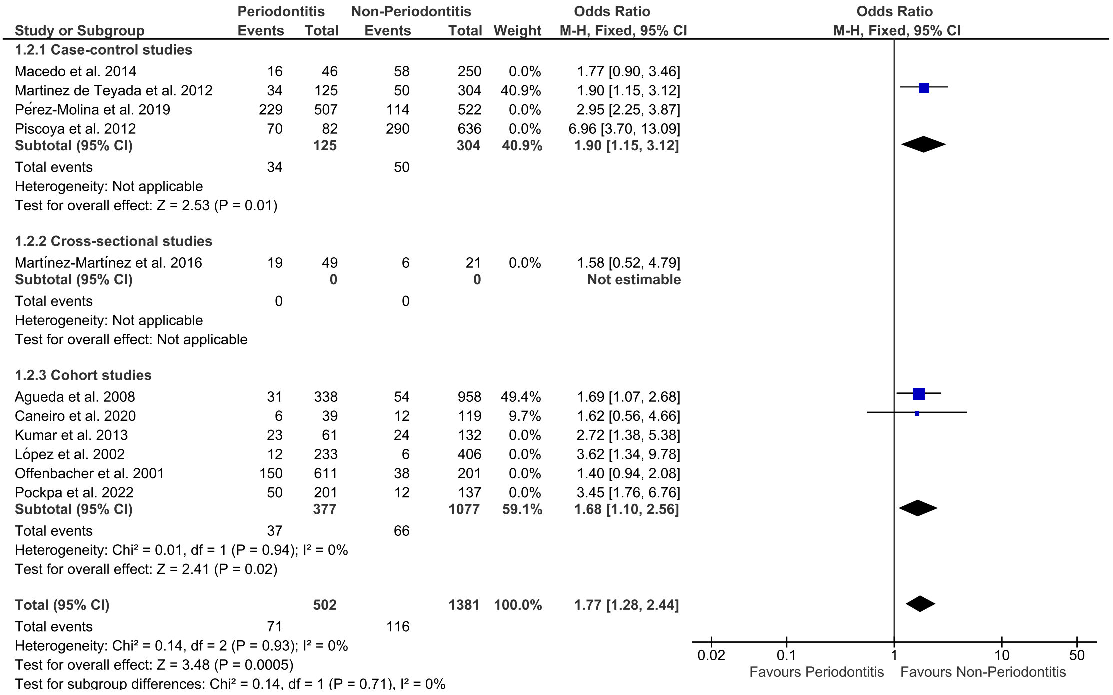

North America (FEM)

Study or Subgroup	Periodontitis Events	Periodontitis Total	Non-Periodontitis Events	Non-Periodontitis Total	Weight	Odds Ratio M-H, Fixed, 95% CI
**1.2.1 Case-control studies**						
Macedo et al. 2014	16	46	58	250	0.0%	1.77 [0.90, 3.46]
Martinez de Teyada et al. 2012	34	125	50	304	0.0%	1.90 [1.15, 3.12]
Pérez-Molina et al. 2019	229	507	114	522	56.1%	2.95 [2.25, 3.87]
Piscoya et al. 2012	70	82	290	636	0.0%	6.96 [3.70, 13.09]
**Subtotal (95% CI)**		**507**		**522**	**56.1%**	**2.95 [2.25, 3.87]**
Total events	229		114			
Heterogeneity: Not applicable						
Test for overall effect: Z = 7.81 (P < 0.00001)						
**1.2.2 Cross-sectional studies**						
Martínez-Martínez et al. 2016	19	49	6	21	4.7%	1.58 [0.52, 4.79]
**Subtotal (95% CI)**		**49**		**21**	**4.7%**	**1.58 [0.52, 4.79]**
Total events	19		6			
Heterogeneity: Not applicable						
Test for overall effect: Z = 0.81 (P = 0.42)						
**1.2.3 Cohort studies**						
Agueda et al. 2008	31	338	54	958	0.0%	1.69 [1.07, 2.68]
Caneiro et al. 2020	6	39	12	119	0.0%	1.62 [0.56, 4.66]
Kumar et al. 2013	23	61	24	132	0.0%	2.72 [1.38, 5.38]
López et al. 2002	12	233	6	406	0.0%	3.62 [1.34, 9.78]
Offenbacher et al. 2001	150	611	38	201	39.3%	1.40 [0.94, 2.08]
Pockpa et al. 2022	50	201	12	137	0.0%	3.45 [1.76, 6.76]
**Subtotal (95% CI)**		**611**		**201**	**39.3%**	**1.40 [0.94, 2.08]**
Total events	150		38			
Heterogeneity: Not applicable						
Test for overall effect: Z = 1.64 (P = 0.10)						
**Total (95% CI)**		**1167**		**744**	**100.0%**	**2.27 [1.82, 2.84]**
Total events	398		158			
Heterogeneity: Chi^2 = 9.69, df = 2 (P = 0.008); I^2 = 79%						
Test for overall effect: Z = 7.29 (P < 0.00001)						
Test for subgroup differences: Chi^2 = 9.69, df = 2 (P = 0.008), I^2 = 79.4%						

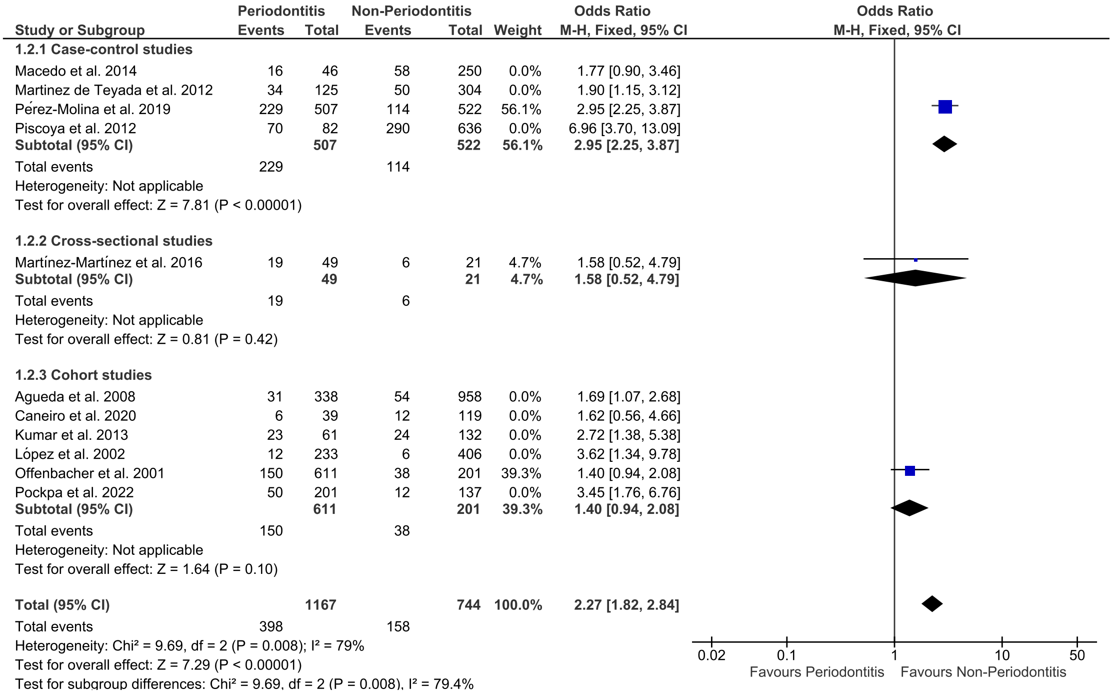

South America (FEM)

Study or Subgroup	Periodontitis Events	Periodontitis Total	Non-Periodontitis Events	Non-Periodontitis Total	Weight	Odds Ratio M-H, Fixed, 95% CI
**1.2.1 Case-control studies**						
Macedo et al. 2014	16	46	58	250	45.9%	1.77 [0.90, 3.46]
Martinez de Teyada et al. 2012	34	125	50	304	0.0%	1.90 [1.15, 3.12]
Pérez-Molina et al. 2019	229	507	114	522	0.0%	2.95 [2.25, 3.87]
Piscoya et al. 2012	70	82	290	636	37.9%	6.96 [3.70, 13.09]
**Subtotal (95% CI)**		**128**		**886**	**83.8%**	**4.11 [2.67, 6.35]**
Total events	86		348			
Heterogeneity: Chi² = 8.71, df = 1 (P = 0.003); I² = 89%						
Test for overall effect: Z = 6.39 (P < 0.00001)						
**1.2.2 Cross-sectional studies**						
Martínez-Martínez et al. 2016	19	49	6	21	0.0%	1.58 [0.52, 4.79]
**Subtotal (95% CI)**		**0**		**0**		**Not estimable**
Total events	0		0			
Heterogeneity: Not applicable						
Test for overall effect: Not applicable						
**1.2.3 Cohort studies**						
Agueda et al. 2008	31	338	54	958	0.0%	1.69 [1.07, 2.68]
Caneiro et al. 2020	6	39	12	119	0.0%	1.62 [0.56, 4.66]
Kumar et al. 2013	23	61	24	132	0.0%	2.72 [1.38, 5.38]
López et al. 2002	12	233	6	406	16.2%	3.62 [1.34, 9.78]
Offenbacher et al. 2001	150	611	38	201	0.0%	1.40 [0.94, 2.08]
Pockpa et al. 2022	50	201	12	137	0.0%	3.45 [1.76, 6.76]
**Subtotal (95% CI)**		**233**		**406**	**16.2%**	**3.62 [1.34, 9.78]**
Total events	12		6			
Heterogeneity: Not applicable						
Test for overall effect: Z = 2.54 (P = 0.01)						
**Total (95% CI)**		**361**		**1292**	**100.0%**	**4.03 [2.71, 6.00]**
Total events	98		354			
Heterogeneity: Chi² = 8.68, df = 2 (P = 0.01); I² = 77%						
Test for overall effect: Z = 6.88 (P < 0.00001)						
Test for subgroup differences: Chi² = 0.05, df = 1 (P = 0.82), I² = 0%						

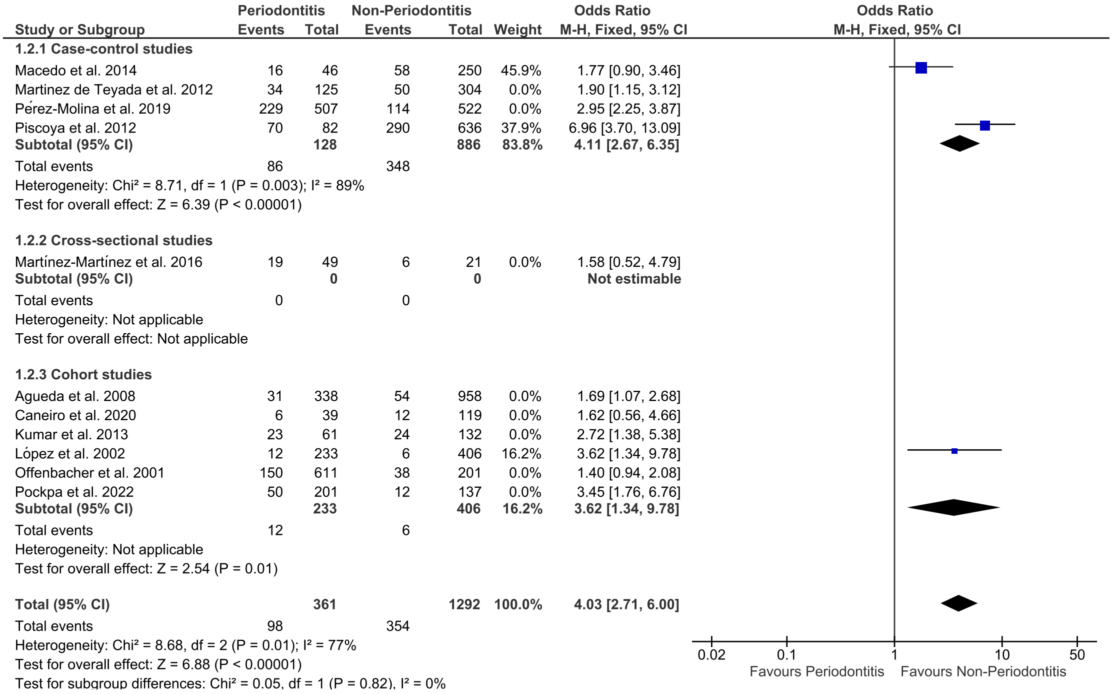

Online Appendix S3-D

Meta-analysis, subgroup analysis: forest plots using a random model (REM) and fixed model (FEM) of the performed meta-analysis for pregnant woment with periodontitis compared to pregnant women without periodontitis on country socio-economic status.

Developed (REM)

Study or Subgroup	Periodontitis Events	Periodontitis Total	Non-Periodontitis Events	Non-Periodontitis Total	Weight	Odds Ratio M-H, Random, 95% CI
**1.2.1 Case-control studies**						
Macedo et al. 2014	16	46	58	250	0.0%	1.77 [0.90, 3.46]
Martinez de Teyada et al. 2012	34	125	50	304	25.3%	1.90 [1.15, 3.12]
Pérez-Molina et al. 2019	229	507	114	522	0.0%	2.95 [2.25, 3.87]
Piscoya et al. 2012	70	82	290	636	0.0%	6.96 [3.70, 13.09]
**Subtotal (95% CI)**		**125**		**304**	**25.3%**	**1.90 [1.15, 3.12]**
Total events	34		50			
Heterogeneity: Not applicable						
Test for overall effect: Z = 2.53 (P = 0.01)						
**1.2.2 Cross-sectional studies**						
Martínez-Martínez et al. 2016	19	49	6	21	0.0%	1.58 [0.52, 4.79]
**Subtotal (95% CI)**		**0**		**0**		**Not estimable**
Total events	0		0			
Heterogeneity: Not applicable						
Test for overall effect: Not applicable						
**1.2.3 Cohort studies**						
Agueda et al. 2008	31	338	54	958	29.6%	1.69 [1.07, 2.68]
Caneiro et al. 2020	6	39	12	119	5.6%	1.62 [0.56, 4.66]
Kumar et al. 2013	23	61	24	132	0.0%	2.72 [1.38, 5.38]
López et al. 2002	12	233	6	406	0.0%	3.62 [1.34, 9.78]
Offenbacher et al. 2001	150	611	38	201	39.5%	1.40 [0.94, 2.08]
Pockpa et al. 2022	50	201	12	137	0.0%	3.45 [1.76, 6.76]
**Subtotal (95% CI)**		**988**		**1278**	**74.7%**	**1.52 [1.14, 2.03]**
Total events	187		104			
Heterogeneity: Tau² = 0.00; Chi² = 0.40, df = 2 (P = 0.82); I² = 0%						
Test for overall effect: Z = 2.85 (P = 0.004)						
**Total (95% CI)**		**1113**		**1582**	**100.0%**	**1.61 [1.25, 2.07]**
Total events	221		154			
Heterogeneity: Tau² = 0.00; Chi² = 0.96, df = 3 (P = 0.81); I² = 0%						
Test for overall effect: Z = 3.73 (P = 0.0002)						
Test for subgroup differences: Chi² = 0.56, df = 1 (P = 0.45), I² = 0%						

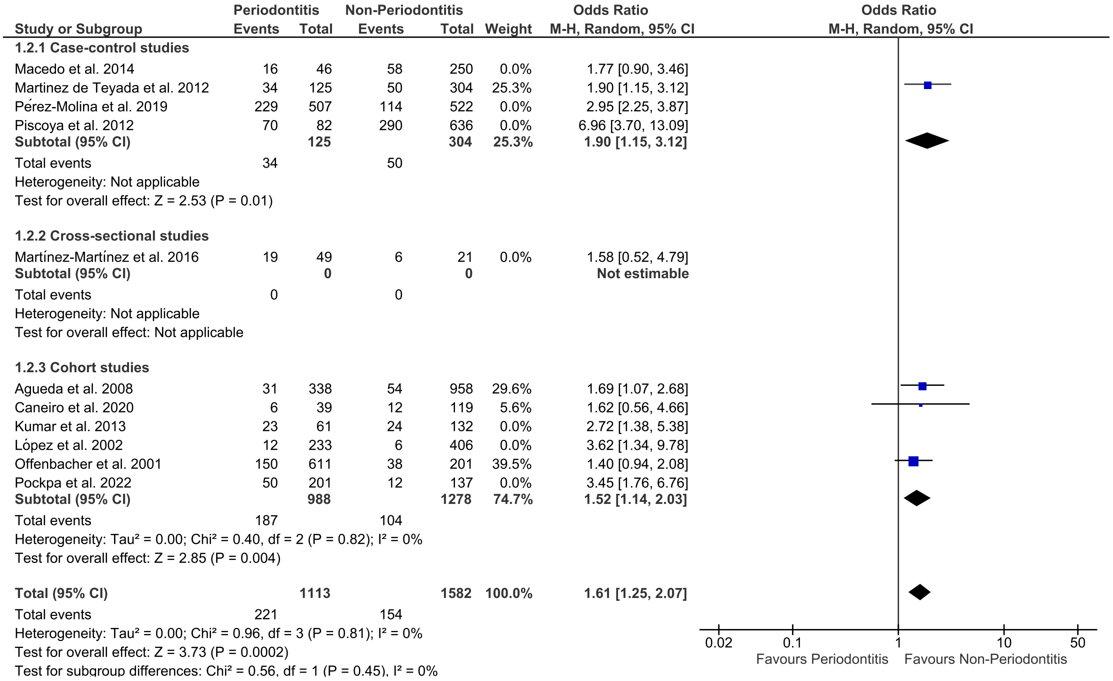

Developing (REM)

Study or Subgroup	Periodontitis Events	Periodontitis Total	Non-Periodontitis Events	Non-Periodontitis Total	Weight	Odds Ratio M-H, Random, 95% CI
**1.2.1 Case-control studies**						
Macedo et al. 2014	16	46	58	250	14.0%	1.77 [0.90, 3.46]
Martinez de Teyada et al. 2012	34	125	50	304	0.0%	1.90 [1.15, 3.12]
Pe´rez-Molina et al. 2019	229	507	114	522	27.9%	2.95 [2.25, 3.87]
Piscoya et al. 2012	70	82	290	636	15.1%	6.96 [3.70, 13.09]
**Subtotal (95% CI)**		**635**		**1408**	**57.0%**	**3.30 [1.74, 6.26]**
Total events	315		462			
Heterogeneity: Tau² = 0.25; Chi² = 9.22, df = 2 (P = 0.010); I² = 78%						
Test for overall effect: Z = 3.64 (P = 0.0003)						
**1.2.2 Cross-sectional studies**						
Martínez-Martínez et al. 2016	19	49	6	21	6.9%	1.58 [0.52, 4.79]
**Subtotal (95% CI)**		**49**		**21**	**6.9%**	**1.58 [0.52, 4.79]**
Total events	19		6			
Heterogeneity: Not applicable						
Test for overall effect: Z = 0.81 (P = 0.42)						
**1.2.3 Cohort studies**						
Agueda et al. 2008	31	338	54	958	0.0%	1.69 [1.07, 2.68]
Caneiro et al. 2020	6	39	12	119	0.0%	1.62 [0.56, 4.66]
Kumar et al. 2013	23	61	24	132	13.8%	2.72 [1.38, 5.38]
Lo´pez et al. 2002	12	233	6	406	8.2%	3.62 [1.34, 9.78]
Offenbacher et al. 2001	150	611	38	201	0.0%	1.40 [0.94, 2.08]
Pockpa et al. 2022	50	201	12	137	14.0%	3.45 [1.76, 6.76]
**Subtotal (95% CI)**		**495**		**675**	**36.1%**	**3.17 [2.06, 4.87]**
Total events	85		42			
Heterogeneity: Tau² = 0.00; Chi² = 0.32, df = 2 (P = 0.85); I² = 0%						
Test for overall effect: Z = 5.24 (P < 0.00001)						
**Total (95% CI)**		**1179**		**2104**	**100.0%**	**3.08 [2.22, 4.26]**
Total events	419		510			
Heterogeneity: Tau² = 0.08; Chi² = 10.93, df = 6 (P = 0.09); I² = 45%						
Test for overall effect: Z = 6.75 (P < 0.00001)						
Test for subgroup differences: Chi² = 1.42, df = 2 (P = 0.49), I² = 0%						

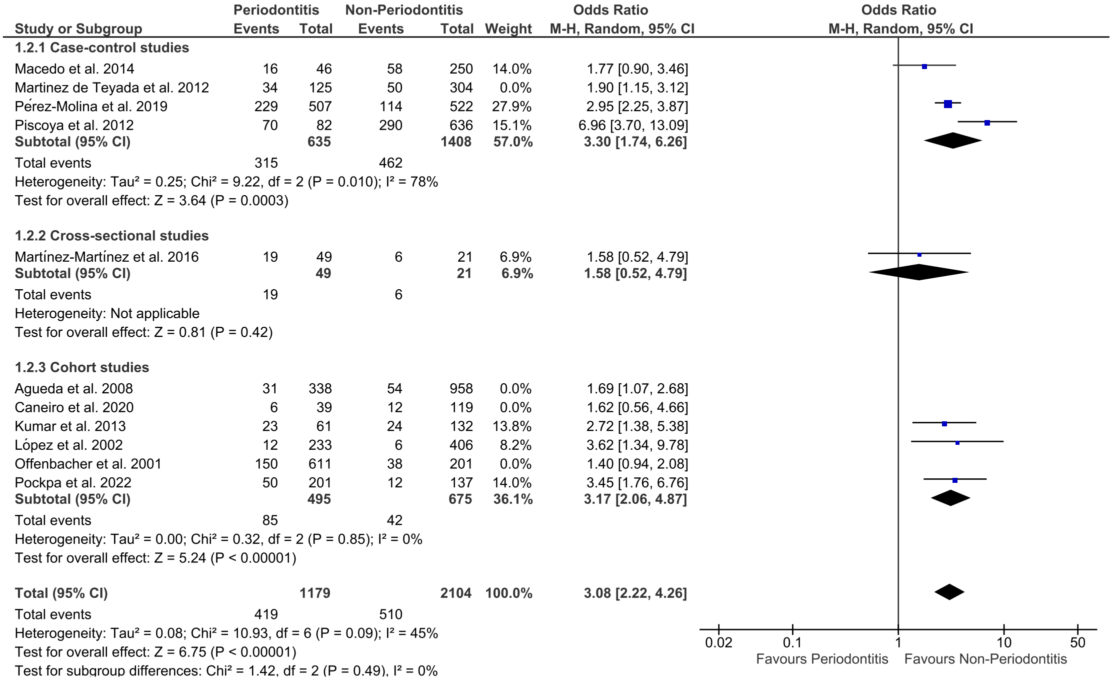

Online Appendix S3-E

Meta-analysis, subgroup analysis: forest plots using a random model (REM) and fixed model (FEM) of the performed meta-analysis for pregnant woment with periodontitis compared to pregnant women without periodontitis on health status, smoking and history of urinary tract infection.

Studies including participants with co-morbidities (REM)

Study or Subgroup	Periodontitis Events	Periodontitis Total	Non-Periodontitis Events	Non-Periodontitis Total	Weight	Odds Ratio M-H, Random, 95% CI
**1.2.1 Case-control studies**						
Macedo et al. 2014	16	46	58	250	0.0%	1.77 [0.90, 3.46]
Martinez de Teyada et al. 2012	34	125	50	304	24.9%	1.90 [1.15, 3.12]
Pe´rez-Molina et al. 2019	229	507	114	522	40.2%	2.95 [2.25, 3.87]
Piscoya et al. 2012	70	82	290	636	0.0%	6.96 [3.70, 13.09]
**Subtotal (95% CI)**		**632**		**826**	**65.1%**	**2.49 [1.64, 3.79]**
Total events	263		164			
Heterogeneity: Tau² = 0.06; Chi² = 2.32, df = 1 (P = 0.13); I² = 57%						
Test for overall effect: Z = 4.26 (P < 0.0001)						
**1.2.2 Cross-sectional studies**						
Martínez-Martínez et al. 2016	19	49	6	21	7.9%	1.58 [0.52, 4.79]
**Subtotal (95% CI)**		**49**		**21**	**7.9%**	**1.58 [0.52, 4.79]**
Total events	19		6			
Heterogeneity: Not applicable						
Test for overall effect: Z = 0.81 (P = 0.42)						
**1.2.3 Cohort studies**						
Agueda et al. 2008	31	338	54	958	27.0%	1.69 [1.07, 2.68]
Caneiro et al. 2020	6	39	12	119	0.0%	1.62 [0.56, 4.66]
Kumar et al. 2013	23	61	24	132	0.0%	2.72 [1.38, 5.38]
Lo´pez et al. 2002	12	233	6	406	0.0%	3.62 [1.34, 9.78]
Offenbacher et al. 2001	150	611	38	201	0.0%	1.40 [0.94, 2.08]
Pockpa et al. 2022	50	201	12	137	0.0%	3.45 [1.76, 6.76]
**Subtotal (95% CI)**		**338**		**958**	**27.0%**	**1.69 [1.07, 2.68]**
Total events	31		54			
Heterogeneity: Not applicable						
Test for overall effect: Z = 2.24 (P = 0.03)						
**Total (95% CI)**		**1019**		**1805**	**100.0%**	**2.16 [1.54, 3.03]**
Total events	313		224			
Heterogeneity: Tau² = 0.05; Chi² = 5.83, df = 3 (P = 0.12); I² = 49%						
Test for overall effect: Z = 4.48 (P < 0.00001)						
Test for subgroup differences: Chi² = 1.71, df = 2 (P = 0.43), I² = 0%						

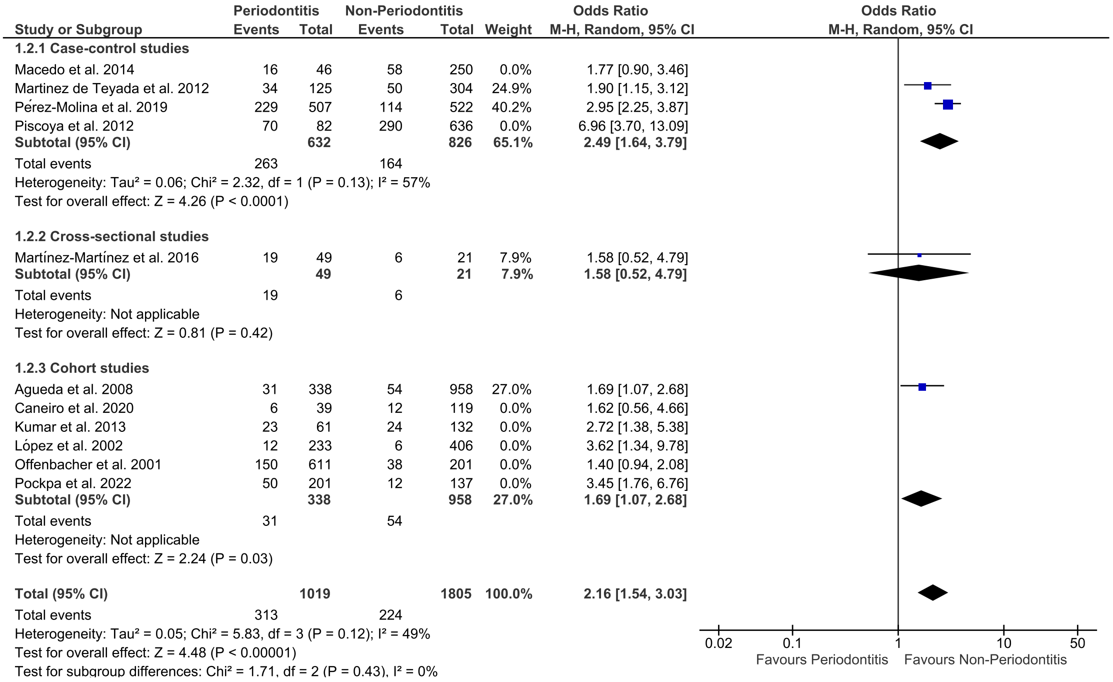

Studies excluding participants with co-morbidities (REM)

Study or Subgroup	Periodontitis Events	Periodontitis Total	Non-Periodontitis Events	Non-Periodontitis Total	Weight	Odds Ratio M-H, Random, 95% CI
**1.2.1 Case-control studies**						
Macedo et al. 2014	16	46	58	250	17.5%	1.77 [0.90, 3.46]
Martinez de Teyada et al. 2012	34	125	50	304	0.0%	1.90 [1.15, 3.12]
Pérez-Molina et al. 2019	229	507	114	522	0.0%	2.95 [2.25, 3.87]
Piscoya et al. 2012	70	82	290	636	18.0%	6.96 [3.70, 13.09]
**Subtotal (95% CI)**		**128**		**886**	**35.5%**	**3.52 [0.90, 13.77]**
Total events	86		348			
Heterogeneity: Tau² = 0.86; Chi² = 8.71, df = 1 (P = 0.003); I² = 89%						
Test for overall effect: Z = 1.81 (P = 0.07)						
**1.2.2 Cross-sectional studies**						
Martínez-Martínez et al. 2016	19	49	6	21	0.0%	1.58 [0.52, 4.79]
**Subtotal (95% CI)**		**0**		**0**		**Not estimable**
Total events	0		0			
Heterogeneity: Not applicable						
Test for overall effect: Not applicable						
**1.2.3 Cohort studies**						
Agueda et al. 2008	31	338	54	958	0.0%	1.69 [1.07, 2.68]
Caneiro et al. 2020	6	39	12	119	12.8%	1.62 [0.56, 4.66]
Kumar et al. 2013	23	61	24	132	17.4%	2.72 [1.38, 5.38]
López et al. 2002	12	233	6	406	13.5%	3.62 [1.34, 9.78]
Offenbacher et al. 2001	150	611	38	201	20.9%	1.40 [0.94, 2.08]
Pockpa et al. 2022	50	201	12	137	0.0%	3.45 [1.76, 6.76]
**Subtotal (95% CI)**		**944**		**858**	**64.5%**	**1.98 [1.25, 3.13]**
Total events	191		80			
Heterogeneity: Tau² = 0.08; Chi² = 4.93, df = 3 (P = 0.18); I² = 39%						
Test for overall effect: Z = 2.93 (P = 0.003)						
**Total (95% CI)**		**1072**		**1744**	**100.0%**	**2.53 [1.45, 4.42]**
Total events	277		428			
Heterogeneity: Tau² = 0.35; Chi² = 20.05, df = 5 (P = 0.001); I² = 75%						
Test for overall effect: Z = 3.25 (P = 0.001)						
Test for subgroup differences: Chi² = 0.62, df = 1 (P = 0.43), I² = 0%						

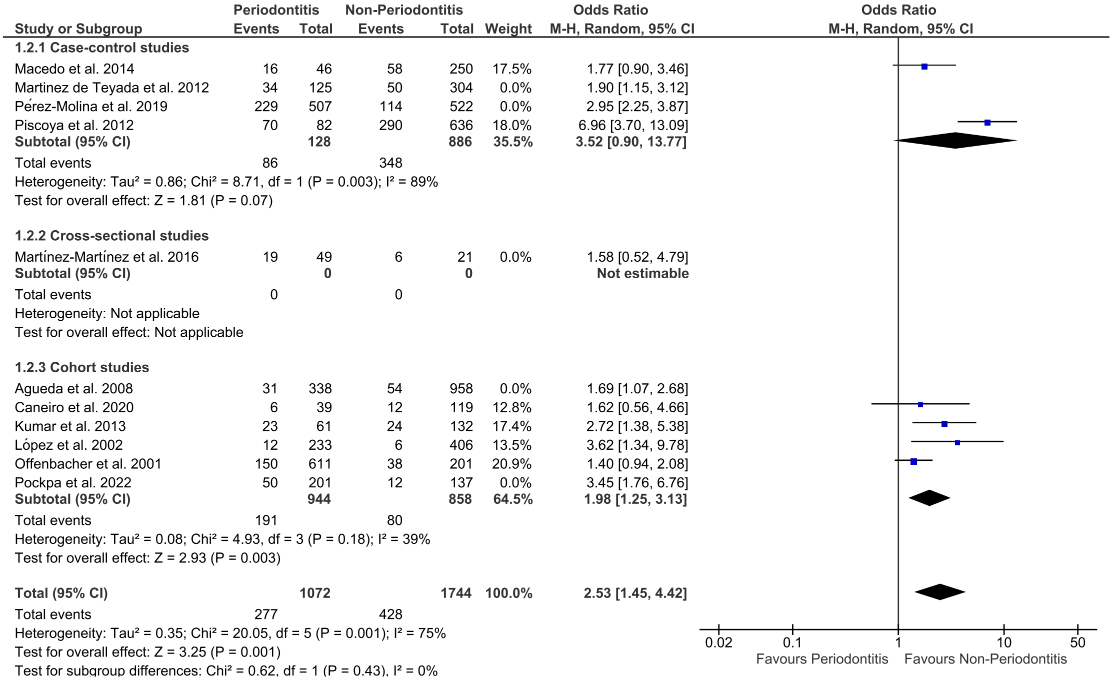

Studies including smokers (REM)

Study or Subgroup	Periodontitis Events	Periodontitis Total	Non-Periodontitis Events	Non-Periodontitis Total	Weight	Odds Ratio M-H, Random, 95% CI
**1.2.1 Case-control studies**						
Macedo et al. 2014	16	46	58	250	0.0%	1.77 [0.90, 3.46]
Martinez de Teyada et al. 2012	34	125	50	304	14.0%	1.90 [1.15, 3.12]
Pérez-Molina et al. 2019	229	507	114	522	17.2%	2.95 [2.25, 3.87]
Piscoya et al. 2012	70	82	290	636	12.1%	6.96 [3.70, 13.09]
**Subtotal (95% CI)**		**714**		**1462**	**43.3%**	**3.26 [1.80, 5.91]**
Total events	333		454			
Heterogeneity: Tau² = 0.22; Chi² = 10.21, df = 2 (P = 0.006); I² = 80%						
Test for overall effect: Z = 3.90 (P < 0.0001)						
**1.2.2 Cross-sectional studies**						
Martínez-Martínez et al. 2016	19	49	6	21	0.0%	1.58 [0.52, 4.79]
**Subtotal (95% CI)**		**0**		**0**		**Not estimable**
Total events	0		0			
Heterogeneity: Not applicable						
Test for overall effect: Not applicable						
**1.2.3 Cohort studies**						
Agueda et al. 2008	31	338	54	958	14.6%	1.69 [1.07, 2.68]
Caneiro et al. 2020	6	39	12	119	7.3%	1.62 [0.56, 4.66]
Kumar et al. 2013	23	61	24	132	0.0%	2.72 [1.38, 5.38]
López et al. 2002	12	233	6	406	7.8%	3.62 [1.34, 9.78]
Offenbacher et al. 2001	150	611	38	201	15.5%	1.40 [0.94, 2.08]
Pockpa et al. 2022	50	201	12	137	11.5%	3.45 [1.76, 6.76]
**Subtotal (95% CI)**		**1422**		**1821**	**56.7%**	**1.98 [1.35, 2.89]**
Total events	249		122			
Heterogeneity: Tau² = 0.08; Chi² = 7.17, df = 4 (P = 0.13); I² = 44%						
Test for overall effect: Z = 3.53 (P = 0.0004)						
**Total (95% CI)**		**2136**		**3283**	**100.0%**	**2.50 [1.74, 3.59]**
Total events	582		576			
Heterogeneity: Tau² = 0.18; Chi² = 25.75, df = 7 (P = 0.0006); I² = 73%						
Test for overall effect: Z = 4.97 (P < 0.00001)						
Test for subgroup differences: Chi² = 1.94, df = 1 (P = 0.16), I² = 48.6%						

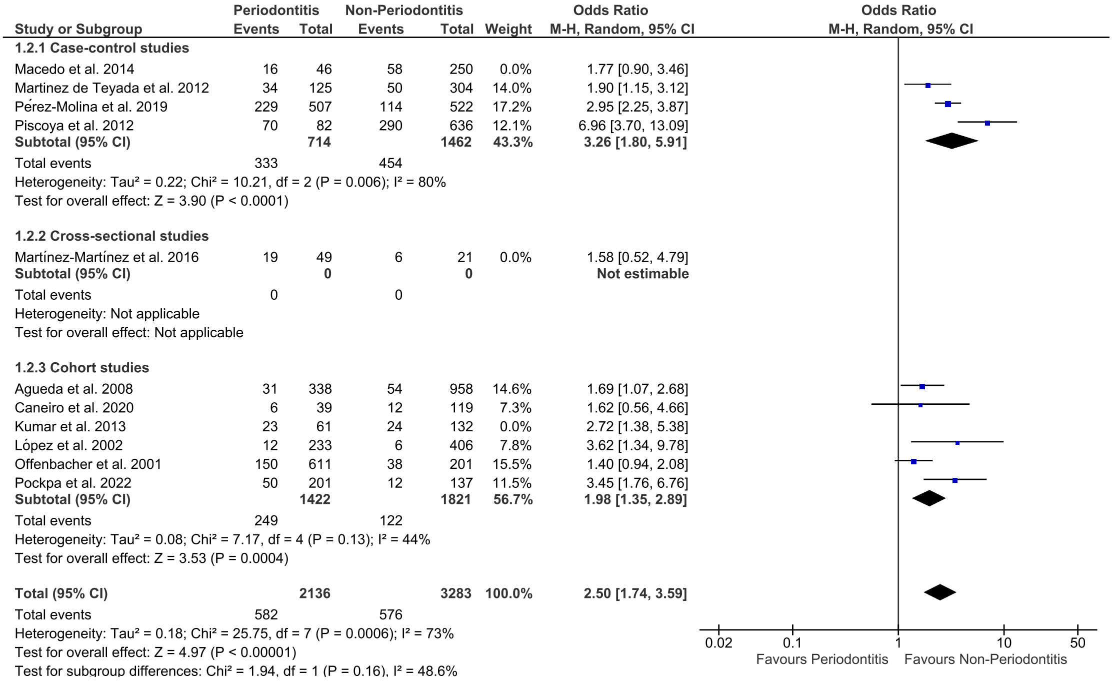

Studies excluding smokers (FEM)

Study or Subgroup	Periodontitis Events	Periodontitis Total	Non-Periodontitis Events	Non-Periodontitis Total	Weight	Odds Ratio M-H, Fixed, 95% CI
**1.2.1 Case-control studies**						
Macedo et al. 2014	16	46	58	250	69.6%	1.77 [0.90, 3.46]
Martinez de Teyada et al. 2012	34	125	50	304	0.0%	1.90 [1.15, 3.12]
Pérez-Molina et al. 2019	229	507	114	522	0.0%	2.95 [2.25, 3.87]
Piscoya et al. 2012	70	82	290	636	0.0%	6.96 [3.70, 13.09]
**Subtotal (95% CI)**		**46**		**250**	**69.6%**	**1.77 [0.90, 3.46]**
Total events	16		58			
Heterogeneity: Not applicable						
Test for overall effect: Z = 1.65 (P = 0.10)						
**1.2.2 Cross-sectional studies**						
Martínez-Martínez et al. 2016	19	49	6	21	30.4%	1.58 [0.52, 4.79]
**Subtotal (95% CI)**		**49**		**21**	**30.4%**	**1.58 [0.52, 4.79]**
Total events	19		6			
Heterogeneity: Not applicable						
Test for overall effect: Z = 0.81 (P = 0.42)						
**1.2.3 Cohort studies**						
Agueda et al. 2008	31	338	54	958	0.0%	1.69 [1.07, 2.68]
Caneiro et al. 2020	6	39	12	119	0.0%	1.62 [0.56, 4.66]
Kumar et al. 2013	23	61	24	132	0.0%	2.72 [1.38, 5.38]
López et al. 2002	12	233	6	406	0.0%	3.62 [1.34, 9.78]
Offenbacher et al. 2001	150	611	38	201	0.0%	1.40 [0.94, 2.08]
Pockpa et al. 2022	50	201	12	137	0.0%	3.45 [1.76, 6.76]
**Subtotal (95% CI)**		**0**		**0**		**Not estimable**
Total events	0		0			
Heterogeneity: Not applicable						
Test for overall effect: Not applicable						
**Total (95% CI)**		**95**		**271**	**100.0%**	**1.71 [0.96, 3.05]**
Total events	35		64			
Heterogeneity: Chi² = 0.03, df = 1 (P = 0.87); I² = 0%						
Test for overall effect: Z = 1.82 (P = 0.07)						
Test for subgroup differences: Chi² = 0.03, df = 1 (P = 0.87), I² = 0%						

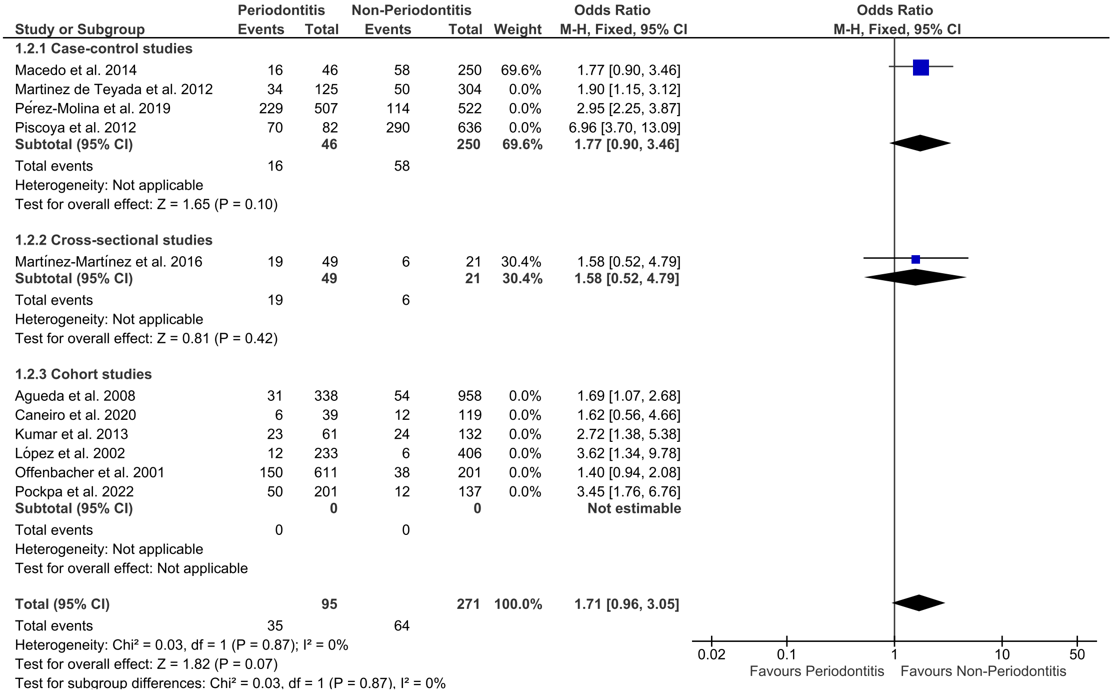

Studies including participants with history of urinary tract infection (REM)

Study or Subgroup	Periodontitis Events	Periodontitis Total	Non-Periodontitis Events	Non-Periodontitis Total	Weight	Odds Ratio M-H, Random, 95% CI
**1.2.1 Case-control studies**						
Macedo et al. 2014	16	46	58	250	12.4%	1.77 [0.90, 3.46]
Martinez de Teyada et al. 2012	34	125	50	304	15.1%	1.90 [1.15, 3.12]
Pérez-Molina et al. 2019	229	507	114	522	18.5%	2.95 [2.25, 3.87]
Piscoya et al. 2012	70	82	290	636	13.1%	6.96 [3.70, 13.09]
**Subtotal (95% CI)**		**760**		**1712**	**59.1%**	**2.86 [1.73, 4.72]**
Total events	349		512			
Heterogeneity: Tau² = 0.19; Chi² = 12.39, df = 3 (P = 0.006); I² = 76%						
Test for overall effect: Z = 4.11 (P < 0.0001)						
**1.2.2 Cross-sectional studies**						
Martínez-Martínez et al. 2016	19	49	6	21	0.0%	1.58 [0.52, 4.79]
**Subtotal (95% CI)**		**0**		**0**		**Not estimable**
Total events	0		0			
Heterogeneity: Not applicable						
Test for overall effect: Not applicable						
**1.2.3 Cohort studies**						
Agueda et al. 2008	31	338	54	958	15.7%	1.69 [1.07, 2.68]
Caneiro et al. 2020	6	39	12	119	0.0%	1.62 [0.56, 4.66]
Kumar et al. 2013	23	61	24	132	0.0%	2.72 [1.38, 5.38]
López et al. 2002	12	233	6	406	8.5%	3.62 [1.34, 9.78]
Offenbacher et al. 2001	150	611	38	201	16.7%	1.40 [0.94, 2.08]
Pockpa et al. 2022	50	201	12	137	0.0%	3.45 [1.76, 6.76]
**Subtotal (95% CI)**		**1182**		**1565**	**40.9%**	**1.71 [1.16, 2.51]**
Total events	193		98			
Heterogeneity: Tau² = 0.04; Chi² = 3.08, df = 2 (P = 0.21); I² = 35%						
Test for overall effect: Z = 2.71 (P = 0.007)						
**Total (95% CI)**		**1942**		**3277**	**100.0%**	**2.38 [1.63, 3.49]**
Total events	542		610			
Heterogeneity: Tau² = 0.19; Chi² = 24.82, df = 6 (P = 0.0004); I² = 76%						
Test for overall effect: Z = 4.46 (P < 0.00001)						
Test for subgroup differences: Chi² = 2.56, df = 1 (P = 0.11), I² = 61.0%						

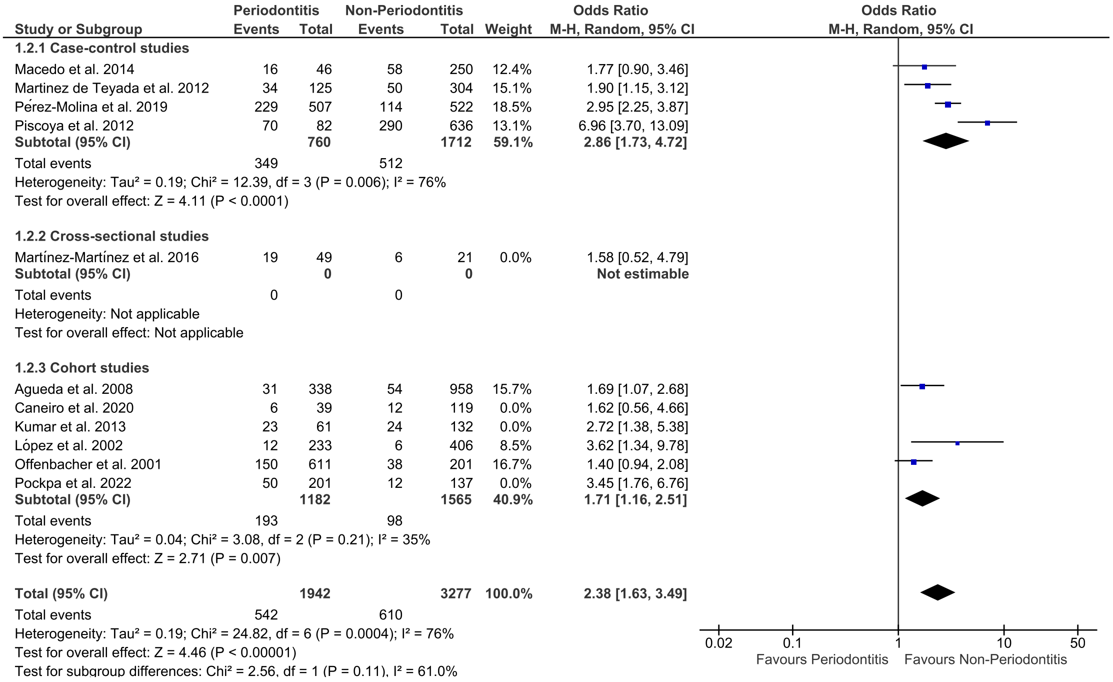

Online Appendix S3-F

Meta-analysis, subgroup analysis: forest plots using a random model (REM) and fixed model (FEM) of the performed meta-analysis for pregnant woment with periodontitis compared to pregnant women without according to periodontitis case definition.

≥ 4 teeth with PPD ≥ 4 mm and CAL ≥ 3 mm at the same site (REM)

Study or Subgroup	Periodontitis Events	Periodontitis Total	Non-Periodontitis Events	Non-Periodontitis Total	Weight	Odds Ratio M-H, Random, 95% CI
**1.2.1 Case-control studies**						
Macedo et al. 2014	16	46	58	250	18.3%	1.77 [0.90, 3.46]
Martinez de Teyada et al. 2012	34	125	50	304	0.0%	1.90 [1.15, 3.12]
Pérez-Molina et al. 2019	229	507	114	522	26.9%	2.95 [2.25, 3.87]
Piscoya et al. 2012	70	82	290	636	19.2%	6.96 [3.70, 13.09]
**Subtotal (95% CI)**		**635**		**1408**	**64.4%**	**3.30 [1.74, 6.26]**
Total events	315		462			
Heterogeneity: Tau² = 0.25; Chi² = 9.22, df = 2 (P = 0.010); I² = 78%						
Test for overall effect: Z = 3.64 (P = 0.0003)						
**1.2.2 Cross-sectional studies**						
Martínez-Martínez et al. 2016	19	49	6	21	0.0%	1.58 [0.52, 4.79]
**Subtotal (95% CI)**		**0**		**0**		**Not estimable**
Total events	0		0			
Heterogeneity: Not applicable						
Test for overall effect: Not applicable						
**1.2.3 Cohort studies**						
Agueda et al. 2008	31	338	54	958	23.0%	1.69 [1.07, 2.68]
Caneiro et al. 2020	6	39	12	119	0.0%	1.62 [0.56, 4.66]
Kumar et al. 2013	23	61	24	132	0.0%	2.72 [1.38, 5.38]
López et al. 2002	12	233	6	406	12.6%	3.62 [1.34, 9.78]
Offenbacher et al. 2001	150	611	38	201	0.0%	1.40 [0.94, 2.08]
Pockpa et al. 2022	50	201	12	137	0.0%	3.45 [1.76, 6.76]
**Subtotal (95% CI)**		**571**		**1364**	**35.6%**	**2.17 [1.08, 4.37]**
Total events	43		60			
Heterogeneity: Tau² = 0.13; Chi² = 1.86, df = 1 (P = 0.17); I² = 46%						
Test for overall effect: Z = 2.16 (P = 0.03)						
**Total (95% CI)**		**1206**		**2772**	**100.0%**	**2.86 [1.79, 4.56]**
Total events	358		522			
Heterogeneity: Tau² = 0.19; Chi² = 14.97, df = 4 (P = 0.005); I² = 73%						
Test for overall effect: Z = 4.41 (P < 0.0001)						
Test for subgroup differences: Chi² = 0.75, df = 1 (P = 0.39), I² = 0%						

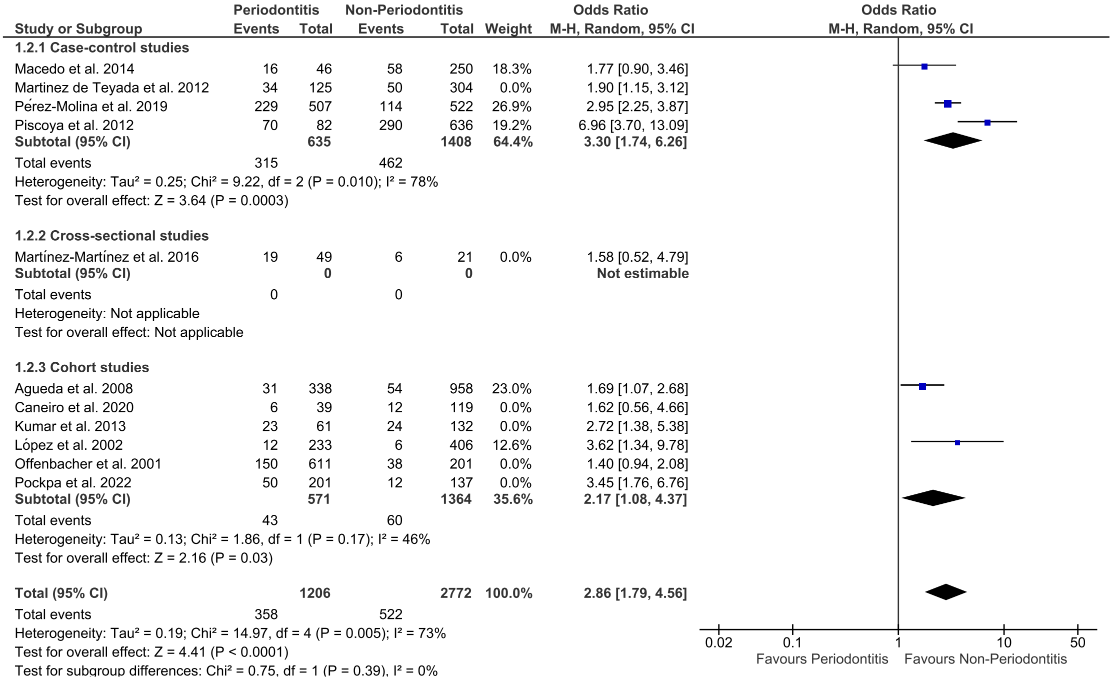

≥ 4 mm CAL (FEM)

Study or Subgroup	Periodontitis Events	Periodontitis Total	Non-Periodontitis Events	Non-Periodontitis Total	Weight	Odds Ratio M-H, Fixed, 95% CI
**1.2.1 Case-control studies**						
Macedo et al. 2014	16	46	58	250	0.0%	1.77 [0.90, 3.46]
Martinez de Teyada et al. 2012	34	125	50	304	69.2%	1.90 [1.15, 3.12]
Pérez-Molina et al. 2019	229	507	114	522	0.0%	2.95 [2.25, 3.87]
Piscoya et al. 2012	70	82	290	636	0.0%	6.96 [3.70, 13.09]
**Subtotal (95% CI)**		**125**		**304**	**69.2%**	**1.90 [1.15, 3.12]**
Total events	34		50			
Heterogeneity: Not applicable						
Test for overall effect: Z = 2.53 (P = 0.01)						
**1.2.2 Cross-sectional studies**						
Martínez-Martínez et al. 2016	19	49	6	21	0.0%	1.58 [0.52, 4.79]
**Subtotal (95% CI)**		**0**		**0**		**Not estimable**
Total events	0		0			
Heterogeneity: Not applicable						
Test for overall effect: Not applicable						
**1.2.3 Cohort studies**						
Agueda et al. 2008	31	338	54	958	0.0%	1.69 [1.07, 2.68]
Caneiro et al. 2020	6	39	12	119	0.0%	1.62 [0.56, 4.66]
Kumar et al. 2013	23	61	24	132	30.8%	2.72 [1.38, 5.38]
López et al. 2002	12	233	6	406	0.0%	3.62 [1.34, 9.78]
Offenbacher et al. 2001	150	611	38	201	0.0%	1.40 [0.94, 2.08]
Pockpa et al. 2022	50	201	12	137	0.0%	3.45 [1.76, 6.76]
**Subtotal (95% CI)**		**61**		**132**	**30.8%**	**2.72 [1.38, 5.38]**
Total events	23		24			
Heterogeneity: Not applicable						
Test for overall effect: Z = 2.88 (P = 0.004)						
**Total (95% CI)**		**186**		**436**	**100.0%**	**2.15 [1.44, 3.21]**
Total events	57		74			
Heterogeneity: Chi² = 0.70, df = 1 (P = 0.40); I² = 0%						
Test for overall effect: Z = 3.75 (P = 0.0002)						
Test for subgroup differences: Chi² = 0.70, df = 1 (P = 0.40), I² = 0%						

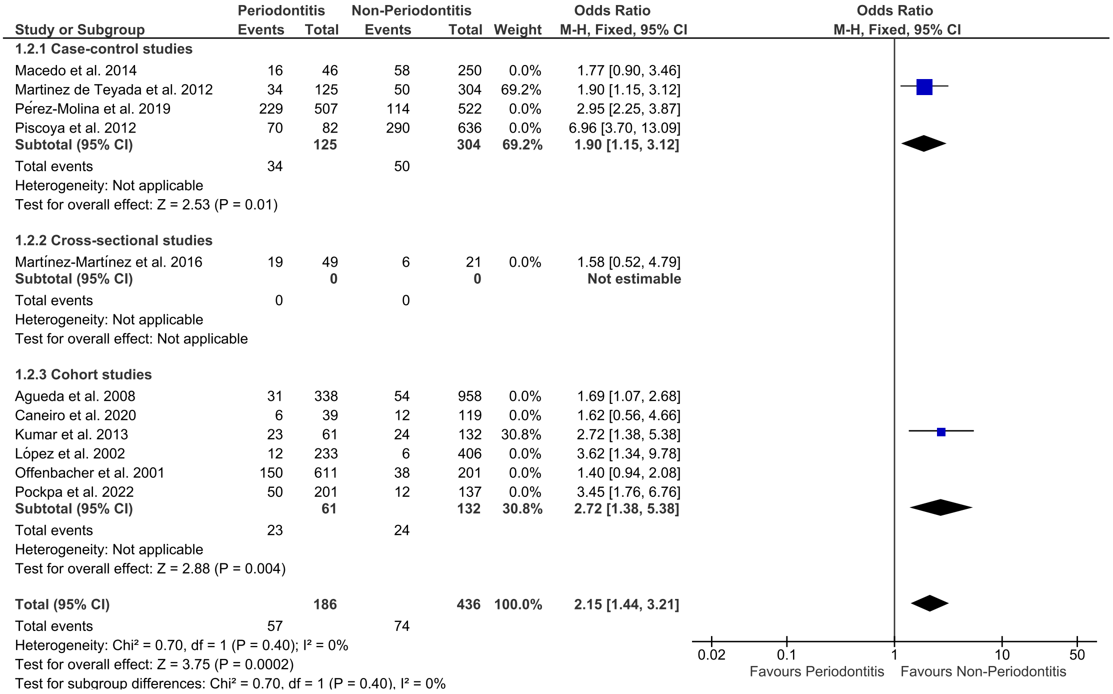

≥ 3 mm CAL (REM)

Study or Subgroup	Periodontitis Events	Periodontitis Total	Non-Periodontitis Events	Non-Periodontitis Total	Weight	Odds Ratio M-H, Random, 95% CI
**1.2.1 Case-control studies**						
Macedo et al. 2014	16	46	58	250	15.0%	1.77 [0.90, 3.46]
Martinez de Teyada et al. 2012	34	125	50	304	0.0%	1.90 [1.15, 3.12]
Pérez-Molina et al. 2019	229	507	114	522	21.0%	2.95 [2.25, 3.87]
Piscoya et al. 2012	70	82	290	636	15.6%	6.96 [3.70, 13.09]
**Subtotal (95% CI)**		**635**		**1408**	**51.7%**	**3.30 [1.74, 6.26]**
Total events	315		462			
Heterogeneity: Tau² = 0.25; Chi² = 9.22, df = 2 (P = 0.010); I² = 78%						
Test for overall effect: Z = 3.64 (P = 0.0003)						
**1.2.2 Cross-sectional studies**						
Martínez-Martínez et al. 2016	19	49	6	21	0.0%	1.58 [0.52, 4.79]
**Subtotal (95% CI)**		**0**		**0**		**Not estimable**
Total events	0		0			
Heterogeneity: Not applicable						
Test for overall effect: Not applicable						
**1.2.3 Cohort studies**						
Agueda et al. 2008	31	338	54	958	18.3%	1.69 [1.07, 2.68]
Caneiro et al. 2020	6	39	12	119	0.0%	1.62 [0.56, 4.66]
Kumar et al. 2013	23	61	24	132	0.0%	2.72 [1.38, 5.38]
López et al. 2002	12	233	6	406	10.7%	3.62 [1.34, 9.78]
Offenbacher et al. 2001	150	611	38	201	19.3%	1.40 [0.94, 2.08]
Pockpa et al. 2022	50	201	12	137	0.0%	3.45 [1.76, 6.76]
**Subtotal (95% CI)**		**1182**		**1565**	**48.3%**	**1.71 [1.16, 2.51]**
Total events	193		98			
Heterogeneity: Tau² = 0.04; Chi² = 3.08, df = 2 (P = 0.21); I² = 35%						
Test for overall effect: Z = 2.71 (P = 0.007)						
**Total (95% CI)**		**1817**		**2973**	**100.0%**	**2.50 [1.60, 3.90]**
Total events	508		560			
Heterogeneity: Tau² = 0.23; Chi² = 24.03, df = 5 (P = 0.0002); I² = 79%						
Test for overall effect: Z = 4.02 (P < 0.0001)						
Test for subgroup differences: Chi² = 2.97, df = 1 (P = 0.08), I² = 66.3%						

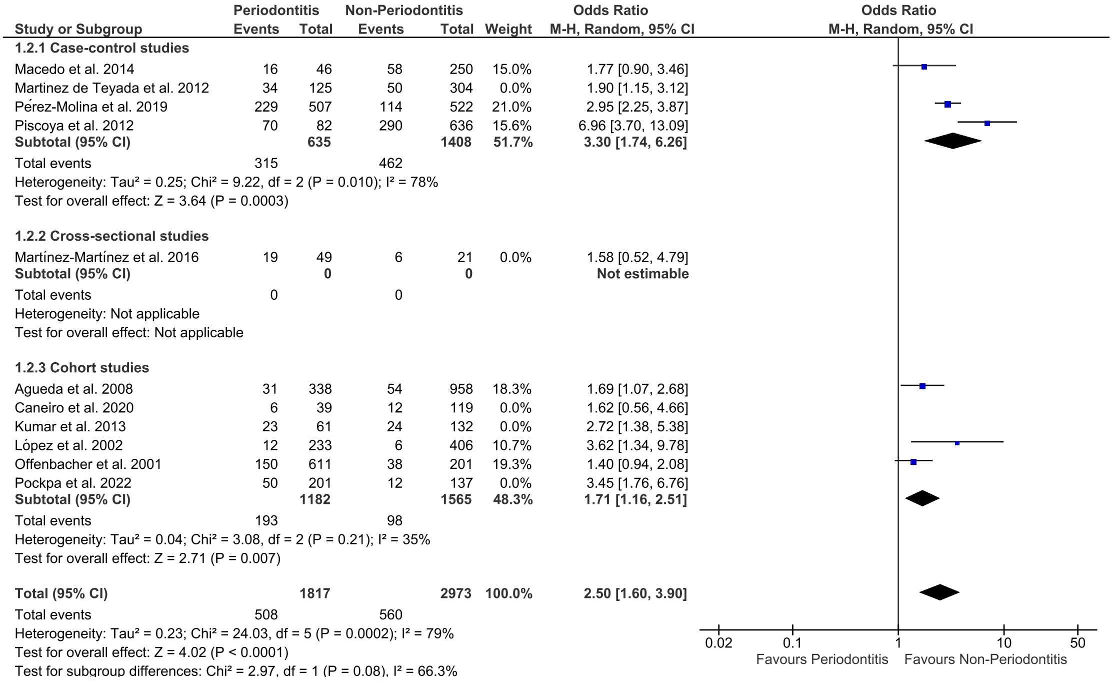

≥ 1 mm CAL (FEM)

Study or Subgroup	Periodontitis Events	Periodontitis Total	Non-Periodontitis Events	Non-Periodontitis Total	Weight	Odds Ratio M-H, Fixed, 95% CI
**1.2.1 Case-control studies**						
Macedo et al. 2014	16	46	58	250	0.0%	1.77 [0.90, 3.46]
Martinez de Teyada et al. 2012	34	125	50	304	0.0%	1.90 [1.15, 3.12]
Pérez-Molina et al. 2019	229	507	114	522	0.0%	2.95 [2.25, 3.87]
Piscoya et al. 2012	70	82	290	636	0.0%	6.96 [3.70, 13.09]
**Subtotal (95% CI)**		**0**		**0**		**Not estimable**
Total events	0		0			
Heterogeneity: Not applicable						
Test for overall effect: Not applicable						
**1.2.2 Cross-sectional studies**						
Martínez-Martínez et al. 2016	19	49	6	21	0.0%	1.58 [0.52, 4.79]
**Subtotal (95% CI)**		**0**		**0**		**Not estimable**
Total events	0		0			
Heterogeneity: Not applicable						
Test for overall effect: Not applicable						
**1.2.3 Cohort studies**						
Agueda et al. 2008	31	338	54	958	0.0%	1.69 [1.07, 2.68]
Caneiro et al. 2020	6	39	12	119	31.9%	1.62 [0.56, 4.66]
Kumar et al. 2013	23	61	24	132	0.0%	2.72 [1.38, 5.38]
López et al. 2002	12	233	6	406	0.0%	3.62 [1.34, 9.78]
Offenbacher et al. 2001	150	611	38	201	0.0%	1.40 [0.94, 2.08]
Pockpa et al. 2022	50	201	12	137	68.1%	3.45 [1.76, 6.76]
**Subtotal (95% CI)**		**240**		**256**	**100.0%**	**2.87 [1.65, 4.99]**
Total events	56		24			
Heterogeneity: Chi² = 1.41, df = 1 (P = 0.23); I² = 29%						
Test for overall effect: Z = 3.72 (P = 0.0002)						
**Total (95% CI)**		**240**		**256**	**100.0%**	**2.87 [1.65, 4.99]**
Total events	56		24			
Heterogeneity: Chi² = 1.41, df = 1 (P = 0.23); I² = 29%						
Test for overall effect: Z = 3.72 (P = 0.0002)						
Test for subgroup differences: Not applicable						

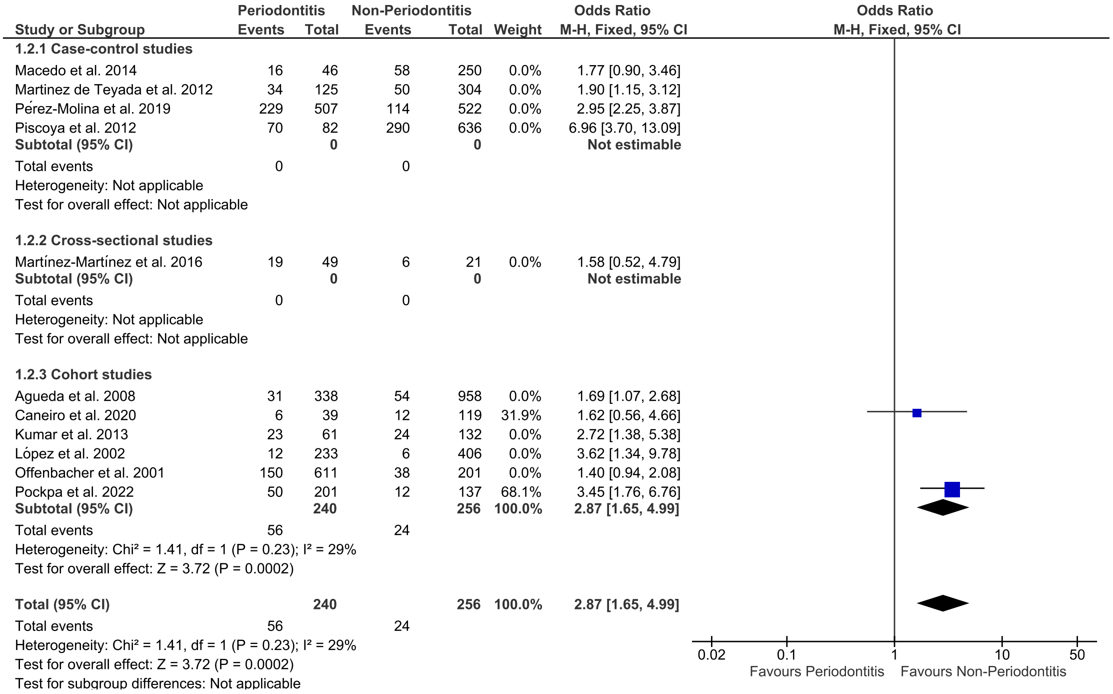

PPD > 3 mm (REM)

Study or Subgroup	Periodontitis Events	Periodontitis Total	Non-Periodontitis Events	Non-Periodontitis Total	Weight	Odds Ratio M-H, Random, 95% CI
**1.2.1 Case-control studies**						
Macedo et al. 2014	16	46	58	250	11.9%	1.77 [0.90, 3.46]
Martinez de Teyada et al. 2012	34	125	50	304	0.0%	1.90 [1.15, 3.12]
Pérez-Molina et al. 2019	229	507	114	522	17.7%	2.95 [2.25, 3.87]
Piscoya et al. 2012	70	82	290	636	12.5%	6.96 [3.70, 13.09]
**Subtotal (95% CI)**		**635**		**1408**	**42.0%**	**3.30 [1.74, 6.26]**
Total events	315		462			
Heterogeneity: Tau² = 0.25; Chi² = 9.22, df = 2 (P = 0.010); I² = 78%						
Test for overall effect: Z = 3.64 (P = 0.0003)						
**1.2.2 Cross-sectional studies**						
Martínez-Martínez et al. 2016	19	49	6	21	7.1%	1.58 [0.52, 4.79]
**Subtotal (95% CI)**		**49**		**21**	**7.1%**	**1.58 [0.52, 4.79]**
Total events	19		6			
Heterogeneity: Not applicable						
Test for overall effect: Z = 0.81 (P = 0.42)						
**1.2.3 Cohort studies**						
Agueda et al. 2008	31	338	54	958	15.0%	1.69 [1.07, 2.68]
Caneiro et al. 2020	6	39	12	119	0.0%	1.62 [0.56, 4.66]
Kumar et al. 2013	23	61	24	132	11.8%	2.72 [1.38, 5.38]
López et al. 2002	12	233	6	406	8.1%	3.62 [1.34, 9.78]
Offenbacher et al. 2001	150	611	38	201	16.0%	1.40 [0.94, 2.08]
Pockpa et al. 2022	50	201	12	137	0.0%	3.45 [1.76, 6.76]
**Subtotal (95% CI)**		**1243**		**1697**	**50.9%**	**1.89 [1.31, 2.72]**
Total events	216		122			
Heterogeneity: Tau² = 0.05; Chi² = 4.94, df = 3 (P = 0.18); I² = 39%						
Test for overall effect: Z = 3.40 (P = 0.0007)						
**Total (95% CI)**		**1927**		**3126**	**100.0%**	**2.43 [1.68, 3.52]**
Total events	550		590			
Heterogeneity: Tau² = 0.18; Chi² = 24.69, df = 7 (P = 0.0009); I² = 72%						
Test for overall effect: Z = 4.71 (P < 0.00001)						
Test for subgroup differences: Chi² = 2.47, df = 2 (P = 0.29), I² = 19.1%						

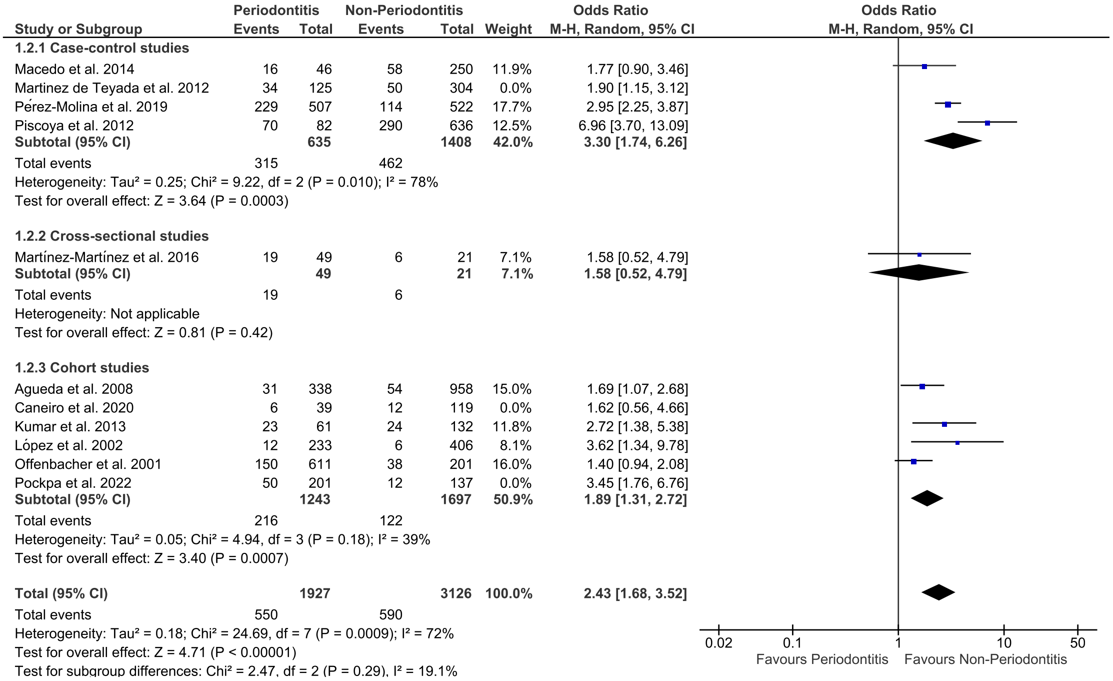

PPD > 2 mm (FEM)

Study or Subgroup	Periodontitis Events	Periodontitis Total	Non-Periodontitis Events	Non-Periodontitis Total	Weight	Odds Ratio M-H, Fixed, 95% CI
**1.2.1 Case-control studies**						
Macedo et al. 2014	16	46	58	250	0.0%	1.77 [0.90, 3.46]
Martinez de Teyada et al. 2012	34	125	50	304	0.0%	1.90 [1.15, 3.12]
Pérez-Molina et al. 2019	229	507	114	522	0.0%	2.95 [2.25, 3.87]
Piscoya et al. 2012	70	82	290	636	0.0%	6.96 [3.70, 13.09]
**Subtotal (95% CI)**		**0**		**0**		**Not estimable**
Total events	0		0			
Heterogeneity: Not applicable						
Test for overall effect: Not applicable						
**1.2.2 Cross-sectional studies**						
Martínez-Martínez et al. 2016	19	49	6	21	0.0%	1.58 [0.52, 4.79]
**Subtotal (95% CI)**		**0**		**0**		**Not estimable**
Total events	0		0			
Heterogeneity: Not applicable						
Test for overall effect: Not applicable						
**1.2.3 Cohort studies**						
Agueda et al. 2008	31	338	54	958	0.0%	1.69 [1.07, 2.68]
Caneiro et al. 2020	6	39	12	119	31.9%	1.62 [0.56, 4.66]
Kumar et al. 2013	23	61	24	132	0.0%	2.72 [1.38, 5.38]
López et al. 2002	12	233	6	406	0.0%	3.62 [1.34, 9.78]
Offenbacher et al. 2001	150	611	38	201	0.0%	1.40 [0.94, 2.08]
Pockpa et al. 2022	50	201	12	137	68.1%	3.45 [1.76, 6.76]
**Subtotal (95% CI)**		**240**		**256**	**100.0%**	**2.87 [1.65, 4.99]**
Total events	56		24			
Heterogeneity: Chi² = 1.41, df = 1 (P = 0.23); I² = 29%						
Test for overall effect: Z = 3.72 (P = 0.0002)						
**Total (95% CI)**		**240**		**256**	**100.0%**	**2.87 [1.65, 4.99]**
Total events	56		24			
Heterogeneity: Chi² = 1.41, df = 1 (P = 0.23); I² = 29%						
Test for overall effect: Z = 3.72 (P = 0.0002)						
Test for subgroup differences: Not applicable						

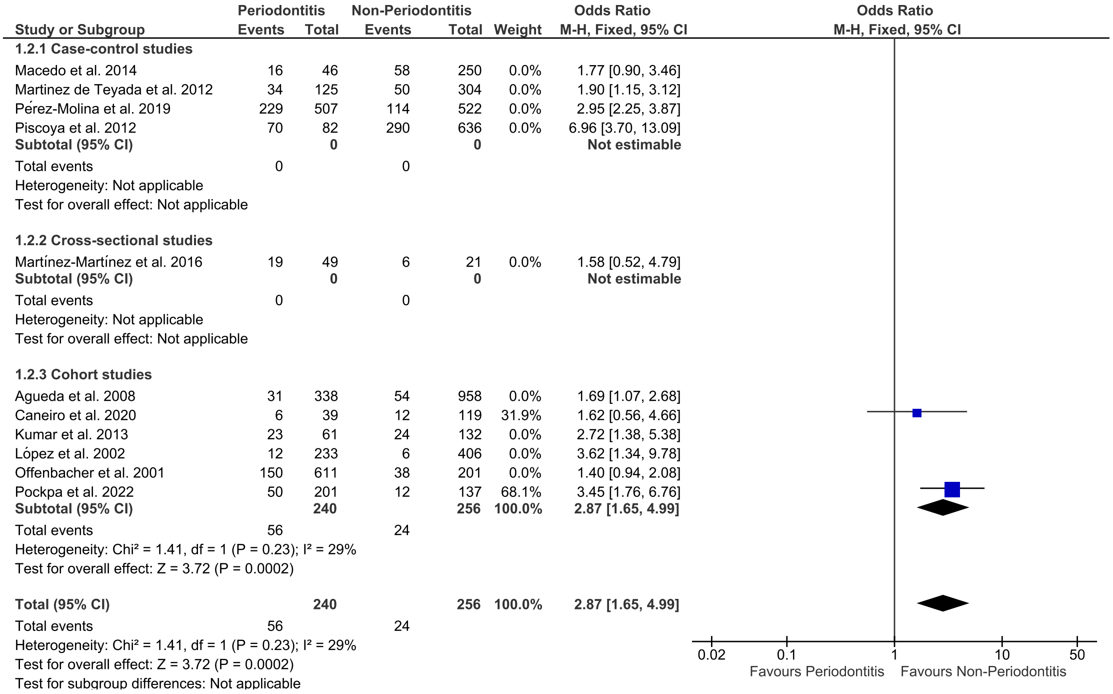

≥ 2 affected teeth (FEM)

Study or Subgroup	Periodontitis Events	Periodontitis Total	Non-Periodontitis Events	Non-Periodontitis Total	Weight	Odds Ratio M-H, Fixed, 95% CI
**1.2.1 Case-control studies**						
Macedo et al. 2014	16	46	58	250	0.0%	1.77 [0.90, 3.46]
Martinez de Teyada et al. 2012	34	125	50	304	57.4%	1.90 [1.15, 3.12]
Pérez-Molina et al. 2019	229	507	114	522	0.0%	2.95 [2.25, 3.87]
Piscoya et al. 2012	70	82	290	636	0.0%	6.96 [3.70, 13.09]
**Subtotal (95% CI)**		**125**		**304**	**57.4%**	**1.90 [1.15, 3.12]**
Total events	34		50			
Heterogeneity: Not applicable						
Test for overall effect: Z = 2.53 (P = 0.01)						
**1.2.2 Cross-sectional studies**						
Martínez-Martínez et al. 2016	19	49	6	21	0.0%	1.58 [0.52, 4.79]
**Subtotal (95% CI)**		**0**		**0**		**Not estimable**
Total events	0		0			
Heterogeneity: Not applicable						
Test for overall effect: Not applicable						
**1.2.3 Cohort studies**						
Agueda et al. 2008	31	338	54	958	0.0%	1.69 [1.07, 2.68]
Caneiro et al. 2020	6	39	12	119	13.6%	1.62 [0.56, 4.66]
Kumar et al. 2013	23	61	24	132	0.0%	2.72 [1.38, 5.38]
López et al. 2002	12	233	6	406	0.0%	3.62 [1.34, 9.78]
Offenbacher et al. 2001	150	611	38	201	0.0%	1.40 [0.94, 2.08]
Pockpa et al. 2022	50	201	12	137	29.0%	3.45 [1.76, 6.76]
**Subtotal (95% CI)**		**240**		**256**	**42.6%**	**2.87 [1.65, 4.99]**
Total events	56		24			
Heterogeneity: Chi² = 1.41, df = 1 (P = 0.23); I² = 29%						
Test for overall effect: Z = 3.72 (P = 0.0002)						
**Total (95% CI)**		**365**		**560**	**100.0%**	**2.31 [1.60, 3.34]**
Total events	90		74			
Heterogeneity: Chi² = 2.40, df = 2 (P = 0.30); I² = 17%						
Test for overall effect: Z = 4.47 (P < 0.00001)						
Test for subgroup differences: Chi² = 1.18, df = 1 (P = 0.28), I² = 15.0%						

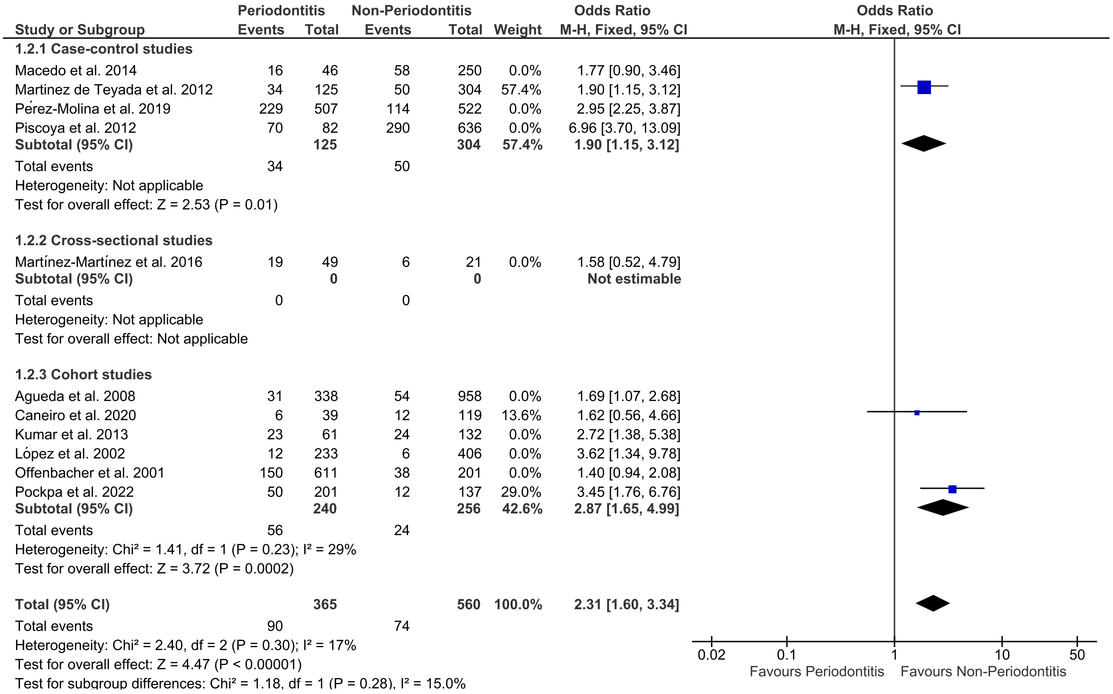

≥ 1 affected tooth (FEM)

Study or Subgroup	Periodontitis Events	Periodontitis Total	Non-Periodontitis Events	Non-Periodontitis Total	Weight	Odds Ratio M-H, Fixed, 95% CI
**1.2.1 Case-control studies**						
Macedo et al. 2014	16	46	58	250	0.0%	1.77 [0.90, 3.46]
Martinez de Teyada et al. 2012	34	125	50	304	0.0%	1.90 [1.15, 3.12]
Pérez-Molina et al. 2019	229	507	114	522	0.0%	2.95 [2.25, 3.87]
Piscoya et al. 2012	70	82	290	636	0.0%	6.96 [3.70, 13.09]
**Subtotal (95% CI)**		**0**		**0**		**Not estimable**
Total events	0		0			
Heterogeneity: Not applicable						
Test for overall effect: Not applicable						
**1.2.2 Cross-sectional studies**						
Martínez-Martínez et al. 2016	19	49	6	21	0.0%	1.58 [0.52, 4.79]
**Subtotal (95% CI)**		**0**		**0**		**Not estimable**
Total events	0		0			
Heterogeneity: Not applicable						
Test for overall effect: Not applicable						
**1.2.3 Cohort studies**						
Agueda et al. 2008	31	338	54	958	0.0%	1.69 [1.07, 2.68]
Caneiro et al. 2020	6	39	12	119	0.0%	1.62 [0.56, 4.66]
Kumar et al. 2013	23	61	24	132	18.0%	2.72 [1.38, 5.38]
López et al. 2002	12	233	6	406	0.0%	3.62 [1.34, 9.78]
Offenbacher et al. 2001	150	611	38	201	82.0%	1.40 [0.94, 2.08]
Pockpa et al. 2022	50	201	12	137	0.0%	3.45 [1.76, 6.76]
**Subtotal (95% CI)**		**672**		**333**	**100.0%**	**1.63 [1.16, 2.31]**
Total events	173		62			
Heterogeneity: Chi² = 2.76, df = 1 (P = 0.10); I² = 64%						
Test for overall effect: Z = 2.78 (P = 0.005)						
**Total (95% CI)**		**672**		**333**	**100.0%**	**1.63 [1.16, 2.31]**
Total events	173		62			
Heterogeneity: Chi² = 2.76, df = 1 (P = 0.10); I² = 64%						
Test for overall effect: Z = 2.78 (P = 0.005)						
Test for subgroup differences: Not applicable						

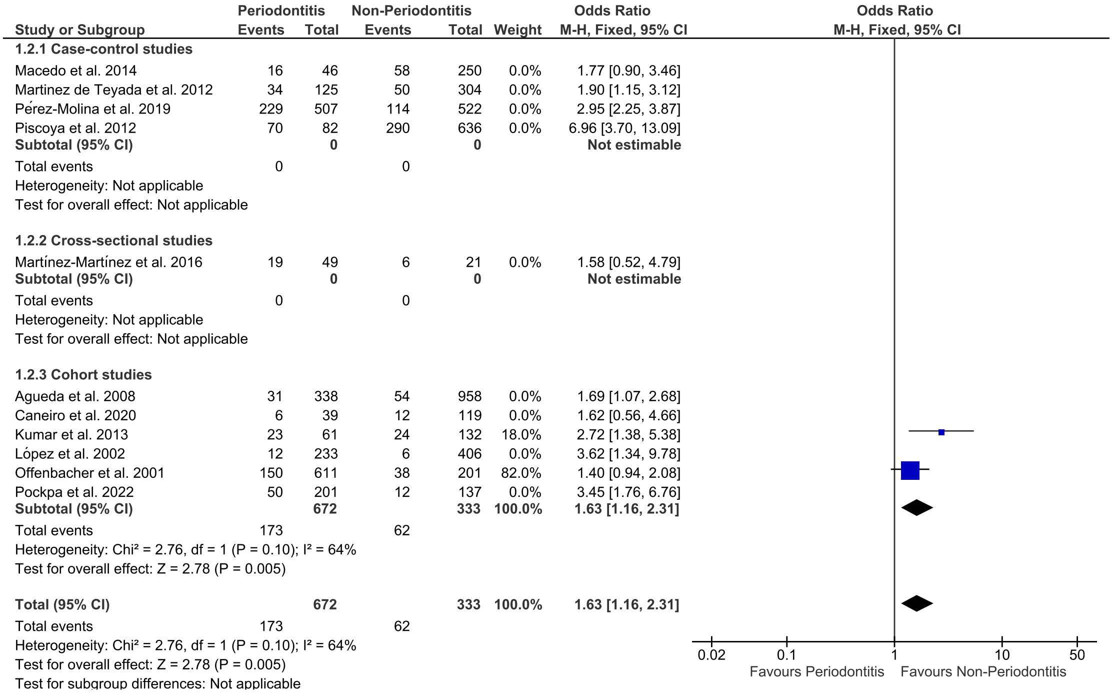

Online Appendix S3-G

Meta-analysis, subgroup analysis: forest plots using a random model (REM) of the performed meta-analysis for pregnant woment with periodontitis compared to pregnant women without periodontitis on number of examined teeth per participant.

Full mouth examination excluding third molars (REM)

Study or Subgroup	Periodontitis Events	Periodontitis Total	Non-Periodontitis Events	Non-Periodontitis Total	Weight	Odds Ratio M-H, Random, 95% CI
**1.2.1 Case-control studies**						
Macedo et al. 2014	16	46	58	250	15.6%	1.77 [0.90, 3.46]
Martinez de Teyada et al. 2012	34	125	50	304	28.7%	1.90 [1.15, 3.12]
Pérez-Molina et al. 2019	229	507	114	522	0.0%	2.95 [2.25, 3.87]
Piscoya et al. 2012	70	82	290	636	0.0%	6.96 [3.70, 13.09]
**Subtotal (95% CI)**		**171**		**554**	**44.4%**	**1.85 [1.24, 2.76]**
Total events	50		108			
Heterogeneity: Tau² = 0.00; Chi² = 0.03, df = 1 (P = 0.87); I² = 0%						
Test for overall effect: Z = 3.01 (P = 0.003)						
**1.2.2 Cross-sectional studies**						
Martínez-Martínez et al. 2016	19	49	6	21	0.0%	1.58 [0.52, 4.79]
**Subtotal (95% CI)**		**0**		**0**		**Not estimable**
Total events	0		0			
Heterogeneity: Not applicable						
Test for overall effect: Not applicable						
**1.2.3 Cohort studies**						
Agueda et al. 2008	31	338	54	958	33.5%	1.69 [1.07, 2.68]
Caneiro et al. 2020	6	39	12	119	6.4%	1.62 [0.56, 4.66]
Kumar et al. 2013	23	61	24	132	0.0%	2.72 [1.38, 5.38]
López et al. 2002	12	233	6	406	0.0%	3.62 [1.34, 9.78]
Offenbacher et al. 2001	150	611	38	201	0.0%	1.40 [0.94, 2.08]
Pockpa et al. 2022	50	201	12	137	15.7%	3.45 [1.76, 6.76]
**Subtotal (95% CI)**		**578**		**1214**	**55.6%**	**2.13 [1.30, 3.48]**
Total events	87		78			
Heterogeneity: Tau² = 0.07; Chi² = 3.20, df = 2 (P = 0.20); I² = 38%						
Test for overall effect: Z = 2.99 (P = 0.003)						
**Total (95% CI)**		**749**		**1768**	**100.0%**	**1.96 [1.50, 2.56]**
Total events	137		186			
Heterogeneity: Tau² = 0.00; Chi² = 3.37, df = 4 (P = 0.50); I² = 0%						
Test for overall effect: Z = 4.96 (P < 0.00001)						
Test for subgroup differences: Chi² = 0.18, df = 1 (P = 0.67), I² = 0%						

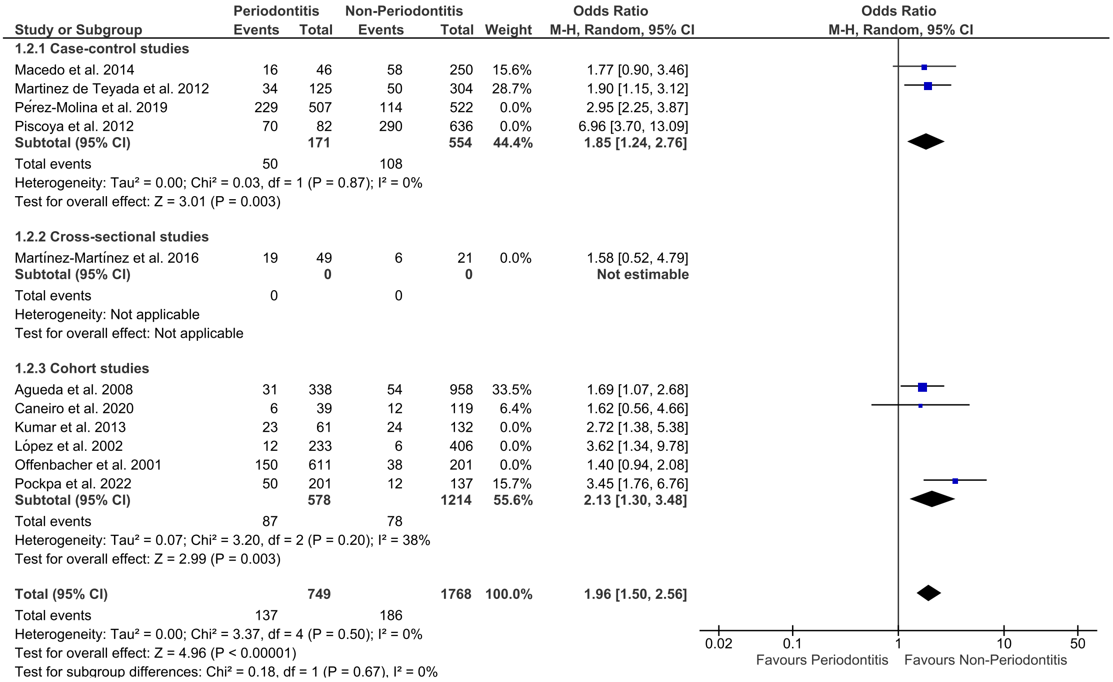

Full mouth examination without specification on third molars (REM)

Study or Subgroup	Periodontitis Events	Periodontitis Total	Non-Periodontitis Events	Non-Periodontitis Total	Weight	Odds Ratio M-H, Random, 95% CI
**1.2.1 Case-control studies**						
Macedo et al. 2014	16	46	58	250	0.0%	1.77 [0.90, 3.46]
Martinez de Teyada et al. 2012	34	125	50	304	0.0%	1.90 [1.15, 3.12]
Pérez-Molina et al. 2019	229	507	114	522	22.9%	2.95 [2.25, 3.87]
Piscoya et al. 2012	70	82	290	636	17.2%	6.96 [3.70, 13.09]
**Subtotal (95% CI)**		**589**		**1158**	**40.1%**	**4.31 [1.86, 10.01]**
Total events	299		404			
Heterogeneity: Tau² = 0.31; Chi² = 6.07, df = 1 (P = 0.01); I² = 84%						
Test for overall effect: Z = 3.40 (P = 0.0007)						
**1.2.2 Cross-sectional studies**						
Martínez-Martínez et al. 2016	19	49	6	21	10.5%	1.58 [0.52, 4.79]
**Subtotal (95% CI)**		**49**		**21**	**10.5%**	**1.58 [0.52, 4.79]**
Total events	19		6			
Heterogeneity: Not applicable						
Test for overall effect: Z = 0.81 (P = 0.42)						
**1.2.3 Cohort studies**						
Agueda et al. 2008	31	338	54	958	0.0%	1.69 [1.07, 2.68]
Caneiro et al. 2020	6	39	12	119	0.0%	1.62 [0.56, 4.66]
Kumar et al. 2013	23	61	24	132	16.4%	2.72 [1.38, 5.38]
López et al. 2002	12	233	6	406	11.9%	3.62 [1.34, 9.78]
Offenbacher et al. 2001	150	611	38	201	21.1%	1.40 [0.94, 2.08]
Pockpa et al. 2022	50	201	12	137	0.0%	3.45 [1.76, 6.76]
**Subtotal (95% CI)**		**905**		**739**	**49.3%**	**2.14 [1.18, 3.88]**
Total events	185		68			
Heterogeneity: Tau² = 0.16; Chi² = 4.89, df = 2 (P = 0.09); I² = 59%						
Test for overall effect: Z = 2.51 (P = 0.01)						
**Total (95% CI)**		**1543**		**1918**	**100.0%**	**2.77 [1.72, 4.44]**
Total events	503		478			
Heterogeneity: Tau² = 0.24; Chi² = 20.78, df = 5 (P = 0.0009); I² = 76%						
Test for overall effect: Z = 4.20 (P < 0.0001)						
Test for subgroup differences: Chi² = 2.53, df = 2 (P = 0.28), I² = 20.9%						

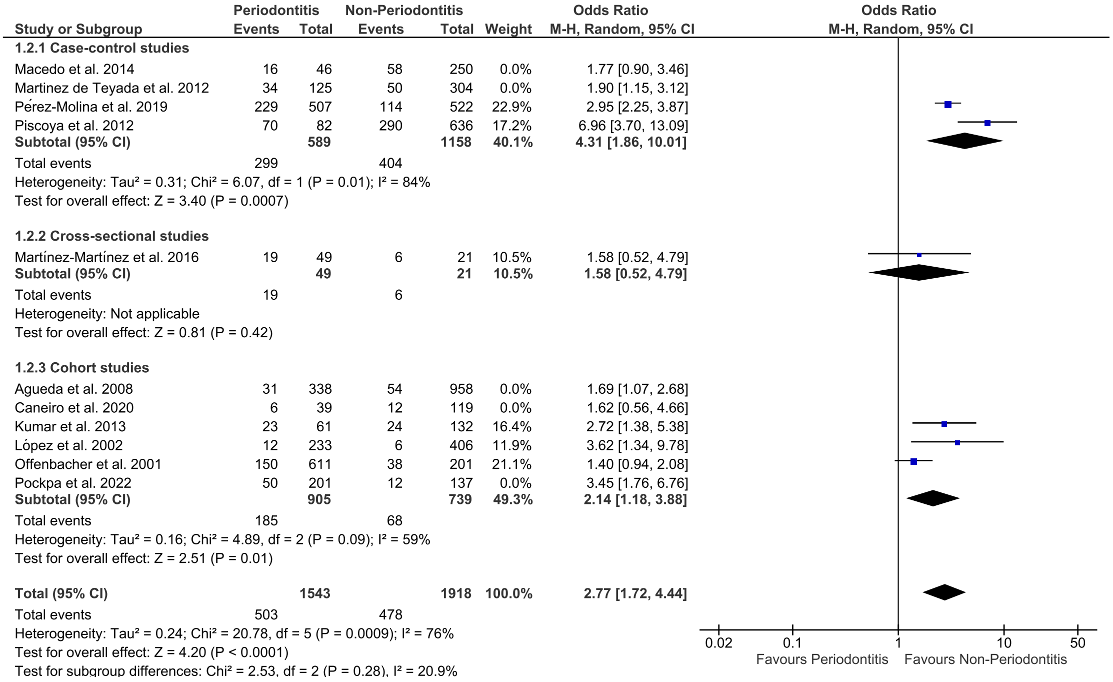

Online Appendix S4

Funnel plot of the selected studies

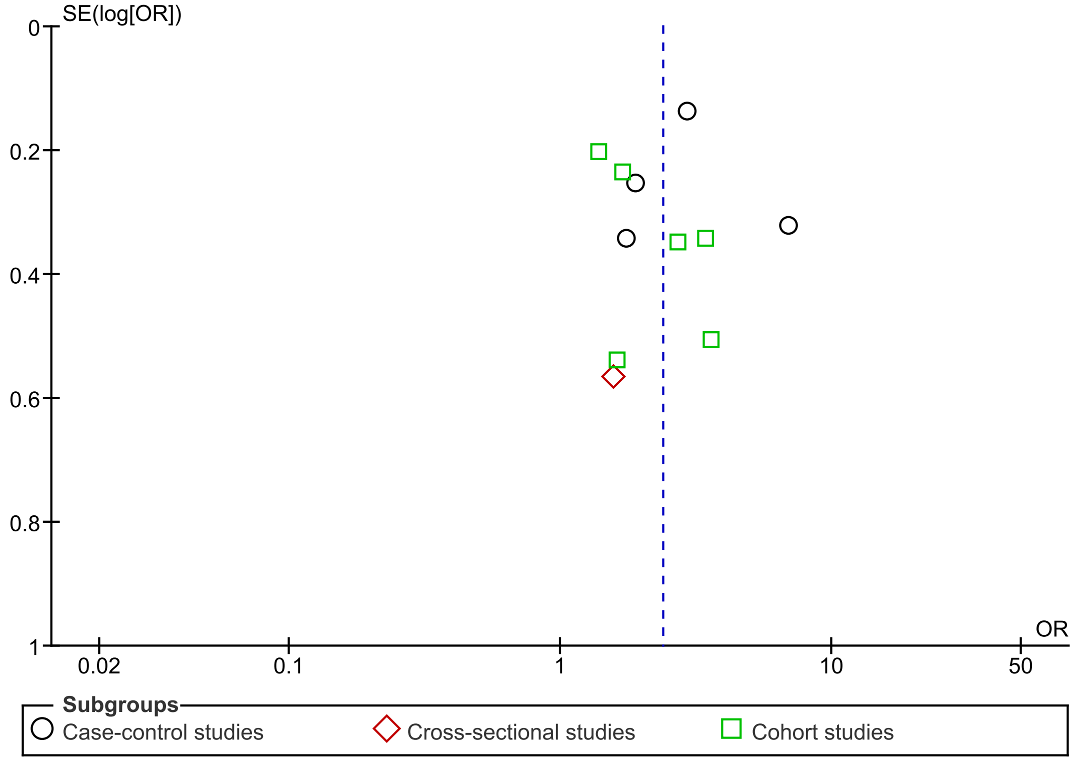

Online Appendix S5

TSA of the selected low risk of bias studies

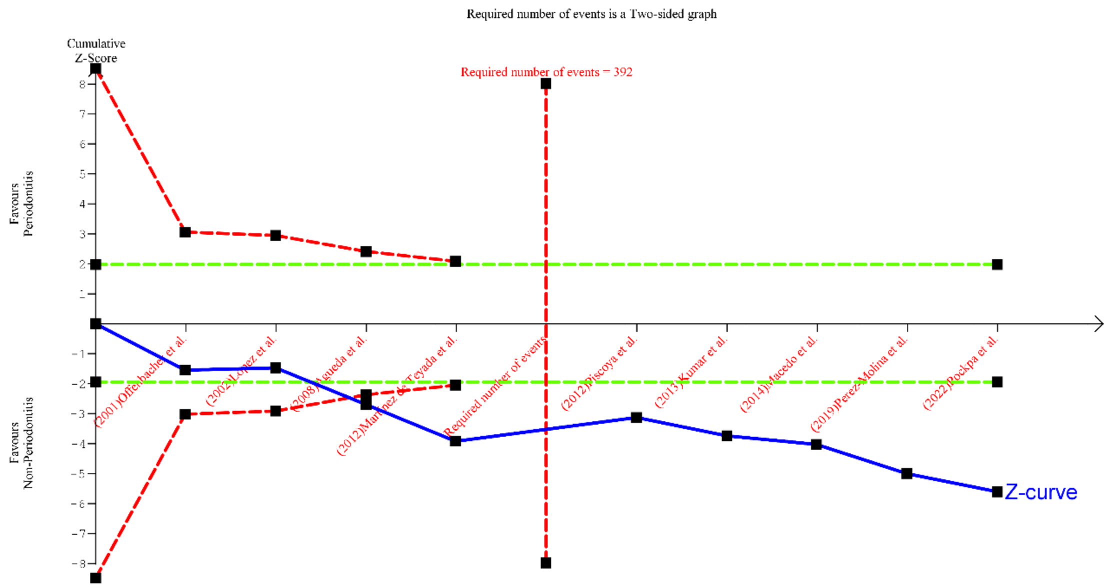

The cumulative blue Z-curves were constructed with each cumulative Z-value calculated after including a new trial according to publication date. Crossing of the two-sided Z = 1.96 provides a traditionally significant result. Crossing of the red trial sequential monitoring boundaries is needed to obtain reliable evidence adjusted for random error risk. Z-curves not crossing Z= 1.96 indicate absence of evidence if the information size is not reached or lack of the predefined intervention effect if the information size is not reached. The green dotted lines represent the traditional boundary. The vertical red line represents the estimated heterogeneity-adjusted required information size, the number of events for the meta-analysis sample size.
